# Adverse effects of 21 antidepressants on sleep during acute-phase treatment in major depressive disorder: a systemic review and dose-effect network meta-analysis

**DOI:** 10.1093/sleep/zsad177

**Published:** 2023-07-09

**Authors:** Shuzhe Zhou, Pei Li, Xiaozhen Lv, Xuefeng Lai, Zuoxiang Liu, Junwen Zhou, Fengqi Liu, Yiming Tao, Meng Zhang, Xin Yu, Jingwei Tian, Feng Sun

**Affiliations:** Peking University Sixth Hospital, Peking University Institute of Mental Health, Beijing, China; NHC Key Laboratory of Mental Health (Peking University), National Clinical Research Center for Mental Disorders (Peking University Sixth Hospital), Beijing, China; Department of Epidemiology and Biostatistics, School of Public Health, Peking University, Beijing, China; Peking University Sixth Hospital, Peking University Institute of Mental Health, Beijing, China; NHC Key Laboratory of Mental Health (Peking University), National Clinical Research Center for Mental Disorders (Peking University Sixth Hospital), Beijing, China; Department of Epidemiology and Biostatistics, School of Public Health, Peking University, Beijing, China; Department of Epidemiology and Biostatistics, School of Public Health, Peking University, Beijing, China; Department of Epidemiology and Biostatistics, School of Public Health, Peking University, Beijing, China; Department of Epidemiology and Biostatistics, School of Public Health, Peking University, Beijing, China; Department of Epidemiology and Biostatistics, School of Public Health, Peking University, Beijing, China; Department of Epidemiology and Biostatistics, School of Public Health, Peking University, Beijing, China; Peking University Sixth Hospital, Peking University Institute of Mental Health, Beijing, China; NHC Key Laboratory of Mental Health (Peking University), National Clinical Research Center for Mental Disorders (Peking University Sixth Hospital), Beijing, China; School of Pharmacy, Key Laboratory of Molecular Pharmacology and Drug Evaluation (Yantai University), Ministry of Education, Collaborative Innovation Center of Advanced Drug Delivery System and Biotech Drugs in Universities of Shandong, Yantai University, Yantai, China; Department of Epidemiology and Biostatistics, School of Public Health, Peking University, Beijing, China; Key Laboratory of Epidemiology of Major Diseases (Peking University), Ministry of Education, Beijing, China

**Keywords:** antidepressants, adverse effects, somnolence, insomnia, major depressive disorders

## Abstract

**Study Objectives:**

Sleep-related adverse effects during acute treatment with antidepressants undermine adherence and impede remission. We aimed to address subtypes of sleep-related adverse effects and depict the relationship between dose and sleep-related adverse events.

**Methods:**

We searched PubMed, Embase, Cochrane Central Register of Controlled Trials, and Web of Science for double-blind randomized controlled trials of depression published before April 30th, 2023. Eligible studies reporting sleep-related adverse effects during short-term monotherapy were included. The odds ratios (ORs) for sleep-related adverse effects were addressed with network meta-analysis. A Bayesian approach was used to depict the dose-effect relationship. Heterogeneity among studies was assessed using the τ^2^ and I^2^ statistics. Sensitivity analyses were performed without studies featuring high risk of bias.

**Results:**

Studies with 64 696 patients were examined from 216 trials. Compared to placebo, 13 antidepressants showed higher ORs for somnolence, of which fluvoxamine (OR = 6.32; 95% CI: 3.56 to 11.21) ranked the top. Eleven had higher risks for insomnia, reboxetine ranked the top (OR = 3.47; 95% CI: 2.77 to 4.36). The dose-effect relationships curves between somnolence or insomnia and dose included linear shape, inverted U-shape, and other shapes. There was no significant heterogeneity among individual studies. The quality of evidence for results in network meta-analyses was rated as very low to moderate by Grading of Recommendations Assessment, Development, and Evaluation.

**Conclusions:**

Most antidepressants had higher risks for insomnia or somnolence than placebo. The diverse relationship curves between somnolence or insomnia and dose of antidepressants can guide clinicians to adjust the doses. These findings suggest clinicians pay more attention to sleep-related adverse effects during acute treatment with antidepressants.

**Statement of Significance**

Sleep-related adverse effects reduce efficacy and acceptability of antidepressants for the acute treatment of patients with major depressive disorder, but are underreported in many clinical trials. We addressed the odd ratios of treatment-emergent somnolence and insomnia of antidepressants and depicted the relationship between dose and sleep-related adverse events. We found that most antidepressants had higher risks for insomnia or somnolence compared to placebo, among which fluvoxamine, trazodone, and mirtazapine ranked top three for somnolence and reboxetine, vilazodone, and desvenlafaxine ranked top three for insomnia. The dose-effect relationships curves between risks of somnolence or insomnia and doses of antidepressants not only appeared linear, but also appeared inverted U-shapes and other shapes. This complex dose-effect relationship requires more attention.

## Introduction

Major depressive disorder (MDD) is a common mood disorder, affecting about 20% of people worldwide, which is predicted to rank as the leading cause of global impact of psychiatric disease by 2030 [1]. Antidepressants are usually prescribed for MDD patients and are recommended as a first-line treatment for moderate and severe depression [2]. At present, more than 30 antidepressants are generally used in treatment of MDD, including selective serotonin reuptake inhibitors, serotonin and norepinephrine reuptake inhibitors, and others. Although most of these are effective, it is necessary for clinicians to balance their efficacy and acceptability [3]. There are many adverse effects commonly associated with antidepressants, including disordered sleep, sexual dysfunction, and gastrointestinal side effects, and these may result in discontinuation during the acute-phase treatment [2]. Reviews and meta-analyses on sexual dysfunction [4] and gastrointestinal side effects [5] were reported recently. Sleep-related adverse effects during short-term treatment with antidepressants not only undermine patient adherence but are also associated with an impediment to achieve remission, greater functional impairment, and higher risk of recurrence [6]. But meta-analysis on the sleep-related adverse effects is still scarce and the prevalence of treatment-emergent sleep disturbance in patients with MDD taking antidepressants is ambiguous.

Some systematic reviews have reported the influence of antidepressants on sleep architecture and physiology [7, 8], providing evidence that different antidepressants with different action mechanisms and pharmacokinetics may have different adverse effects on sleep [9]. Insomnia and somnolence have a significant influence on patients who require alertness in their work, which may include driving or operating heavy machinery, resulting in severe adverse events. A post-marketing adverse drug reaction study [10] assessed and ranked the odds ratios (ORs) of somnolence among 30 antidepressants under a wide array of clinical indications circumstances not limited to MDD. Some meta-analyses [11, 12] qualified and compared the rates of insomnia and somnolence associated with second-generation antidepressants during acute-phase treatment of MDD. Other sleep disorders, including nightmares, sleep terrors, restless leg syndrome, sleep paralysis, sleep-related hallucinations, and sleepwalking can also be found and affect clinicians’ choice of antidepressants [13]. Thus, it is important to clarify the association between antidepressants and adverse effects on sleep [7, 9]. A comprehensive comparative analysis that ranks the odds of adverse effects of common antidepressants on sleep during short-term treatment for MDD is an unmet clinical need. Network meta-analyses of datasets from high-quality double-blinded randomized controlled trials (RCTs) make it possible to measure the rates and risks of sleep-related adverse events and provide essential evidence for clinicians to conduct optimal treatment [3].

Dose-effect relationships of antidepressants have been reported in several studies [14–16], which show that efficacy is not always dose-dependent. The debate on whether higher doses are more efficacious or not is still ongoing, and the same happens with dose and sleep-related adverse effects.

For instance, different dosages of trazodone have different effects on sleep architecture [8]. Low doses of mirtazapine are often prescribed off-label for insomnia clinically. And its sedation effect might be attenuated at higher doses, probably due to increased serotonin and norepinephrine release [17]. It is essential for clinicians to identify the relationship between dosage and sleep-related adverse effects to make better use of antidepressants, but few studies focus on this aspect.

Our study was specifically designed to address the different subtypes of sleep-related adverse effects during acute treatment with antidepressants for patients living with MDD and depict the relationship between dose and these adverse events. To this end, we conducted a systematic review and dose-effect network meta-analysis.

## Methods

We reported this study according to the Preferred Reporting Items for Systematic Reviews and Meta-Analyses (PRISMA) [18]. The protocol was registered on PROSPERO, the International prospective register of systematic reviews (CRD42022339567).

### Data source and search strategy

We systematically searched multiple databases, including PubMed, Embase, the Cochrane Central Register of Controlled Trials (CENTRAL), and Web of Science, for articles published before April 30th, 2023. We also searched clinicaltrials.gov for unpublished trials. The reference lists of previous systematic reviews were screened to supplement study inclusion. The search terms included “depress,” “dysthymia,” and names of included antidepressants, among others. The detailed search strategy is described in Supplementary Table S1.

### Study selection and data extraction

Double-blinded RCTs involving adults (≥18 years) with MDD and that compared antidepressants with placebo, compared different antidepressants, or compared different doses of antidepressants were eligible. Studies reporting sleep-related adverse effects that occurred during the trial period were included. For the screened studies, we went through their method section and protocol, if applicable, to confirm all the investigated outcomes. If any sleep-related adverse effect was mentioned in the method section or protocol but not reported in the result, we consider zero events occurred for this outcome. According to the previous large-scale reviews [3, 4] and clinical practice, we included 21 antidepressants in the analysis of odds risks of sleep-related adverse effects and dose-effect network meta-analysis (see Table 1 and Table 2). Toludesvenlafaxine, a new triple reuptake inhibitor with a good effect profile [19, 20], was only included in the analysis of the risk of somnolence because of the limited study data, which were a revision from original protocol. Only monotherapy was considered. We excluded RCTs of women with postpartum depression, of patients with post-stroke depression, of participants that consisted of more than 20% of bipolar or psychotic depression, and of participants with resistant depression and concomitant medical illness.

**Table 1.** Summary of Finding Table for Somnolence

	Relative effect (95% CI)	Anticipated absolute effect (95% CI)			Certainty of evidence	Ranking
		*Placebo*	*Other strategies*	Difference		
Agomelatine (2 RCTs; 6543 participants)	OR 1.39 (1.06 to 1.82) Network estimate	45 per 1000	61 per 1000	16 more (3 more to 34 more)	moderate, due to within-study bias	7
Amitriptyline (2 RCTs; 1491 participants)	OR 3.84 (2.81 to 5.23) Network estimate	45 per 1000	173 per 1000	128 more (81 more to 190 more)	moderate, due to within-study bias	20
Bupropion (1 RCT; 936 participants)	OR 0.50 (0.30 to 0.82) Network estimate	45 per 1000	23 per 1000	23 fewer (31 fewer to 8 fewer)	moderate, due to within-study bias	1
Citalopram (1 RCT; 1498 participants)	OR 1.38 (0.91 to 2.09) Network estimate	45 per 1000	62 per 1000	17 more (4 fewer to 49 more)	moderate, due to within-study bias	8
Clomipramine (2 RCTs; 55 participants)	OR 6.39 (0.24 to 172.51) Network estimate	45 per 1000	231 per 1000	186 more (34 fewer to 845 more)	very low, due to within-study bias, incoherence	18
Desvenlafaxine (1 RCT; 1116 participants)	OR 2.03 (1.20 to 3.43) Network estimate	45 per 1000	87 per 1000	42 more (9 more to 94 more)	moderate, due to within-study bias	10
Duloxetine (1 RCT; 3147 participants)	OR 3.02 (2.31 to 3.95) Network estimate	45 per 1000	125 per 1000	80 more (53 more to 112 more)	moderate, due to within-study bias	17
Escitalopram (2 RCT; 3284 participants)	OR 2.87 (2.04 to 4.04) Network estimate	45 per 1000	119 per 1000	74 more (43 more to 115 more)	moderate, due to within-study bias	15
Fluoxetine (1 RCT; 4246 participants)	OR 2.14 (1.70 to 2.69) Network estimate	45 per 1000	92 per 1000	47 more (29 more to 67 more)	moderate, due to within-study bias	12
Fluvoxamine (1 RCT; 295 participants)	OR 6.32 (3.56 to 11.21) Network estimate	45 per 1000	229 per 1000	184 more (99 more to 301 more)	very low, due to within-study bias, incoherence	23
Levomilnacipran (1 RCT; 85 participants)	OR 0.78 (0.15 to 4.03) Network estimate	45 per 1000	35 per 1000	10 fewer (38 fewer to 115 more)	very low, due to within-study bias, incoherence	3
Milnacipran (1 RCT; 932 participants)	OR 1.90 (1.07 to 3.37) Network estimate	45 per 1000	82 per 1000	37 more (3 more to 92 more)	very low, due to within-study bias, incoherence	9
Mirtazapine (1 RCT; 807 participants)	OR 4.47 (3.00 to 6.66) Network estimate	45 per 1000	174 per 1000	129 more (79 more to 194 more)	very low, due to within-study bias, incoherence	21
Nefazodone (1 RCT; 217 participants)	OR 2.37 (1.29 to 4.35) Network estimate	45 per 1000	100 per 1000	55 more (12 more to 125 more)	very low, due to within-study bias	14
Paroxetine (1 RCT; 5567 participants)	OR 2.83 (2.33 to 3.43) Network estimate	45 per 1000	118 per 1000	73 more (54 more to 94 more)	moderate, due to within-study bias	16
Reboxetine (1 RCT; 1094 participants)	OR 1.26 (0.81 to 1.98) Network estimate	45 per 1000	56 per 1000	11 more (8 fewer to 4 more)	moderate, due to within-study bias	6
Sertraline (2 RCT; 2280 participants)	OR 2.25 (1.65 to 3.08) Network estimate	45 per 1000	96 per 1000	51 more (27 more to 82 more)	moderate, due to within-study bias	13
Toludesvenlafaxine (1 RCT; 368 participants)	OR 7.66 (0.41 to 143.81) Network estimate	45 per 1000	265 per 1000	22 more (26 fewer to 826more)	low, due to, incoherence	19
Trazodone (1 RCT; 1066 participants)	OR 4.64 (3.17 to 6.81) Network estimate	45 per 1000	179 per 1000	134 more (85 more to 198 more)	low, due to within-study bias, heterogeneity	22
Venlafaxine (1 RCT; 4116 participants)	OR 2.04 (1.57 to 2.65) Network estimate	45 per 1000	88 per 1000	43 more (24 more to 66 more)	moderate, due to within-study bias	11
Vilazodone (1 RCT; 575 participants)	OR 1.05 (0.48 to 2.32) Network estimate	45 per 1000	47 per 1000	2 more (23 fewer to 54 more)	moderate, due to within-study bias	4
Vortioxetine (1 RCT; 1801 participants)	OR 1.17 (0.74 to 1.86) Network estimate	45 per 1000	52 per 1000	7 more (11 fewer to 36 more)	moderate, due to within-study bias	5
Placebo (1 RCT; 13863 participants)	Reference comparator	No estimable	No estimable	No estimable	Reference comparator	2

**Table 2.** Summary of Finding for Insomnia

	Relative effect (95% CI)	Anticipated absolute effect (95% CI)			Certainty of evidence	Ranking
		*Placebo*	*Other strategies*	Difference		
Agomelatine (2 RCTs; 4910 participants)	OR 0.98 (0.75 to 1.27) Network estimate	54 per 1000	53 per 1000	1 fewer (13 fewer to 14 more)	moderate, due to within-study bias	4
Amitriptyline (2 RCTs; 1561 participants)	OR 0.63 (0.42 to 0.92) Network estimate	54 per 1000	35 per 1000	19 fewer (31 fewer to 4 fewer)	moderate, due to within-study bias	1
Bupropion (1 RCT; 2274 participants)	OR 1.83 (1.42 to 2.36) Network estimate	54 per 1000	95 per 1000	41 more (21 more to 65 more)	moderate, due to within-study bias	17
Citalopram (1 RCT; 1341 participants)	OR 1.67 (1.16 to 2.41) Network estimate	54 per 1000	87 per 1000	33 more (9 more to 182 more)	moderate, due to within-study bias	14
Clomipramine (2 RCTs; 199 participants)	OR 2.11 (0.83 to 5.40) Network estimate	54 per 1000	107 per 1000	53 more (9 fewer to 182 more)	moderate, due to within-study bias	18
Desvenlafaxine (1 RCT; 1720 participants)	OR 2.12 (1.50 to 2.99) Network estimate	54 per 1000	108 per 1000	54 more (25 more to 92 more)	low, due to within-study bias, incoherence	20
Duloxetine (1 RCT; 2952 participants)	OR 1.96 (1.60 to 2.42) Network estimate	54 per 1000	101 per 1000	47 more (30 more to 67 more)	moderate, due to within-study bias	19
Escitalopram (2 RCT; 3409 participants)	OR 1.37 (1.08 to 1.75) Network estimate	54 per 1000	73 per 1000	19 more (4 more to 37 more)	moderate, due to within-study bias	10
Fluoxetine (1 RCT; 5007 participants)	OR 1.65 (1.40 to 1.93) Network estimate	54 per 1000	86 per 1000	32 more (20 more to 45 more)	moderate, due to within-study bias	13
Fluvoxamine (1 RCT; 273 participants)	OR 1.28 (0.75 to 2.21) Network estimate	54 per 1000	68 per 1000	14 more (13 fewer to 58 more)	moderate, due to within-study bias	9
Levomilnacipran (1 RCT; 1014 participants)	OR 1.42 (0.85 to 2.38) Network estimate	54 per 1000	74 per 1000	21 more (8 fewer to 65 more)	moderate, due to within-study bias	11
Milnacipran (1 RCT; 519 participants)	OR 0.82 (0.45 to 1.50) Network estimate	54 per 1000	45 per 1000	9 fewer (29 fewer to 25 more)	moderate, due to within-study bias	2
Mirtazapine (1 RCT; 571 participants)	OR 1.04 (0.65 to 1.65) Network estimate	54 per 1000	56 per 1000	2 more (18 fewer to 32 more)	moderate, due to within-study bias	6
Nefazodone (1 RCT; 255 participants)	OR 1.01 (0.57 to 1.81) Network estimate	54 per 1000	55 per 1000	1 fewer (22 fewer to 40 more)	low, due to within-study bias	7
Paroxetine (1 RCT; 4499 participants)	OR 1.47 (1.26 to 1.71) Network estimate	54 per 1000	77 per 1000	23 more (13 more to 35 more)	moderate, due to within-study bias	12
Reboxetine (1 RCT; 1676 participants)	OR 3.47 (2.77 to 4.36) Network estimate	54 per 1000	165 per 1000	111 more (83 more to 145 more)	moderate, due to within-study bias	22
Sertraline (2 RCT; 2446 participants)	OR 1.67 (1.36 to 2.05) Network estimate	54 per 1000	87 per 1000	33 more (18 more to 51 more)	moderate, due to within-study bias	15
Trazodone (1 RCT; 190 participants)	OR 0.80 (0.34 to 1.85) Network estimate	54 per 1000	44 per 1000	10 fewer (35 fewer to 42 more)	moderate, due to within-study bias	3
Venlafaxine (1 RCT; 3904 participants)	OR 1.77 (1.44 to 2.19) Network estimate	54 per 1000	92 per 1000	38 more (22 more to 57 more)	moderate, due to within-study bias	16
Vilazodone (1 RCT; 1084 participants)	OR 2.99 (1.78 to 5.03) Network estimate	54 per 1000	146 per 1000	92 more (38 more to 169 more)	moderate, due to within-study bias	21
Vortioxetine (1 RCT; 2695 participants)	OR 1.07 (0.77 to 1.49) Network estimate	54 per 1000	58 per 1000	4 more (12 fewer to 24 more)	moderate, due to within-study bias	8
Placebo (1 RCT; 15098 participants)	Reference comparator	No estimable	No estimable	No estimable	Reference comparator	5

We extracted the name and the dosage per day of the antidepressants, as well as the feature of the study populations, including sample size, baseline severity, percentage of females, and mean age. Basic information was also extracted, such as year of publication, first author, and study region. We summarized information on study design and result reporting to assess the risk of bias within each individual study.

The main outcomes that we focused on were somnolence and insomnia. Both self-reported and clinically confirmed treatment-emergent somnolence and insomnia were extracted. In addition, we also extracted the numbers of participants that experienced other subtypes of sleep-related adverse effects, including nightmares, restless leg syndrome, rapid eye movement sleep behavior disorder, and sleepwalking.

The risk of bias in each included study was assessed using the Cochrane risk of bias tool (RoB2.0) [21], which assesses the following domains: randomization, deviation from the intended interventions, missing outcome data, outcome measures, selection of the reported results, and overall bias. The quality of evidence for results in network meta-analyses was rated through the Grading of Recommendations Assessment, Development, and Evaluation (GRADE) process from five aspects, including risk of bias, imprecision, inconsistency, indirectness, and publication bias [22].

Two groups of reviewers selected the studies, extracted information, and conducted the quality assessment independently. Every group consisted of three reviews, who were master or PhD candidates (PL, JWZ, YMT and XFL, MZ, and FQL). We did not use any automation tool in this process. Data were double-checked across two groups who worked independently. Discrepancies were resolved by discussion or consultation with senior investigators (FS and XZL), both were associate professors and specialists.

### Statistical analyses

We conducted network meta-analyses using the frequentist method, where different doses of the same antidepressant were treated as a single treatment. A random effects model was used to calculate pooled ORs and their 95% confidence intervals (95% CI). We calculated the surface under the cumulative ranking curve (SUCRA) to rank different treatments. Heterogeneity among studies was assessed using the τ^2^ and I^2^ statistics. We performed a meta-regression using the Bayesian method instead of subgroup analysis to explore the potential interaction between the study characteristics and antidepressants, according to study region, mean age of the study population (≥50/<50 years), percentage of females, and baseline severity (depression rating score). This alternative analysis method was a modification of the original protocol which had been amended in PROSPERO. In the Bayesian models, the number of the chain was three and the number of total interactions per chain was 50 000. Global inconsistency was evaluated using a generalized Q test, while local inconsistencies were detected using a node split approach [23]. Funnel plots were drawn to check for publication bias. The transitivity assumption was assessed by comparing the baseline characteristics of populations with different treatment comparisons, and box plots were used to display the similarity of the baseline characteristics. Sensitivity analysis was carried out by excluding studies that were assessed to feature high risk of bias.

Using a Bayesian approach, we implemented model-based network meta-analyses to explore the dose-effect relationship between dose and the risks for sleep-related adverse effects [24]. We estimated the maximum effect (ORmax) and its 95% credible interval (95%CrI) using an Emax model [25]. Only fixed-dose arms were included in the dose-effect modeling.

We performed the statistical analyses with R 4.0.1, and mainly used the netmeta, gemtc, and model-based network meta-analysis dose packages. *p* < .05 was considered to indicate significance in all statistical tests.

## Results

**Figure 1.**
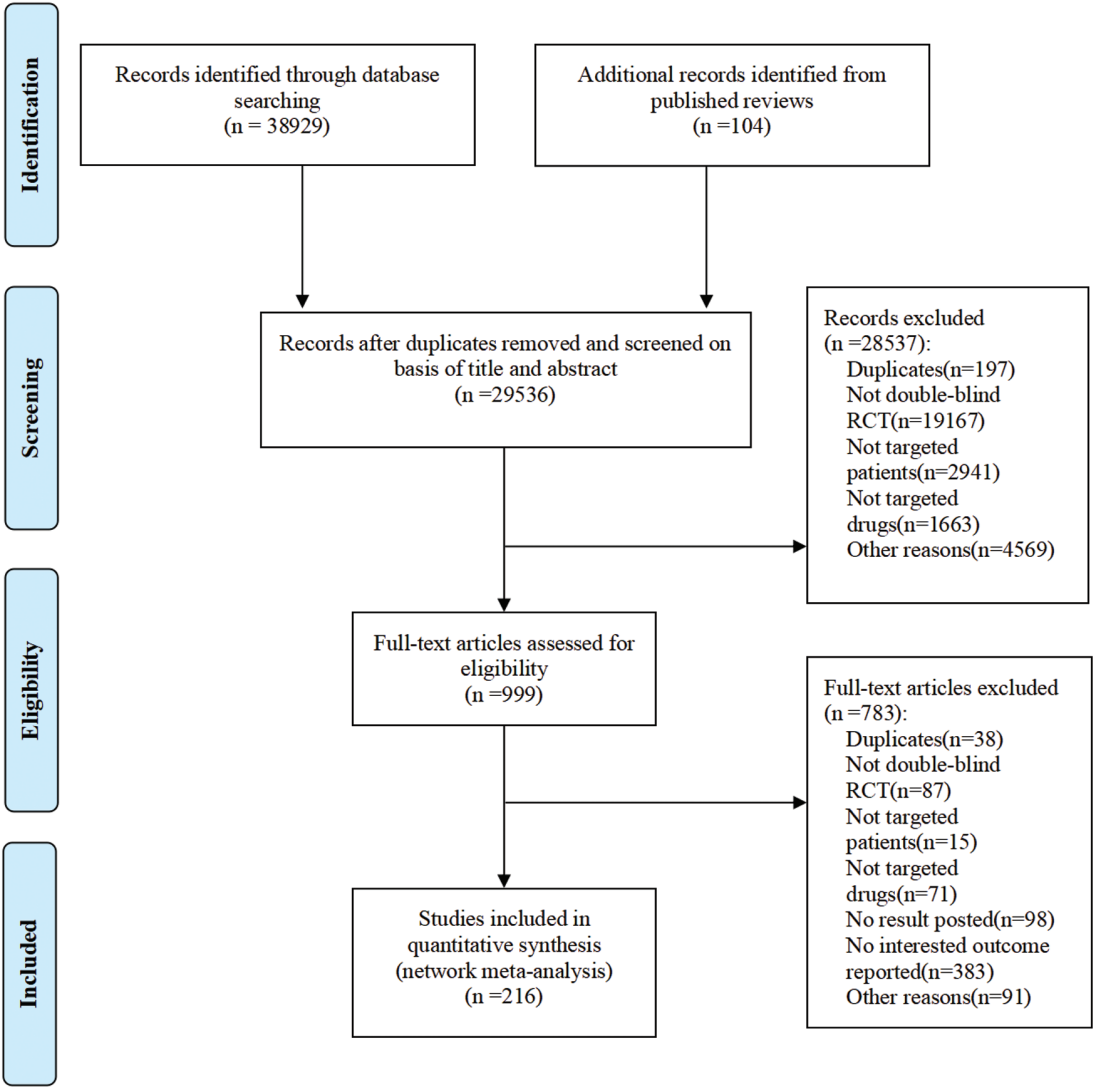
The flow diagram for study selection.

In all, 38 929 records were identified from the databases, and 104 studies were retrieved from the reference lists of published reviews. After screening the titles and abstracts, the full texts of 999 studies were reviewed. Following this, 216 studies were included in our systematic review, of which 163 were included in the meta-analyses for somnolence and 166 were included in the meta-analyses for insomnia (Figure 1). Other subtypes of sleep-related adverse effects were reported sparsely and could not be synthesized quantitively.

### Study characteristics

In total, 64 696 participants were enrolled in the included studies, of whom 63.2% were female, with a mean age of 45.5 years. Of the included studies, 127 (58.8%) studies involved a group of placebo controls, while 45 (20.8%) included a group treated with paroxetine, which is the most common active drug treatment among all 22 antidepressants. In addition, 85 (39.4%) studies were carried out in North America, 58 (26.9%) were conducted in Europe, 20 (9.7%) were from Asia, and 32 (14.8%) were carried out across multiple continents (detailed information on characteristics of individual studies can be viewed in Supplementary Table S2).

### Risk of bias and quality of evidence

**Figure 2.**
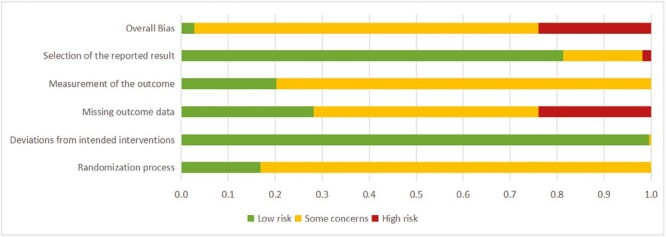
The result of risk of bias assessment.

Most studies (*n* = 158) were assessed to be of some concern in terms of overall bias. To be more specific, more than 80% of the included studies probably introduced risk of bias during the randomization process and outcome measurement. Because we only included double-blinded RCTs, there was a low risk of deviation from the intended interventions in the majority of the studies. Notably, 51 (23.6%) studies were at high risk for missing outcome data (Figure 2) (detailed information on risks for each study can be viewed in Supplementary Table S3).

### Results of network meta-analyses

**Figure 3.**
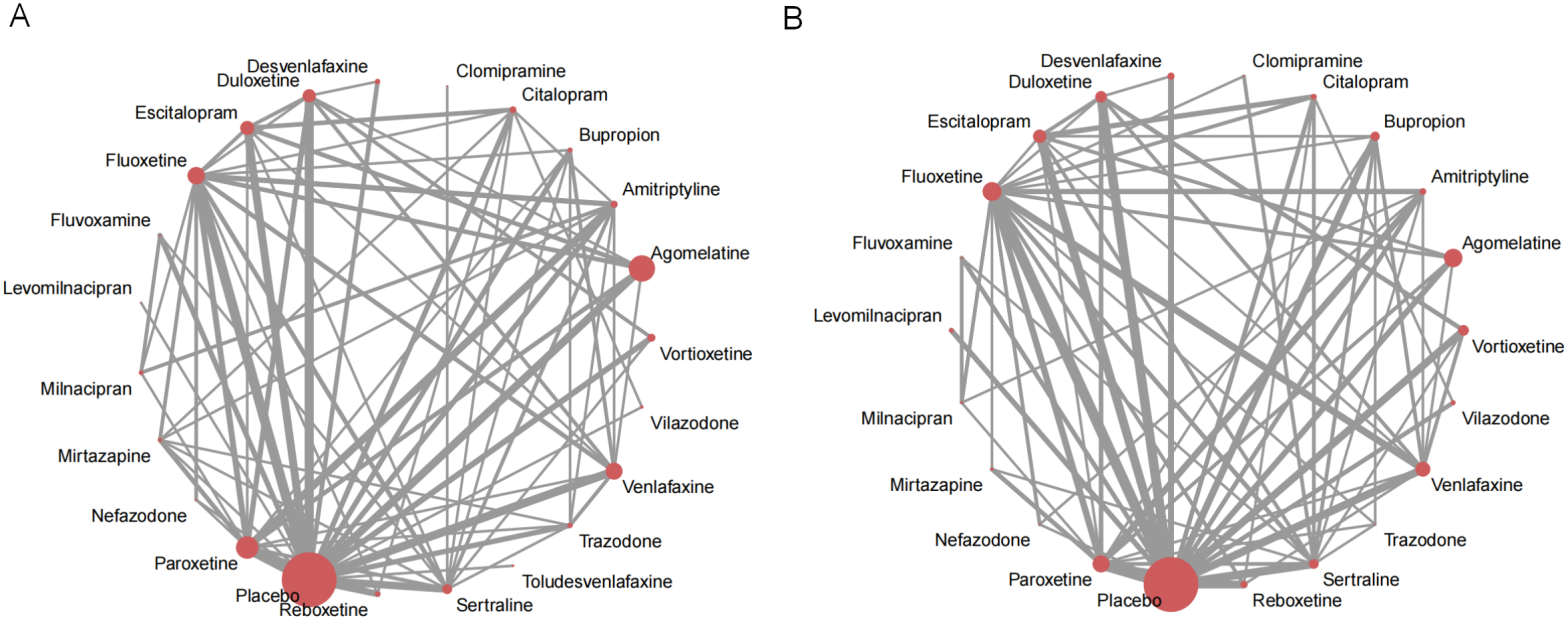
The network of evidence for main outcomes: (A) Somnolence; (B) Insomnia. The size of the nodes represents the sample size of each treatment group, and the width of the lines connecting different nodes is proportional to the number of RCTs comparing every pair of treatments.

**Figure 4.**
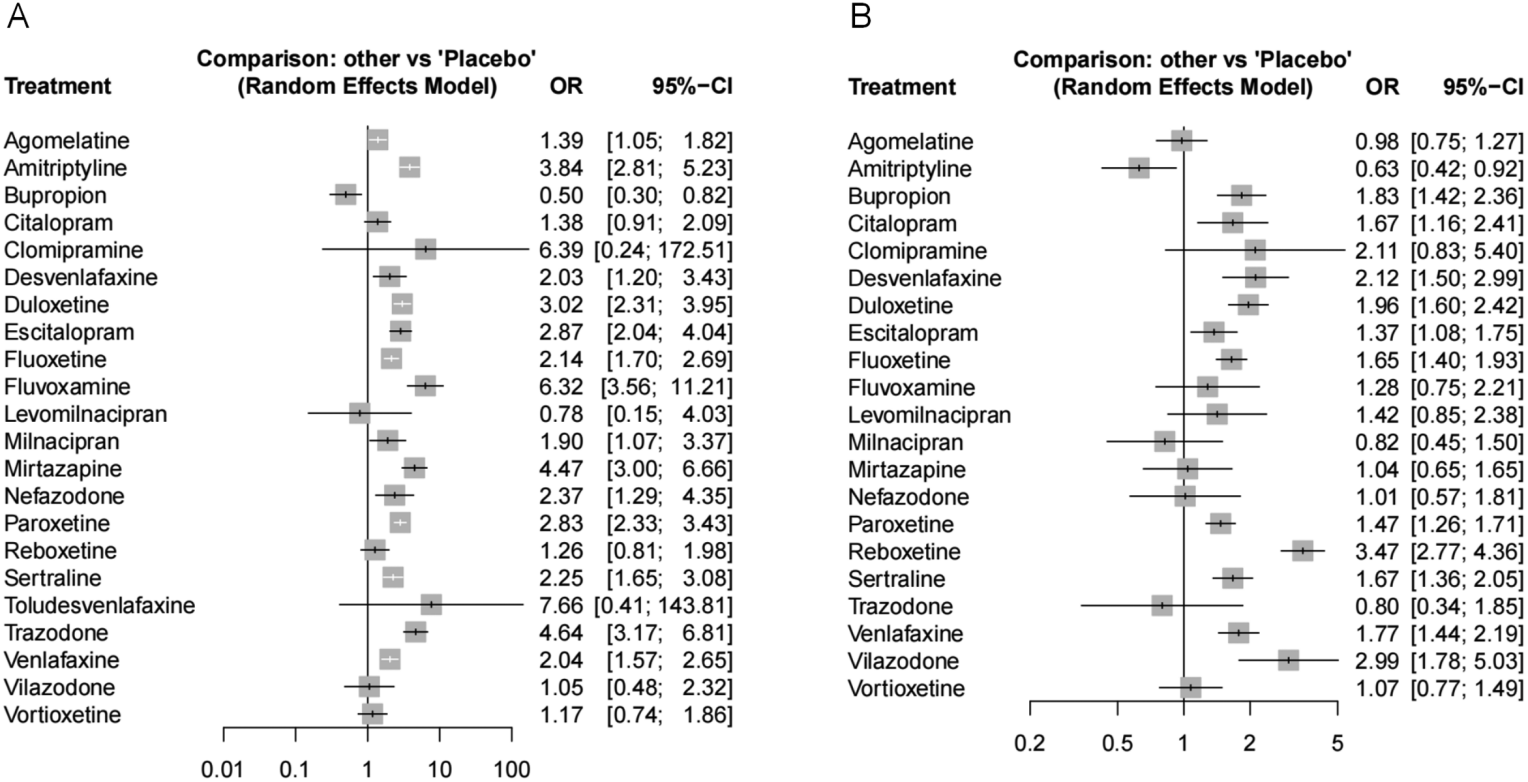
Forest plot displaying the result of network meta-analysis for main outcomes. (A) Somnolence; (B) Insomnia.

In all, 163 studies were included to construct a network for comparing the associations between different antidepressants and somnolence (Figure 3A). The prevalence of somnolence for placebo was approximately 4.5%. The estimated prevalence of somnolence for every antidepressant was calculated based on the OR of relative effect and the prevalence for placebo, such as agomelatine 6.1% and amitriptyline 17.3% (Table 1). Taking the placebo as a control group, fluvoxamine (OR = 6.32; 95% CI: 3.56 to 11.21), trazodone (OR = 4.64; 95% CI: 3.17 to 6.81), mirtazapine (OR = 4.47; 95% CI: 3.00 to 6.66), amitriptyline (OR = 3.84; 95% CI: 2.81 to 5.23), duloxetine (OR = 3.02; 95% CI: 2.31 to 3.95), escitalopram (OR = 2.87; 95% CI: 2.04 to 4.04), paroxetine (OR = 2.83; 95% CI: 2.33 to 3.43), nefazodone (OR = 2.37; 95% CI: 1.29 to 4.35), sertraline (OR = 2.25; 95% CI: 1.65 to 3.08), fluoxetine (OR = 2.14; 95% CI: 1.70 to 2.69), venlafaxine (OR = 2.04; 95% CI: 1.57 to 2.65), desvenlafaxine (OR = 2.03; 95% CI: 1.20 to 3.43), milnacipran (OR = 1.90; 95% CI: 1.07 to 3.37), and agomelatine (OR = 1.39; 95% CI: 1.05 to 1.82) were associated with a higher risk for somnolence. Bupropion (OR = 0.50; 95% CI: 0.30 to 0.82) had a lower risk for somnolence compared to placebo (Figure 4A).

The 166 studies that compared different antidepressants with respect to insomnia outcomes were included in a network (Figure 3B). The prevalence of insomnia for placebo was approximately 5.4%. The prevalence of insomnia for every antidepressant was calculated based on the OR of relative effect and the prevalence for placebo, such as agomelatine 5.3% and amitriptyline 3.5% (Table 2). Compared to placebo, reboxetine (OR = 3.47; 95% CI: 2.77 to 4.36), vilazodone (OR = 2.99; 95% CI: 1.78 to 5.03), desvenlafaxine (OR = 2.12; 95% CI: 1.50 to 2.99), duloxetine (OR = 1.96; 95% CI: 1.60 to 2.42), bupropion (OR = 1.83; 95% CI: 1.42 to 2.36), venlafaxine (OR = 1.77; 95% CI: 1.44 to 2.19), sertraline (OR=1.67; 95%CI: 1.36-2.05), citalopram (OR=1.67; 95%CI: 1.16-2.41), fluoxetine (OR = 1.65; 95% CI: 1.40 to 1.93), paroxetine (OR = 1.47; 95% CI: 1.26 to 1.71), and escitalopram (OR = 1.37; 95% CI: 1.08 to 1.75) had a higher risk for insomnia. However, amitriptyline (OR = 0.63; 95% CI: 0.42 to 0.92) was associated with a lower risk for insomnia than that of placebo (Figure 4B).

Heterogeneity among individual studies was not significant for somnolence or insomnia (τ^2^ = 0.098, I^2^ = 29.1% [95% CI: 13.2%~41.1%] and τ^2^ = 0.013, I^2^ = 5.7% [95% CI: 0.0%~22.5%], respectively). In the meta-regression, there was no significant interaction between baseline characteristics (mean age, percentage of females, and baseline severity) and antidepressants in terms of the risk for both main outcomes, except for gender, and baseline severity had an effect on toludesvenlafaxine (Supplementary Tables S4–S9). However, a significant global inconsistency was detected in the networks of both outcomes. The local inconsistency was explored using the node split approach (Supplementary Table S10). No publication bias was detected from the funnel plot (Supplementary Figures S3 and S4). Baseline characteristics, including the percentage of females, mean age, and baseline severity, were similar across different designs (Supplementary Figure S5). When studies with high risk of bias were excluded from the network, the results were consistent with the main analyses (Supplementary Figures S6 and S7).

Information on other subtypes of sleep-related adverse effects was presented in Supplementary Table S11. Sleep disorders, abnormal dreams, and yawning were the most commonly reported secondary outcomes, although the rates were very low.

The quality of evidence for results in network meta-analyses was rated as very low to moderate. The results of GRADE assessment are displayed in Table 1 and Table 2.

### Dose-effect relationship of each drug between dose and sleep-related adverse effects

**Figure 5.**
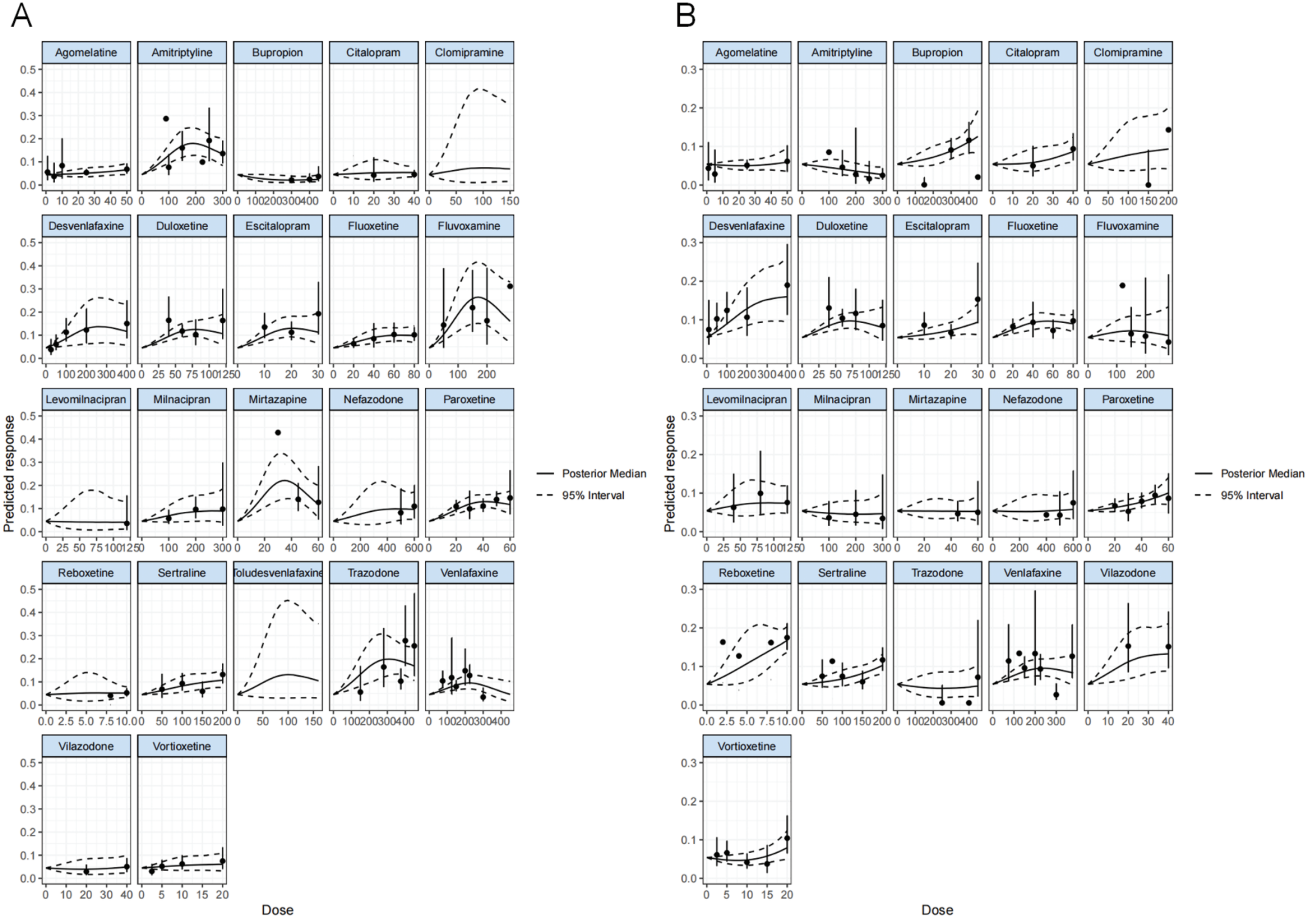
Dose-effect relationship of each drug between dose and sleep-related adverse events. (A) somnolence; (B) insomnia; *Y*-axis represents the absolute risk for somnolence or insomnia of different doses of antidepressants. *X*-axis represents doses of antidepressants, 0 represents placebo.

Dose-effect relationships of antidepressants between dose and sleep-related adverse events were presented in Figure 5. As for fluoxetine, milnacipran, nefazodone, and sertraline, the risks of somnolence increased linearly along with the dose increasing within conventional therapeutic doses. When it comes to amitriptyline, desvenlafaxine, duloxetine, escitalopram, paroxetine, toludesvenlafaxine, trazodone, and venlafaxine the risks of somnolence gradually increased from low doses to moderate doses and then showed a decreasing trend through the high doses. In regard to fluvoxamine and mirtazapine, the risks of somnolence were shown as an inverted U-shape, increasing steeply up to maximum effect and then decreasing. The maximum effect on somnolence of mirtazapine was at approximately 30 mg and fluvoxamine 150 mg (OR = 4.89; 95% CI: 3.12 to 9.40, OR = 6.41; 95% CI: 3.45 to 15.92, respectively).

Meanwhile, the risks of insomnia stayed flat from low doses to moderate doses and then increased through high doses of bupropion, citalopram, escitalopram, paroxetine, sertraline, and vortioxetine. With respect to desvenlafaxine, duloxetine, fluoxetine, venlafaxine, and vilazodone, the risks of insomnia increased gently and then presented a flat trend. Some antidepressants only appeared in one dose in the constructed model, and due to the limited sample size, some of the 95% CI were wide both in somnolence and insomnia.

Maximum effects were detected in the relationship between somnolence and doses of 21 antidepressants, 13 antidepressants had significantly higher risks than that placebo, not including milnacipran due to the wide 95% CI (OR = 1.68; 95% CI: 0.91 to 12.91). Regarding to maximum effects of insomnia, 11 antidepressants were estimated to have significantly higher risks of insomnia compared to placebo, which was consistent with the results of network meta-analysis. Bupropion (OR = 0.49; 95% CI: 0.28 to 0.83) had lower risks for somnolence even at maximum effects, whereas amitriptyline (OR = 0.63; 95% CI: 0.31 to 0.96) still had lower risks for insomnia compared to placebo (Table 3). The dose-effect models were fitted with the estimated effect size of the specific dose of antidepressants.

**Table 3.** The Odds Ratios of Somnolence and Insomnia of Antidepressants Compared to Placebo in Dose-Effect Models

Antidepressants	Somnolence	Insomnia
Agomelatine	1.42(1.00,12.04)	0.94(0.30,3.61)
Amitriptyline	4.04(2.84,6.19)	0.63(0.31,0.96)
Bupropion	0.49(0.28,0.83)	1.87(1.40,2.88)
Citalopram	1.34(0.85,21.52)	1.62(1.08,3.88)
Clomipramine	1.93(0.26,23.53)	1.64(0.51,5.14)
Desvenlafaxine	2.25(1.24,9.63)	2.33(1.60,5.86)
Duloxetine	3.23(2.39,5.31)	2.04(1.62,2.69)
Escitalopram	3.46(2.17,9.80)	1.48(1.00,3.39)
Fluoxetine	2.26(1.67,9.90)	1.68(1.41,2.19)
Fluvoxetine	6.41(3.45,15.92)	1.28(0.33,2.53)
Levomilnacipran	0.90(0.19,6.32)	1.40(0.72,2.71)
Milnacipran	1.68(0.91,12.91)	0.75(0.29,1.55)
Mirtazapine	4.89(3.12,9.40)	0.99(0.32,6.15)
Nefazodone	2.33(1.23,4.75)	1.03(0.49,1.84)
Paroxetine	3.05(2.34,5.82)	1.53(1.24,3.23)
Reboxetine	1.16(0.30,20.16)	3.69(2.75,5.90)
Sertraline	2.35(1.65,6.60)	1.69(1.32,7.68)
Toludesvenlafaxine	3.24(0.70,19.18)	—
Trazodone	5.29(3.53,13.77)	0.81(0.34,5.50)
Venlafaxine	2.06(1.55,2.80)	1.86(1.47,2.34)
Vilazodone	1.10(0.42,12.10)	2.68(1.68,5.21)
Vortioxetine	1.36(0.57,11.66)	1.13(0.25,5.79)

## Discussion

Our study investigated 216 double-blinded RCTs including 64 696 depressed patients to identify the ORs of insomnia and somnolence induced by different antidepressants. We found that fluvoxamine, trazodone, mirtazapine, amitriptyline, duloxetine, escitalopram, paroxetine, nefazodone, sertraline, fluoxetine, venlafaxine, desvenlafaxine, milnacipran, and agomelatine had higher ORs for somnolence and bupropion had a lower OR than placebo. For insomnia, reboxetine, vilazodone, desvenlafaxine, duloxetine, bupropion, venlafaxine, sertraline, citalopram, fluoxetine, paroxetine, and escitalopram had higher ORs and amitriptyline had a lower OR than placebo. But the risks of somnolence and insomnia were not always increased linearly along with the dose increase for 21 antidepressants.

With the exception of fluvoxamine, selective serotonin reuptake inhibitors and serotonin and norepinephrine reuptake inhibitors had higher risks for somnolence and insomnia than placebo. This finding is consistent with previous studies [2, 9] that reported that patients taking an SSRI or SNRI had similar likelihood of presenting with somnolence or insomnia. If patients complained of lethargy after taking an SSRI or SNRI in the morning, it was appropriate to administer it closer to bedtime. However, for fluvoxamine which had significantly higher risks for hypersomnia but not for insomnia than placebo, patients would benefit more by taking it at night. This might be related to the well-characterized ability of fluvoxamine to increase nocturnal serum levels of melatonin by 2- to 3-fold [26], with probable mechanism of inhibiting hepatic metabolism of melatonin by cytochrome P450 enzymes.

Agomelatine, mirtazapine, and trazodone are usually used to improve insomnia in depressed patients by changing their polysomnographic sleep architecture [8, 16]. Our results are consistent with the findings of previous studies. But these three antidepressants have different properties due to their different mechanisms. Agomelatine was given top acceptability in a previous network meta-analysis [3]. As a melatonin receptor agonist (MT1 and MT2), agomelatine can increase total sleep time, improve sleep efficiency [16], and restore circadian rhythm. Compared to mirtazapine, agomelatine has a lower frequency of oversedation or tiredness within 90 days of treatment [27]. Mirtazapine is a noradrenaline and specific serotonergic antidepressant that shows antagonism against the alpha-2 autoreceptor and heteroreceptors and strong antagonism against the 5-HT2, 5-HT3, and H1 receptors. In one study, mirtazapine was the most frequently associated with akathisia and restless leg syndrome, which can lead to difficulty falling asleep [28]. Mirtazapine might show optimal acceptability at 30 mg instead of 45 mg [29]. In a previous study, compared to the control group, somnolence, and dizziness occurred with greater frequency in the trazodone group [8], and a particularly complex action of antagonist on H1 histamine receptor, alpha 1, and alpha 2 adrenergic receptors of trazodone resulted in these unwanted side effects [30]. Whether trazodone should be first-line therapy for insomnia is still under discussion [31].

Vortioxetine, vilazodone, and levomilnacipran have been approved for the treatment of MDD in recent years. Insomnia is among the common adverse events, occurring at a rate of 6% for vilazodone and 5% for levomilnacipran [32]. Among all studies considered in our analysis, there were no significantly higher risk of vortioxetine for somnolence and insomnia comparing placebo [33]. Bupropion is a norepinephrine-dopamine disinhibitor. Insomnia is among the most commonly reported side effects associated with higher erythrohydrobupropion concentrations, and vivid dreams have also been reported [34]. Toludesvenlafaxine [19] is a new chemical compound that inhibits the reuptake of serotonin, norepinephrine, and dopamine, a triple reuptake inhibitor. Toludesvenlafaxine exerted good efficacy and acceptability in clinical trials [20]. In our study, the absolute risk of toludesvenlafaxine was shown to be low, which means we can anticipate low rate of somnolence related to toludesvenlafaxine. The relative risk of it was shown to be high with wide range of confidence intervals, these inconsistent results might be caused by the limited number of RCTs regarding toludesvenlafaxine.

Our study detected maximum effects between sleep-related adverse events and dose of antidepressants in addition to analyzing average effects. We find that the risks for somnolence and dose are not always linearly related, the same happens for insomnia and dose, which is rarely studied. In the linear relationship between somnolence and dose of fluoxetine, milnacipran, nefazodone, and sertraline, we expect somnolence more likely to happen with higher dose. The dose-effect relationship of fluvoxamine and mirtazapine exhibited an inverted U-shape. When patients taking mirtazapine 30 mg daily complain of daytime sleepiness, both decreasing doses and increasing doses could reduce the likelihood of somnolence. These findings might partly explain the non-linear dose–response curves in antidepressants [14, 35] and different uses with dosages [8, 17]. This finding provides an important perspective for clinicians to balance efficacy and safety and make the optimal choice.

Comparing the risks of treatment-emergent insomnia and somnolence and the dose-effect relationship, our study may help produce deeper insight into sleep-related adverse effects during acute treatment with antidepressants. We only enrolled RCTs and monotherapy studies, and the method of network meta-analysis increased the credibility of our findings. The search strategy and eligibility criteria were basically consistent with previously published systematic reviews [3, 29]. This approach will translate into a more systematic collection of available findings, facilitating the contextualization of these findings. However, there were some limits to our study. We excluded some patients characterized by postpartum depression and post-stroke depression, and we excluded RCTs with participant pools that consisted of more than 20% of cases of psychotic depression, limiting the applicability of the results but strengthening methodological transitivity. In addition, due to the paucity of qualified studies, other sleep disorders induced by antidepressants were not synthesized quantitively. Thirdly, both somnolence and insomnia are not primary and even secondary outcomes in most clinical trials in MDD and therefore are underreported. Studies investigating any outcome of sleep-related adverse effects but not reporting data were included in our study, considering zero events occurred for this outcome. Fourthly, the quality of evidence for some results in network meta-analyses was rated as very low and low by GRADE, which should be interpreted with caution.

In summary, our study sheds light on the frequency of sleep disturbances induced by antidepressants as adverse effects. Most antidepressants included in the network meta-analysis had relatively higher ORs for insomnia and somnolence compared to placebo. Among them, fluvoxamine, trazodone, and mirtazapine ranked the top three risks for somnolence, whereas reboxetine and vilazodone had the highest risks for insomnia. The relationship curves between the risks of somnolence or insomnia and dose of antidepressants can be linear, inverted U-shape, and other shapes. We hope that these results will help clinicians better take sleep-related adverse effects into consideration and make optimal treatment choices.

## Supplementary Material

zsad177_suppl_Supplementary_Material

## Data Availability

The data underlying this article will be shared on reasonable request to the corresponding author.
