# Correction to: Adverse effects of 21 antidepressants on sleep during acute-phase treatment in major depressive disorder: a systemic review and dose-effect network meta-analys

**DOI:** 10.1093/sleep/zsag042

**Published:** 2026-03-23

This is a correction to: Shuzhe Zhou, Pei Li, Xiaozhen Lv, Xuefeng Lai, Zuoxiang Liu, Junwen Zhou, Fengqi Liu, Yiming Tao, Meng Zhang, Xin Yu, Jingwei Tian, Feng Sun, Adverse effects of 21 antidepressants on sleep during acute-phase treatment in major depressive disorder: a systemic review and dose-effect network meta-analysis, *Sleep*, Volume 46, Issue 10, October 2023, zsad177, https://doi.org/10.1093/sleep/zsad177

The affiliation for co-author Jingwei Tian should read: “^5^School of Pharmacy, Yantai University, Qingquanlu 30#, 264005, Laishan District, Yantai, China” instead of: “^5^School of Pharmacy, Key Laboratory of Molecular Pharmacology and Drug Evaluation (Yantai University), Ministry of Education, Collaborative Innovation Center of Advanced Drug Delivery System and Biotech Drugs in Universities of Shandong, Yantai University, Yantai, China” and in the corresponding author statement this should read: “Jingwei Tian, School of Pharmacy, Yantai University, Qingquanlu 30#, 264005, Laishan District, Yantai, China. Email: tianjingwei618@163.com”. instead of: “Jingwei Tian, Department of Epidemiology and Biostatistics, School of Public Health, Peking University, Qingquanlu 30#, 264005, Laishan District, Yantai, China. Email: tianjingwei618@163.com”.

The errors have been outlined only in this correction notice to preserve the version of record.

