# Supplementary material for: Adverse effects of 21 antidepressants on sleep during acute-phase treatment in major depressive disorder: a systemic review and dose-effect network meta-analysis
Source: Sleep. 2023 Jul 9;46(10):zsad177. doi: 10.1093/sleep/zsad177 (PMC10566234; doi:10.1093/sleep/zsad177)
Supplement: zsad177_suppl_Supplementary_Material [file zsad177_suppl_supplementary_material.docx]

**Adverse effects of 21 antidepressants on sleep during acute-phase treatment in major depressive disorder: A systemic review and dose-effect network meta-analysis**

**Shuzhe Zhou^1,2#^, Pei Li^3#^, Xiaozhen Lv^1,2*^, Xuefeng Lai^3^, Zuoxiang Liu^3^，Junwen Zhou^3^，Fengqi Liu^3^，Yiming Tao^3^，Meng Zhang^3^，Xin Yu^1,2^, Jingwei Tian^5*^, Feng Sun^3,4*^**

^1^Peking University Sixth Hospital, Peking University Institute of Mental Health, Beijing, 100191, China.

^2^NHC Key Laboratory of Mental Health (Peking University), National Clinical Research Center for Mental Disorders (Peking University Sixth Hospital), Beijing, 100191, China.

^3^Department of Epidemiology and Biostatistics, School of Public Health, Peking University, Beijing, 100191, China.

^4^Key Laboratory of Epidemiology of Major Diseases (Peking University), Ministry of Education, Beijing, 100191, China.

^5^School of Pharmacy, Key Laboratory of Molecular Pharmacology and Drug Evaluation (Yantai University), Ministry of Education, Collaborative Innovation Center of Advanced Drug Delivery System and Biotech Drugs in Universities of Shandong, Yantai University, Yantai, 264005, China

***Corresponding authors**

Feng Sun, sunfeng@bjmu.edu.cn;

Full address: Xueyuanlu 38#, 100191, Haidian District, Beijing, China

Xiaozhen Lv, lxz120300@163.com;

Full address: Huayuanbeilu 51#, 100191, Haidian District, Beijing, China

Jingwei Tian, tianjingwei618@163.com;

Full address: Qingquanlu 30#, 264005, Laishan District, Yantai, China

^#^The two authors have contributed equally to this work and share first authorship.

Supplementary files including:

Table S1 Search strategy;

Table S2 Characteristics of included studies;

Table S3 Risk of bias assessment for individual studies;

Table S4 The estimations of meta-regression coefficients of age for somnolence;

Table S5 The estimations of meta-regression coefficients of percentage of females for somnolence;

Table S6 The estimations of meta-regression coefficients of baseline severity for somnolence;

Table S7 The estimations of meta-regression coefficients of age for insomnia；

Table S8 The estimations of meta-regression coefficients of percentage of females for insomnia；

Table S9 The estimations of meta-regression coefficients of baseline severity for insomnia；

Table S10 Local inconsistency detection by node split approach；

Table S11 Secondary outcomes reported by included studies；

Figure S1 SUCRA for somnolence；

Figure S2 SUCRA for insomnia；

Figure S3 Funnel plot of somnolence；

Figure S4 Funnel plot of insomnia；

Figure S5 Illustrations for the similarity of baseline characteristics across comparisons；

Figure S6 Result of sensitivity analyses for somnolence；

Figure S7 Result of sensitivity analyses for insomnia

Table S1 Search strategy

| Database | No | Search terms |
| --- | --- | --- |
| Embase | #1 | ('amitriptyline' OR 'mirtazapine' OR 'duloxetine' OR 'venlafaxine' OR 'paroxetine' OR 'milnacipran' OR 'fluvoxamine' OR 'escitalopram' OR 'nefazodone' OR 'sertraline' OR 'vortioxetine' OR 'agomelatine' OR 'vilazodone' OR 'levomilnacipran' OR 'bupropion' OR 'fluoxetine' OR 'citalopram' OR 'trazodone' OR 'clomipramine' OR 'desvenlafaxine' OR 'reboxetine') AND ('depress' OR 'dysthymi' OR 'dysthymia'/exp OR 'adjustment disorder' OR 'mood disorder' OR 'affective disorder' OR 'affective symptoms') |
| Pubmed | #1 | ("amitriptyline" OR "mirtazapine" OR "duloxetine" OR "venlafaxine" OR "paroxetine" OR "milnacipran" OR "fluvoxamine" OR "escitalopram" OR "nefazodone" OR "sertraline" OR "vortioxetine" OR "agomelatine" OR "vilazodone" OR "levomilnacipran" OR "bupropion" OR "fluoxetine" OR "citalopram" OR "trazodone" OR "clomipramine" OR "desvenlafaxine" OR "reboxetine" ) AND ( "depress*" OR "dysthymia*" OR "dysthymia" OR "adjustment disorder" OR "mood disorder" OR "affective disorder" OR "affective symptoms" ) |
| Web of science-SCI | #1 | ALL=(Amitriptyline) OR ALL=(Mirtazapine) OR ALL=(Duloxetine) OR ALL=(Venlafaxine) OR ALL=(Paroxetine) OR ALL=(Milnacipran) OR ALL=(Fluvoxamine) OR ALL=(Escitalopram) OR ALL=(Nefazodone) OR ALL=(Sertraline) OR ALL=(Vortioxetine) OR ALL=(Agomelatine) OR ALL=(Vilazodone) OR ALL=(Levomilnacipran) OR ALL=(Bupropion) OR ALL=(Fluoxetine) OR ALL=(Citalopram) OR ALL=(Trazodone) OR ALL=(Clomipramine) OR ALL=(Desvenlafaxine) OR ALL=(Reboxetine) |
|  | #2 | ALL=(adjustment disorder*) OR ALL=(mood disorder*) OR ALL=(affective disorder) OR ALL=(affective symptoms) |
|  | #3 | ALL=(depress*) OR ALL=(dysthymi*) |
|  | #4 | #2 AND (#3 OR #4) |
| CENTRAL | #1 | "amitriptyline" OR "mirtazapine" OR "duloxetine" OR "venlafaxine" OR "paroxetine" OR "milnacipran" OR "fluvoxamine" OR "escitalopram" OR "nefazodone" OR "sertraline" OR "vortioxetine" OR "agomelatine" OR "vilazodone" OR "levomilnacipran" OR "bupropion" OR "fluoxetine" OR "citalopram" OR "trazodone" OR "clomipramine" OR "desvenlafaxine" OR "reboxetine" |
|  | #2 | "depress*" OR "dysthymi*" OR "dysthymia" |
|  | #3 | "adjustment disorder*" OR "mood disorder*" OR "affective disorder*" OR "affective symptoms*" |
|  | #4 | #1 AND (#2 OR #3) |
| clinicaltrials.gov | #1 | "Amitriptyline" OR "Mirtazapine" OR "Duloxetine" OR "Venlafaxine" OR "Paroxetine" OR "Milnacipran" OR "Fluvoxamine" OR "Escitalopram" OR "Nefazodone" OR "Sertraline" OR "Vortioxetine" OR "Agomelatine" OR "Vilazodone" OR "Levomilnacipran" \| Completed Studies \| Studies With Results \| depression OR dysthymia OR "adjustment disorder" OR "mood disorder" OR "affective disorder" OR "affective symptoms" |

Table S2 Characteristics of included studies

| Author | Year | Region | Sample size | Female% | Mean age | Drug | Dose(mg/day) | Measurement of baseline severity | Mean | Main outcomes |
| --- | --- | --- | --- | --- | --- | --- | --- | --- | --- | --- |
| Anna^1^ | 2000 | not specified | 176 |  |  | sertraline | 150 | MADRS | 30.3 | ①② |
|  |  |  | 177 |  |  | paroxetine | 40 | MADRS | 30.7 | ①② |
| Aguglia^2^ | 1993 | Europe | 52 | 0.75 | 57.5 | sertraline | 150 | HAMD | 24.8 | ①② |
|  |  |  | 56 | 0.75 | 58.9 | fluoxetine | 60 | HAMD | 25.1 | ①② |
| AK1102365^3^ | 2009 | Asia | 166 | 0.46 | 36.8 | bupropion | 300 | HAMD17 | 19.4 | ① |
|  |  |  | 159 | 0.43 | 36 | placebo | 0 | HAMD17 | 19.8 | ① |
| Alvarez^4^ | 2012 | Europe | 109 | 0.64 | 43.8 | vortioxetine | 5 | MADRS | 34.1 | ② |
|  |  |  | 101 | 0.65 | 42.3 | vortioxetine | 10 | MADRS | 34 | ② |
|  |  |  | 114 | 0.54 | 45 | venlafaxine | 225 | MADRS | 34.2 | ② |
|  |  |  | 105 | 0.66 | 42 | placebo | 0 | MADRS | 33.9 | ② |
| Amini^5^ | 2005 | Asia | 18 | 0.69 | 35 | mirtazapine | 30 | HAMD17 | 25.8 | ① |
|  |  |  | 18 | 0.6 | 37.7 | fluoxetine | 20 | HAMD17 | 24.8 | ① |
| Ansseau^6^ | 1989 | Europe | 44 |  |  | milnacipran | 100 | HAMD17 | 24.4 | ① |
|  |  |  | 45 |  |  | amitriptyline | 150 | HAMD17 | 24.8 | ① |
|  |  |  | 44 |  |  | milnacipran | 200 | HAMD24 | 37.3 | ①② |
|  |  |  | 43 |  |  | amitriptyline | 150 | HAMD24 | 37.6 | ①② |
| Ansseau^7^ | 1991 | Europe | 41 | 0.76 | 46.6 | fluvoxamine | 200 | HAMD24 | 32.69 | ①② |
|  |  |  | 42 | 0.67 | 49.9 | milnacipran | 300 | HAMD24 | 33.94 | ①② |
|  |  |  | 43 | 0.68 | 46.5 | milnacipran | 200 | HAMD24 | 31.89 | ①② |
| Ansseau^8^ | 1994 | Europe | 97 | 0.74 | 44.8 | milnacipran | 100 | HAMD24 | 30.6 | ①② |
|  |  | Europe | 93 | 0.76 | 45 | fluoxetine | 20 | HAMD24 | 32.4 | ①② |
|  |  | Europe | 55 | 0.73 | 45.8 | nefazodone | 400 | MADRS | 34.1 | ② |
|  |  | Europe | 51 | 0.67 | 48.6 | amitriptyline | 200 | MADRS | 35.6 | ② |
| Asnis^9^ | 2013 | North America | 178 | 0.69 | 41.6 | levomilnacipran | 40 | HAMD17 | 24.7 | ② |
|  |  |  | 179 | 0.62 | 41 | levomilnacipran | 80 | HAMD17 | 24.9 | ② |
|  |  |  | 180 | 0.59 | 40.3 | levomilnacipran | 120 | HAMD17 | 25 | ② |
|  |  |  | 176 | 0.61 | 41.3 | placebo | 0 | HAMD17 | 24.6 | ② |
| Bakish^10^ | 1992 | North America | 58 |  | 42 | amitriptyline | 150 | HAMD17 | 22.81 | ② |
|  |  |  | 56 |  | 44 | placebo | 0 | HAMD17 | 23.04 | ② |
| Baldwin^11^ | 2006 | Europe | 159 | 0.75 | 45.1 | paroxetine | 40 | MADRS | 29.7 | ①② |
|  |  |  | 166 | 0.74 | 44.9 | escitalopram | 20 | MADRS | 29.6 | ①② |
| Baldwin^12^ | 2012 | Cross-continental | 155 | 0.71 | 46 | vortioxetine | 2.5 | MADRS | 31.6 | ①② |
|  |  |  | 159 | 0.66 | 44.7 | vortioxetine | 5 | MADRS | 32.7 | ①② |
|  |  |  | 153 | 0.68 | 45.2 | vortioxetine | 10 | MADRS | 31.8 | ①② |
|  |  |  | 157 | 0.68 | 45.3 | duloxetine | 60 | MADRS | 31.4 | ①② |
|  |  |  | 152 | 0.7 | 43.4 | placebo | 0 | MADRS | 31.7 | ①② |
| Ban^13^ | 1998 | Cross-continental | 84 | 0.5 | 47.6 | reboxetine | 8 | MADRS | 17.28 | ② |
|  |  |  | 89 | 0.64 | 46.7 | placebo | 0 | MADRS | 16.4 | ② |
| Battegay^14^ | 1985 | Europe | 11 | 0.55 | 41.5 | paroxetine | 10 |  |  | ①② |
|  |  |  | 10 | 0.6 | 36.9 | amitriptyline | 50 |  |  | ①② |
| Behnke^15^ | 2003 | Cross-continental | 176 | 0.56 | 42 | mirtazapine | 45 |  |  | ①② |
|  |  |  | 170 | 0.62 | 41 | sertraline | 150 |  |  | ①② |
| Berlanga^16^ | 1997 | South America | 37 | 0.76 | 39.9 | fluoxetine | 40 | HAMD17 | 23.7 | ①② |
|  |  |  | 37 | 0.78 | 41.5 | nefazodone | 500 | HAMD17 | 25.1 | ①② |
| Bignamini^17^ | 1992 | Europe | 156 |  |  | paroxetine | 30 | HAMD | 30.5 | ① |
|  |  |  | 153 |  |  | amitriptyline | 150 | HAMD | 30.9 | ① |
| Blacker^18^ | 1988 | Europe | 112 |  | 45 | trazodone | 150 | HAMD21 | 26.3 | ① |
|  |  |  | 44 |  | 42 | amitriptyline | 100 | HAMD21 | 26.7 | ① |
| Andreoli^19^ | 2002 | Cross-continental | 126 | 0.67 | 40 | reboxetine | 10 | HAMD21 | 26.8 | ② |
|  |  |  | 127 | 0.65 | 40.2 | fluoxetine | 40 | HAMD21 | 26.9 | ② |
| Bose^20^ | 2008 | North America | 130 | 0.59 | 68.1 | escitalopram | 20 | MADRS | 29.4 | ② |
|  |  |  | 134 | 0.6 | 68.5 | placebo | 0 | MADRS | 28.4 | ② |
| Bougerol^21^ | 1997 | Cross-continental | 158 |  | 43 | citalopram | 40 | MADRS | 31.2 | ①② |
|  |  |  | 158 |  | 41 | fluoxetine | 20 | MADRS | 31.8 | ①② |
| Study 049^22^ | 1998 | North America | 107 | 0.55 | 39.9 | reboxetine | 10 | MADRS | 29.2 | ①② |
|  |  |  | 105 | 0.58 | 39.7 | placebo | 0 | MADRS | 29.2 | ①② |
| Boyer^23^ | 2008 | Cross-continental | 166 | 0.7 | 44 | desvenlafaxine | 50 | HAMD17 | 24 | ② |
|  |  |  | 158 | 0.71 | 46 | desvenlafaxine | 100 | HAMD17 | 24 | ② |
|  |  |  | 161 | 0.68 | 46 | placebo | 0 | HAMD17 | 24 | ② |
| Brunoni^24^ | 2012 | South America | 30 |  | 41 | sertraline | 50 | MADRS | 30.5 | ② |
|  |  |  | 30 |  | 46.4 | placebo | 0 | MADRS | 30.76 | ② |
| Byerley^25^ | 1988 | North America | 32 | 0.75 | 38.9 | fluoxetine | 80 | HAMD21 | 27.1 | ② |
|  |  |  | 29 | 0.09 | 37.5 | placebo | 0 | HAMD21 | 27.4 | ② |
| CAGO178A2303^26^ | 2008 | North America | 169 | 0.62 | 42.1 | agomelatine | 50 | HAMD17 | 27.2 | ① |
|  |  |  | 168 | 0.59 | 43.7 | paroxetine | 40 | HAMD17 | 27 | ① |
|  |  |  | 166 | 0.67 | 42.9 | placebo | 0 | HAMD17 | 26.9 | ① |
| Chouinard^27^ | 1985 | North America | 25 | 0.7 | 41 | fluoxetine | 80 | HAMD21 | 27.6 | ① |
|  |  |  | 28 | 0.71 | 39 | amitriptyline | 300 | HAMD21 | 25.9 | ① |
| Chouinard^28^ | 1998 | North America | 102 | 0.64 | 40.6 | paroxetine | 50 | HAMD21 | 25.91 | ①② |
|  |  |  | 101 | 0.59 | 41.2 | fluoxetine | 80 | HAMD21 | 25.45 | ①② |
| CL3-20098-022^29^ | 2001 | Europe | 133 | 0.67 | 41 | agomelatine | 25 | HAMD17 | 27.6 | ①② |
|  |  |  | 137 | 0.67 | 42.9 | fluoxetine | 20 | HAMD17 | 27.5 | ①② |
|  |  |  | 149 | 0.67 | 43 | placebo | 0 | HAMD17 | 28 | ①② |
| CL3-20098-023^30^ | 2001 | Europe | 142 | 0.75 | 40.6 | agomelatine | 25 | HAMD17 | 25.7 | ①② |
|  |  |  | 138 | 0.75 | 40.9 | paroxetine | 20 | HAMD17 | 26.1 | ①② |
|  |  |  | 137 | 0.75 | 41.2 | placebo | 0 | HAMD17 | 26 | ①② |
| CL3-20098-026^31^ | 2001 | Europe | 109 |  | 66.8 | agomelatine | 25 | HAMD17 | 23.4 | ①② |
|  |  |  | 109 |  | 67.8 | placebo | 0 | HAMD17 | 23 | ①② |
| CL3-20098-062^32^ | 2010 | Europe | 202 | 0.72 | 42.8 | agomelatine | 50 | HAMD17 | 26.2 | ① |
|  |  |  | 216 | 0.72 | 42.8 | duloxetine | 60 | HAMD17 | 26.3 | ① |
| CL3-20098-070^33^ | 2011 | Cross-continental | 151 |  | 71.9 | agomelatine | 50 | HAMD17 | 26.8 | ① |
|  |  |  | 71 |  | 71.7 | placebo | 0 | HAMD17 | 26.7 | ① |
| Claghorn^34^ | 1983 | North America | 85 | 0.57 | 39 | amitriptyline | 300 | HAMD21 | 26.51 | ①② |
|  |  |  | 87 | 0.57 | 39.1 | placebo | 0 | HAMD21 | 27.11 | ①② |
| Claghorn^35^ | 1995 | North America | 45 | 0.52 | 39 | mirtazapine | 35 | MADRS | 27.9 | ① |
|  |  |  | 45 | 0.45 | 40 | placebo | 0 | MADRS | 29.7 | ① |
| Claghorn^36^ | 1996 | North America | 50 |  | 39 | fluvoxamine | 150 | HAMD21 | 26.09 | ①② |
|  |  |  | 50 |  | 39 | placebo | 0 | HAMD21 | 26.42 | ①② |
| Clerc^37^ | 1994 | Europe | 34 |  | 53.6 | fluoxetine | 40 | MADRS | 35.7 | ② |
|  |  |  | 34 |  | 49 | venlafaxine | 200 | MADRS | 34.8 | ② |
| Clerc^38^ | 2001 | Europe | 57 | 0.75 | 48.7 | milnacipran | 100 | MADRS | 37.1 | ①② |
|  |  |  | 56 | 0.82 | 51.2 | fluvoxamine | 200 | MADRS | 35.5 | ①② |
| Cohn^39^ | 1985 | Europe | 30 | 0.72 | 54 | fluoxetine | 80 | HAMD | 25.75 | ①② |
|  |  |  | 29 | 0.48 | 58 | placebo | 0 | HAMD | 25.14 | ①② |
| Coleman^40^ | 1999 | North America | 118 | 0.54 | 38.3 | sertraline | 200 | HAMD31 | 34.8 | ② |
|  |  |  | 122 | 0.56 | 38.1 | bupropion | 400 | HAMD31 | 34.5 | ② |
|  |  |  | 124 | 0.59 | 38.5 | placebo | 0 | HAMD31 | 34 | ② |
| Coleman^41^ | 2001 | North America | 150 | 0.63 | 36.6 | bupropion | 400 | HAMD21 | 24.6 | ①② |
|  |  |  | 154 | 0.66 | 37.1 | fluoxetine | 60 | HAMD21 | 24.5 | ①② |
|  |  |  | 152 | 0.61 | 36.7 | placebo | 0 | HAMD21 | 24.4 | ①② |
| Corrigan^42^ | 2000 | North America | 35 |  | 42 | fluoxetine | 20 | MADRS | 28.8 | ①② |
|  |  |  | 35 |  | 42 | placebo | 0 | MADRS | 26.8 | ①② |
| Corruble^43^ | 2013 | Cross-continental | 160 | 0.69 | 42.8 | escitalopram | 20 | HAMD17 | 26.6 | ①② |
|  |  |  | 164 | 0.73 | 43.6 | agomelatine | 50 | HAMD17 | 26.8 | ①② |
| Croft^44^ | 1999 | not specified | 121 | 0.5 | 37.4 | placebo | 0 | HAMD29 | 32.2 | ①② |
|  |  |  | 119 | 0.5 | 36 | sertraline | 200 | HAMD29 | 33.1 | ①② |
|  |  |  | 120 | 0.51 | 35.9 | bupropion | 400 | HAMD29 | 32.9 | ①② |
| Croft^45^ | 2014 | North America | 258 | 0.56 | 41.1 | placebo | 0 | MADRS | 30.9 | ② |
|  |  |  | 260 | 0.51 | 39.3 | vilazodone | 40 | MADRS | 30.6 | ② |
| Cunningham^46^ | 1994 | not specified | 76 | 0.67 | 76 | placebo | 0 |  | 28 | ① |
|  |  |  | 77 | 0.69 | 77 | trazodone | 400 |  | 28.26 | ① |
|  |  |  | 76 | 0.63 | 76 | venlafaxine | 200 | MADRS | 27.4 | ① |
| Cunningham^47^ | 1997 | not specified | 100 | 0.41 | 39.9 | placebo | 0 |  | 26.6 | ① |
|  |  |  | 203 | 0.35 | 40.9 | venlafaxine | 75 | MADRS | 26.5 | ① |
| Blier^48^ | 2009 | North America | 19 | 0.48 | 40 | paroxetine | 30 | MADRS | 32.3 | ①② |
|  |  |  | 21 | 0.25 | 46 | mirtazapine | 45 | MADRS | 32 | ①② |
| DeMartinis^49^ | 2007 | North America | 120 |  | 40 | placebo | 0 |  | 23.1 | ①② |
|  |  |  | 116 |  | 39 | desvenlafaxine | 400 |  | 23 | ①② |
|  |  |  | 116 |  | 40.7 | desvenlafaxine | 200 |  | 22.9 | ①② |
|  |  |  | 118 |  | 40.4 | desvenlafaxine | 100 | HAMD17 | 23.2 | ①② |
| Ronchi^50^ | 1998 | not specified | 32 |  | 68.6 | amitriptyline | 100 | MADRS |  | ①② |
|  |  |  | 33 |  | 69.1 | fluoxetine | 20 | MADRS |  | ①② |
| Detke^51^ | 2002 | North America | 139 | 0.71 | 41 | placebo | 0 | HAMD17 | 20.46 | ①② |
|  |  |  | 267 | 0.66 | 41 | duloxetine | 60 | HAMD17 | 20.33 | ①② |
| Detke^52^ | 2004 | not specified | 93 | 0.74 | 43.7 | placebo | 0 | MADRS | 21.5 | ①② |
|  |  |  | 93 | 0.75 | 44.7 | duloxetine | 120 | MADRS | 22.1 | ①② |
|  |  |  | 94 | 0.74 | 43.1 | duloxetine | 80 | MADRS | 21.8 | ①② |
|  |  |  | 367 | 0.67 | 42 | paroxetine | 20 | MADRS | 22.3 | ①② |
| Dierick^53^ | 1996 | not specified | 161 | 0.64 | 43.2 | fluoxetine | 20 | MADRS | 30.4 | ② |
|  |  |  | 153 | 0.65 | 43.7 | venlafaxine | 150 | HAMD | 27.3 | ② |
| Dimidjian^54^ | 2006 | North America | 100 | 0.68 | 39.9 | paroxetine | 50 | HAMD | 20.87 | ② |
|  |  |  | 53 | 0.72 | 39.9 | placebo | 0 | HAMD | 21.15 | ② |
| Doogan^55^ | 1994 | not specified | 99 | 0.71 | 46 | sertraline | 100 | MADRS | 27.9 | ① |
|  |  |  | 101 | 0.64 | 45.4 | placebo | 0 | MADRS | 27.3 | ① |
| Dube^56^ | 2010 | Cross-continental | 62 | 0.46 | 34.3 | escitalopram | 20 | HAMD21 |  | ② |
|  |  |  | 138 | 0.46 | 37.5 | placebo | 0 | HAMD21 |  | ② |
| Dunbar^57^ | 1993 | North America | 170 | 0.51 | 40 | paroxetine | 50 | MADRS | 28.2 | ①② |
|  |  |  | 171 | 0.52 | 42 | placebo | 0 | MADRS | 28.5 | ①② |
| Fabre^58^ | 1995 | North America | 95 | 0.51 | 37.3 | sertraline | 50 | HAMD | 24.8 | ①② |
|  |  |  | 92 | 0.57 | 37.1 | sertraline | 100 | HAMD | 24.9 | ①② |
|  |  |  | 91 | 0.63 | 38.6 | sertraline | 200 | HAMD | 25.7 | ①② |
|  |  |  | 91 | 0.44 | 37.3 | placebo | 0 | HAMD | 25.3 | ①② |
| Fabre^59^ | 1996 | North America | 50 |  | 45 | fluvoxamine | 120 | HAMD | 27.7 | ①② |
|  |  |  | 50 |  | 41 | placebo | 0 | HAMD | 26 | ①② |
| Falk^60^ | 1989 | North America | 13 | 0.77 | 67.5 | trazodone | 250 | HAMD | 26.17 | ② |
|  |  |  | 14 | 0.71 | 69.1 | fluoxetine | 60 | HAMD | 23.77 | ② |
| Fava^61^ | 2002 | North America | 96 | 0.55 | 44 | sertraline | 200 | HAMD | 21 | ①② |
|  |  |  | 92 | 0.58 | 42.1 | fluoxetine | 60 | HAMD | 20.5 | ①② |
|  |  |  | 96 | 0.56 | 42.5 | paroxetine | 60 | HAMD | 20.6 | ①② |
| Fava^62^ | 2005 | North America | 43 | 0.53 | 36.7 | placebo | 0 | HAMD | 19.9 | ①② |
|  |  |  | 47 | 0.65 | 37.8 | fluoxetine | 20 | HAMD17 | 19.6 | ①② |
| Feighner^63^ | 1989 | North America | 59 | 0.79 | 39.7 | placebo | 0 | HAMD21 | 25.9 | ① |
|  |  |  | 62 | 0.69 | 45 | fluoxetine | 80 | HAMD21 | 25.6 | ① |
|  |  |  | 31 | 0.77 | 41 | fluvoxamine | 280 | HAMDunspecified | 25 | ①② |
|  |  |  | 19 | 0.89 | 41 | placebo | 0 | HAMDunspecified | 25 | ①② |
| Dunbar^64^ | 1991 | North America | 241 | 0.52 | 40.3 | paroxetine | 50 | HRSD | 26.5 | ① |
|  |  |  | 244 | 0.48 | 40.1 | placebo | 0 | HRSD | 26.6 | ① |
| Gentil^65^ | 2000 | South America | 57 | 0.8 | 37.9 | venlafaxine | 150 | HAMD | 23.9 | ①② |
|  |  |  | 59 | 0.81 | 39.1 | amitriptyline | 150 | HAMD | 24.5 | ①② |
| Geretsegger^66^ | 1995 | Europe | 44 |  | 71 | paroxetine | 30 | HAMD21 | 26.8 | ② |
|  |  |  | 47 |  | 71.3 | amitriptyline | 150 | HAMD21 | 28.3 | ② |
| Goldstein^67^ | 2004 | North America | 91 | 0.68 | 43.36 | duloxetine | 40 | HAMD17 | 17.47 | ①② |
|  |  |  | 84 | 0.61 | 43.68 | duloxetine | 80 | HAMD17 | 17.44 | ①② |
|  |  |  | 90 | 0.66 | 43.18 | placebo | 0 | HAMD17 | 17.79 | ①② |
|  |  |  | 89 | 0.52 | 44.43 | paroxetine | 20 | HAMD17 | 17.97 | ①② |
|  |  |  | 86 | 0.56 | 41 | duloxetine | 40 | HAMD17 | 18.74 | ①② |
| Goldstein^67^ | 2004 | North America | 91 | 0.62 | 41 | duloxetine | 80 | HAMD17 | 17.86 | ①② |
|  |  |  | 89 | 0.64 | 40 | placebo | 0 | HAMD17 | 17.2 | ①② |
|  |  |  | 87 | 0.64 | 40 | paroxetine | 20 | HAMD17 | 17.83 | ①② |
| Gommoll^68^ | 2014 | North America | 182 | 0.64 | 43.7 | placebo | 0 | HAMD17 | 24.4 | ② |
|  |  |  | 175 | 0.57 | 42.8 | levomilnacipran | 120 | HAMD17 | 24.9 | ② |
| Goodarzi^69^ | 2015 | Asia | 24 | 0.43 | 38 | mirtazapine | 30 | HAMD | 31 | ① |
|  |  |  | 28 | 0.57 | 40.35 | citalopram | 40 | HAMD | 29 | ① |
| Griebel^70^ | 2012 | North America | 84 | 0.62 | 40.8 | escitalopram | 10 | HAMD17 | 23.4 | ①② |
|  |  |  | 76 | 0.63 | 41.2 | placebo | 0 | HAMD17 | 23 | ①② |
| Griebel^70^ | 2012 | Cross-continental | 80 | 0.7 | 40 | paroxetine | 20 | HAMD17 | 24.9 | ①② |
|  |  |  | 79 | 0.73 | 40.1 | placebo | 0 | HAMD17 | 24.8 | ①② |
| Guelfi^71^ | 1998 | Europe | 100 | 0.6 | 45.6 | milnacipran | 100 | HAMD17 | 27.8 | ② |
|  |  |  | 100 | 0.66 | 45.2 | milnacipran | 200 | HAMD17 | 27.7 | ② |
|  |  |  | 100 | 0.75 | 45.8 | fluoxetine | 20 | HAMD17 | 27.4 | ② |
| Hale^72^ | 2010 | Cross-continental | 252 | 0.77 | 41.8 | agomelatine | 50 | HAMD17 | 28.5 | ① |
|  |  |  | 263 | 0.78 | 42.7 | fluoxetine | 40 | HAMD16 | 28.7 | ① |
| Halikas^73^ | 1995 | North America | 50 | 0.6 | 61 | trazodone | 280 | HAMD | 24.6 | ① |
|  |  |  | 50 | 0.59 | 62 | placebo | 0 | HAMD | 23.5 | ① |
| Hewett^74^ | 2008 | Cross-continental | 188 | 0.74 | 41.8 | bupropion | 300 | MADRS | 30.4 | ② |
|  |  |  | 189 | 0.68 | 42.7 | venlafaxine | 150 | MADRS | 30 | ② |
|  |  |  | 199 | 0.72 | 41.8 | placebo | 0 | MADRS | 30.4 | ② |
| Hewett^75^ | 2010 | Cross-continental | 203 | 0.63 | 45.6 | bupropion | 300 | MADRS | 30.6 | ①② |
|  |  |  | 187 | 0.67 | 44.5 | placebo | 0 | MADRS | 30.6 | ①② |
|  |  |  | 198 | 0.68 | 44.1 | venlafaxine | 150 | MADRS | 30.1 | ①② |
| Hicks^76^ | 2002 | Europe | 20 | 0.6 | 42.75 | nefazodone | 600 | HRSD |  | ① |
|  |  |  | 20 | 0.55 | 42.95 | paroxetine | 40 | HRSD |  | ① |
| Higuchi^77^ | 2016 | Asia | 174 | 0.52 | 38.4 | venlafaxine | 75 | HAMD17 | 22.4 | ① |
|  |  |  | 180 | 0.48 | 38.3 | venlafaxine | 225 | HAMD17 | 22.4 | ① |
|  |  |  | 184 | 0.51 | 38.6 | placebo | 0 | HAMD17 | 22.6 | ① |
| Higuchi^78^ | 2011 | Asia | 83 | 0.58 | 35.5 | Paroxetine | 40 | HAMD17 | 22.7 | ① |
|  |  |  | 161 | 0.53 | 36.4 | Paroxetine | 50 | HAMD17 | 22.7 | ① |
|  |  |  | 172 | 0.55 | 36.8 | placebo | 0 | HAMD17 | 22.6 | ① |
| Hormazabal^79^ | 1985 | South America | 20 | 0.85 | 43.9 | amitriptyline | 150 | HAMD21 | 36.7 | ① |
|  |  |  | 20 | 0.8 | 42.3 | placebo | 0 | HAMD21 | 35.8 | ① |
| Hoyberg^80^ | 1996 | Europe | 56 | 0.44 | 70 | mirtazapine | 45 | HRSD | 26.7 | ① |
|  |  |  | 59 | 0.51 | 71 | amitriptyline | 90 | HRSD | 25.7 | ① |
| Hsu^81^ | 2011 | Asia | 25 | 0.52 | 43.4 | citalopram | 20 | MADRS | 36.6 | ①② |
|  |  |  | 26 | 0.67 | 38 | sertraline | 50 | MADRS | 38.2 | ①② |
| Itil^82^ | 1983 | North America | 22 | 0.43 | 41.3 | fluvoxamine | 50 | HAMD (16 items) | 20.3 | ① |
|  |  |  | 22 | 0.43 | 41.3 | placebo | 0 | HAMD (16 items) | 19.7 | ① |
| Iwata^83^ | 2013 | Cross-continental | 237 | 0.56 | 39 | desvenlafaxine | 50 | HAMD17 | 23 | ① |
|  |  |  | 235 | 0.55 | 40 | placebo | 0 | HAMD17 | 23 | ① |
|  |  |  | 237 | 0.55 | 40 | desvenlafaxine | 25 | HAMD17 | 23 | ① |
| Judd^84^ | 1993 | Oceania | 28 | 0.66 | 41.7 | amitriptyline | 200 | Hamilton total score | 23.7 | ② |
|  |  |  | 30 | 0.66 | 41.7 | fluoxetine | 20 | Hamilton total score | 24.4 | ② |
| Kamijima^85^ | 2013 | Asia | 303 | 0.53 | 35.9 | milnacipran | 100 | HAMD | 22.1 | ① |
|  |  |  | 301 | 0.51 | 36.7 | milnacipran | 200 | HAMD | 22.3 | ① |
|  |  |  | 301 | 0.55 | 36.6 | paroxetine | 40 | HAMD | 22.1 | ① |
| Kasper^86^ | 2005 | Europe | 55 | 0.58 | 43.5 | trazodone | 450 | HAMD | 21 | ①② |
|  |  |  | 53 | 0.68 | 44.3 | paroxetine | 40 | HAMD | 20.9 | ①② |
|  |  |  | 174 | 0.75 | 75 | escitalopram | 10 | MADS | 28.2 | ①② |
|  |  |  | 164 | 0.77 | 75 | fluoxetine | 20 | MADS | 28.5 | ①② |
|  |  |  | 180 | 0.76 | 75 | placebo | 0 | MADS | 28.6 | ①② |
| Kasper^87^ | 2012 | Europe | 419 | 0.68 | 41.9 | escitalopram | 20 | MADRS | 35.4 | ①② |
|  |  |  | 71 | 0.75 | 42.6 | placebo | 0 | MADRS | 34.7 | ①② |
| Katona^88^ | 2012 | Cross-continental | 145 | 0.62 | 70.3 | placebo | 0 | HAMD24 | 29.4 | ① |
|  |  |  | 151 | 0.66 | 70.9 | duloxetine | 60 | HAMD24 | 28.5 | ① |
| Keegan^89^ | 1991 | North America | 20 | 0.67 | 39.5 | fluoxetine | 40 | HAMD | 27.3 | ② |
|  |  |  | 22 | 0.67 | 47.8 | amitriptyline | 150 | HAMD | 33.28 | ② |
| Keller^90^ | 2006 | North America | 297 | 0.68 | 39 | paroxetine | 20 | HAMD17 | 27.5 | ①② |
|  |  |  | 155 | 0.61 | 41.4 | placebo | 0 | HAMD17 | 27.7 | ①② |
| Keller^90^ | 2006 | Cross-continental | 164 | 0.64 | 40.6 | paroxetine | 20 | HAMD17 | 28.3 | ①② |
|  |  |  | 161 | 0.71 | 41.3 | placebo | 0 | HAMD17 | 28.3 | ①② |
| Keller^91^ | 2007 | North America | 275 | 0.61 | 40 | fluoxetine | 60 | HAMD17 | 23 | ①② |
|  |  |  | 821 | 0.65 | 39.6 | venlafaxine | 300 | HAMD17 | 22.6 | ①② |
| Kennedy^92^ | 2014 | Cross-continental | 138 | 0.65 | 46.5 | agomelatine | 25 | HAMD17 | 26.7 | ① |
|  |  |  | 137 | 0.72 | 43.1 | agomelatine | 50 | HAMD17 | 26.7 | ① |
|  |  |  | 141 | 0.77 | 45 | placebo | 0 | HAMD17 | 26.6 | ① |
|  |  |  | 133 | 0.79 | 45.4 | agomelatine | 10 | HAMD17 | 27.3 | ① |
| Khan^93^ | 1998 | North America | 85 | 0.68 | 43.3 | venlafaxine | 75 | HAMD21 | 24.3 | ①② |
|  |  |  | 90 | 0.64 | 40 | venlafaxine | 150 | HAMD21 | 24.5 | ①② |
|  |  |  | 83 | 0.6 | 43.6 | venlafaxine | 200 | HAMD21 | 24.8 | ①② |
|  |  |  | 95 | 0.61 | 40.2 | placebo | 0 | HAMD21 | 25.1 | ①② |
| Koshino^94^ | 2013 | Asia | 192 | 0.56 | 37.5 | bupropion | 300 | MADRS | 32.1 | ② |
|  |  |  | 187 | 0.54 | 37.9 | placebo | 0 | MADRS | 31.9 | ② |
|  |  |  | 190 | 0.52 | 36 | bupropion | 150 | MADRS | 32.1 | ② |
| Kramer^95^ | 1998 | North America | 72 |  |  | paroxetine | 20 | HAMD21 |  | ①② |
|  |  |  | 70 |  |  | placebo | 0 | HAMD21 |  | ①② |
| Kyle^96^ | 1998 | Europe | 179 | 0.73 | 73.4 | citalopram | 40 | MADRS | 27.8 | ① |
|  |  |  | 186 | 0.74 | 74.1 | amitriptyline | 100 | MADRS | 29.4 | ① |
| Lalit^97^ | 2004 | Asia | 69 | 0.57 | 33 | escitalopram | 20 | HAMD | 26 | ② |
|  |  |  | 74 | 0.58 | 33 | citalopram | 40 | HAMD | 25 | ② |
|  |  |  | 71 | 0.49 | 37 | sertraline | 150 | HAMD | 25 | ② |
| Learned^98^ | 2012 | Cross-continental | 133 | 0.39 | 43 | venlafaxine | 225 | HAMD17 |  | ①② |
|  |  |  | 126 | 0.37 | 41.9 | placebo | 0 | HAMD17 |  | ①② |
|  |  |  | 166 | 0.33 | 44.4 | paroxetine | 30 | MADRS | 30.3 | ② |
|  |  |  | 156 | 0.25 | 41.8 | placebo | 0 | MADRS | 31.8 | ② |
| Lecrubier^99^ | 1997 | Europe | 78 | 0.71 | 38.3 | Venlafaxine | 125 | MADRS | 24.9 | ①② |
|  |  |  | 76 | 0.63 | 40.5 | Placebo | 0 | MADRS | 24.2 | ①② |
| Lee^100^ | 2007 | Cross-continental | 238 | 0.66 | 39 | duloxetine | 60 | HAMD17 | 21.1 | ① |
|  |  |  | 240 | 0.74 | 38 | paroxetine | 20 | HAMD17 | 21.1 | ① |
| Lemoine^101^ | 2007 | Europe | 165 | 0.75 | 40.7 | agomelatine | 25 | HAMD17 | 25.9 | ①② |
|  |  |  | 167 | 0.67 | 39.6 | venlafaxine | 75 | HAMD17 | 26 | ①② |
| Lepine^102^ | 2000 | Europe | 82 | 0.71 | 42.3 | sertraline | 200 | HAMD17 | 29.8 | ② |
|  |  |  | 84 | 0.69 | 41.8 | clomipramine | 200 | HAMD17 | 29.6 | ② |
| Lepola^103^ | 2003 | Europe | 156 | 0.75 | 43 | escitalopram | 20 | MADRS | 29 | ①② |
|  |  |  | 161 | 0.69 | 44 | citalopram | 40 | MADRS | 29.2 | ①② |
|  |  |  | 154 | 0.72 | 43 | placebo | 0 | MADRS | 28.7 | ①② |
| Lieberman^104^ | 2008 | Europe | 245 | 0.65 | 42 | placebo | 0 | HAMD17 |  | ① |
|  |  |  | 127 | 0.72 | 46 | venlafaxine | 150 | HAMD17 | 25.8 | ① |
|  |  |  | 127 | 0.7 | 46 | venlafaxine | 225 | HAMD17 | 25.1 | ① |
| Liebowitz^105^ | 2008 | North America | 151 | 0.63 | 42 | desvenlafaxine | 50 | HAMD17 | 23.37 | ② |
|  |  |  | 148 | 0.62 | 43 | desvenlafaxine | 100 | HAMD17 | 23.35 | ② |
|  |  |  | 152 | 0.53 | 43 | placebo | 0 | HAMD17 | 23.02 | ② |
| Liebowitz^106^ | 2013 | North America | 227 | 0.62 | 42 | placebo | 0 | HAMD17 | 23 | ② |
|  |  |  | 228 | 0.6 | 41 | Desvenlafaxine | 10 | HAMD17 | 23 | ② |
|  |  |  | 227 | 0.6 | 43 | Desvenlafaxine | 50 | HAMD17 | 23 | ② |
| Loo^107^ | 2002 | Europe | 141 | 0.67 | 42.3 | agomelatine | 1 | HAMD17 | 27.9 | ①② |
|  |  |  | 147 | 0.67 | 42.3 | agomelatine | 5 | HAMD17 | 27.3 | ①② |
|  |  |  | 137 | 0.67 | 42.3 | agomelatine | 25 | HAMD17 | 27.4 | ①② |
|  |  |  | 139 | 0.67 | 42.3 | placebo | 0 | HAMD17 | 27.4 | ①② |
|  |  |  | 147 | 0.67 | 42.3 | paroxetine | 20 | HAMD17 | 27.3 | ①② |
| Lydiard^108^ | 1997 | North America | 131 | 0.69 | 39 | amitriptyline | 150 | HAMD17 | 22.1 | ①② |
|  |  |  | 129 | 0.67 | 40.2 | placebo | 0 | HAMD17 | 22.1 | ①② |
|  |  |  | 132 | 0.64 | 41.2 | sertraline | 200 | HAMD17 | 21.5 | ①② |
| M/2020/0046 (Study 046)^22^ | - | North America | 265 | 0.71 | 39.9 | reboxetine | 10 | HAMD21 | 23 | ①② |
|  |  |  | 257 | 0.7 | 39 | placebo | 0 | HAMD21 | 23 | ①② |
|  |  |  | 265 | 0.69 | 39.8 | paroxetine | 40 | HAMD21 | 22.8 | ①② |
| M/2020/0047 (Study 047)^109^ | unpublished | North America | 258 | 0.74 | 39.3 | reboxetine | 10 | HAMD21 | 24.2 | ①② |
|  |  |  | 254 | 0.82 | 37.1 | placebo | 0 | HAMD21 | 23.7 | ①② |
|  |  |  | 262 | 0.72 | 39.8 | paroxetine | 40 | HAMD21 | 23.9 | ①② |
| Mahableshwarkar^110^ | 2013 | North America | 153 | 0.61 | 42.6 | placebo | 0 | HAMD24 | 29.5 | ①② |
|  |  |  | 153 | 0.64 | 42.6 | vortioxetine | 2.5 | HAMD24 | 29.8 | ①② |
|  |  |  | 153 | 0.69 | 43.1 | vortioxetine | 5 | HAMD24 | 29.8 | ①② |
|  |  |  | 152 | 0.6 | 42.7 | duloxetine | 60 | HAMD24 | 28.7 | ①② |
| Mahableshwarkar^111^ | 2015 | North America | 161 | 0.72 | 42.4 | placebo | 0 | MADRS | 31.6 | ② |
|  |  |  | 147 | 0.71 | 43.1 | vortioxetine | 15 | MADRS | 31.9 | ② |
|  |  |  | 154 | 0.74 | 42.8 | vortioxetine | 20 | MADRS | 32 | ② |
|  |  |  | 152 | 0.78 | 43.4 | duloxetine | 60 | MADRS | 32.9 | ② |
| Mao^112^ | 2008 | Asia | 117 | 0.62 | 40.7 | fluoxetine | 20 | HAMD17 | 24.1 | ① |
|  |  |  | 123 | 0.47 | 37.1 | escitalopram | 10 | HAMD17 | 24.7 | ① |
| Mao^113^ | 2015 | North America | 19 | 0.53 | 41.4 | sertraline | 50 | HAMD17 | 15.4 | ② |
|  |  |  | 18 | 0.44 | 46.7 | placebo | 0 | HAMD17 | 14.4 | ② |
| Marchesi^114^ | 1998 | Europe | 67 | 0.73 | 43.6 | fluoxetine | 20 | HAMD17 | 25.5 | ① |
|  |  |  | 75 | 0.75 | 43.6 | amitriptyline | 225 | HAMD17 | 25.3 | ① |
| Mathews^115^ | 2015 | North America | 292 | 0.58 | 41.7 | vilazodone | 20 | MADRS | 31 | ①② |
|  |  |  | 291 | 0.57 | 40.8 | vilazodone | 40 | MADRS | 30.8 | ①② |
|  |  |  | 290 | 0.56 | 42 | placebo | 0 | MADRS | 31.3 | ①② |
|  |  |  | 289 | 0.59 | 42.6 | citalopram | 40 | MADRS | 31.1 | ①② |
| McGrath^116^ | 2000 | South America | 49 | 0.64 | 41.6 | fluoxetine | 20 | HAMD17 |  | ① |
|  |  |  | 52 | 0.64 | 41.6 | placebo | 0 | HAMD17 |  | ① |
| McPartlin^117^ | 1998 | Europe | 178 | 0.69 | 44 | paroxetine | 20 | HAMD17 | 23 | ①② |
|  |  |  | 183 | 0.68 | 45 | venlafaxine | 75 | HAMD17 | 23 | ①② |
| Moller^118^ | 1993 | Cross-continental | 78 | 0.71 | 47.5 | paroxetine | 50 | HAMD21 | 28.6 | ①② |
|  |  |  | 75 | 0.71 | 46.6 | amitriptyline | 250 | HAMD21 | 28.9 | ①② |
| Moller^119^ | 2000 | Europe | 116 | 0.63 | 47.7 | sertraline | 100 | HAMD21 | 27.1 | ① |
|  |  |  | 124 | 0.7 | 48.1 | amitriptyline | 150 | HAMD21 | 27.5 | ① |
| Montgomery^120^ |  | not specified | 64 |  |  | citalopram | 40 | HAMD17 | 22.98 | ① |
|  |  |  | 66 |  |  | placebo | 0 | HAMD17 | 23.72 | ① |
| Montgomery^121^ | 2004 | not specified | 148 | 0.73 | 49 | escitalopram | 20 | HAMD17 | 19.99 | ①② |
|  |  |  | 145 | 0.71 | 47 | venlafaxine | 150 | HAMD17 | 20.44 | ①② |
| Moon^122^ | 1994 | Europe | 51 | 0.43 | 42 | sertraline | 150 | HAMD17 | 23.9 | ①② |
|  |  |  | 55 | 0.6 | 45 | clomipramine | 150 | HAMD17 | 22.7 | ①② |
| Moore^123^ | 2005 | Europe | 142 | 0.72 | 44.1 | escitalopram | 20 | MADRS | 36.3 | ①② |
|  |  |  | 152 | 0.62 | 46.2 | citalopram | 40 | MADRS | 35.7 | ①② |
| Moscovitch^124^ | 2004 | Europe | 93 | 0.77 | 39.6 | sertraline | 200 | HAMD17 | 18.62 | ①② |
|  |  |  | 94 | 0.78 | 40 | placebo | 0 | HAMD17 | 17.76 | ①② |
| Munizza^125^ | 2006 | Europe | 60 | 0.7 | 46.9 | sertraline | 100 | HAMD17 | 21.9 | ①② |
|  |  |  | 62 | 0.6 | 45 | trazodone | 450 | HAMD17 | 21.7 | ①② |
| MY-1008/BRL-029060/2/CPMS-076^126^ |  | Europe | 4 | 1 | 49.3 | paroxetine | 30 | HAMD21 |  | ② |
|  |  |  | 4 | 0.5 | 46.5 | placebo | 0 | HAMD21 |  | ② |
| MY-1042/BRL-029060/CPMS-251^127^ |  | North America | 125 | 0.66 | 41.7 | paroxetine | 50 | HAMDunspecified | 24.47 | ①② |
|  |  |  | 129 | 0.65 | 42 | placebo | 0 | HAMDunspecified | 24.35 | ①② |
| MY-1043/BRL-029060/115^128^ |  | North America | 284 | 0.64 | 42.3 | paroxetine | 50 | HAMD21 |  | ①② |
|  |  |  | 289 | 0.65 | 41.7 | fluoxetine | 80 | HAMD21 |  | ①② |
|  |  |  | 118 | 0.72 | 42.1 | placebo | 0 | HAMD21 |  | ①② |
| MY-1045/BRL-029060/1 (PAR 128)^129^ |  | North America | 357 | 0.62 | 42.3 | paroxetine | 50 | HAMD21 | 25.6 | ①② |
|  |  |  | 351 | 0.61 | 40.6 | fluoxetine | 80 | HAMD21 | 25.6 | ①② |
|  |  |  | 140 | 0.71 | 43.3 | placebo | 0 | HAMD21 | 25.7 | ①② |
| NCT01020799^130^ |  | North America | 50 | 0.44 | 36.22 | escitalopram | 20 | HAMDunspecified | 24.4 | ① |
|  |  |  | 99 | 0.5 | 41.77 | placebo | 0 | HAMDunspecified | 24.6 | ① |
| NCT01145755^131^ |  | North America | 47 | 0.51 | 39.9 | duloxetine | 60 | MADRS |  | ①② |
|  |  |  | 44 | 0.68 | 38.4 | placebo | 0 | MADRS |  | ①② |
| NCT01254305^132^ |  | North America | 93 | 0.65 | 41.4 | placebo | 0 |  |  | ①② |
|  |  |  | 90 | 0.58 | 42.9 | levomilnacipran | 120 |  |  | ①② |
| NCT01255787 (EUCTR2010-022257-41, Lu AA21004/CCT-002, U1111-1117-6595, JapicCTI-101344, CTRI/2011/08/001963)^133^ |  | Cross-continental | 152 | 0.6 | 43.6 | placebo | 0 | MADRS | 31.6 | ①② |
|  |  |  | 144 | 0.68 | 44.2 | vortioxetine | 5 | MADRS | 31.6 | ①② |
|  |  |  | 150 | 0.62 | 45.7 | vortioxetine | 10 | MADRS | 31.8 | ①② |
|  |  |  | 154 | 0.6 | 44 | vortioxetine | 20 | MADRS | 31.7 | ①② |
| NCT01355081(japicCTI-111492, U1111-1120-9277)^134^ |  | Asia | 119 | 0.42 | 38.3 | vortioxetine | 5 | HAMD17 | 20.9 | ①② |
|  |  |  | 123 | 0.44 | 38.8 | vortioxetine | 10 | HAMD17 | 21.2 | ①② |
|  |  |  | 124 | 0.54 | 37.6 | placebo | 0 | HAMD17 | 21.5 | ①② |
| NCT01808612^135^ |  | Asia | 169 | 0.49 | 40.05 | fluoxetine | 20 | HAMD21 |  | ①② |
|  |  |  | 84 | 0.4 | 39.58 | fluoxetine | 40 | HAMD21 |  | ①② |
|  |  |  | 260 | 0.5 | 38.54 | placebo | 0 | HAMD21 |  | ①② |
| Nemeroff^136^ | 1995 | North America | 49 | 0.61 | 38.5 | fluvoxamine | 150 | HAMD21 | 24.57 | ①② |
|  |  |  | 48 | 0.61 | 41.2 | sertraline | 200 | HAMD21 | 23.15 | ①② |
| Nemeroff^137^ | 2007 | North America | 104 | 0.65 | 37.9 | venlafaxine | 225 | HAMD21 | 23.7 | ② |
|  |  |  | 102 | 0.56 | 40.4 | placebo | 0 | HAMD21 | 23.5 | ② |
|  |  |  | 102 | 0.69 | 40.1 | fluoxetine | 60 | HAMD21 | 23.7 | ② |
| Nierenberg^138^ | 2007 | North America | 274 | 0.68 | 43.3 | escitalopram | 10 | HAMD17 | 17.8 | ①② |
|  |  |  | 273 | 0.63 | 41.1 | duloxetine | 60 | HAMD17 | 17.6 | ①② |
|  |  |  | 137 | 0.64 | 42.5 | placebo | 0 | HAMD17 | 17.7 | ①② |
| Noguera^139^ | 1991 | Europe | 60 | 0.7 | 46 | clomipramine | 100 | HAMDunspecified | 24.6 | ② |
|  |  |  | 60 | 0.75 | 46.3 | fluoxetine | 40 | HAMDunspecified | 24.3 | ② |
| Oakes2012a (NCT00536471)^140^ | 2012 | North America | 257 | 0.6 | 42.42 | duloxetine | 60 | HAMD17 | 22.9 | ② |
|  |  |  | 127 | 0.61 | 43.7 | placebo | 0 | HAMD17 | 22.8 | ② |
| Olie^141^ | 1997 | Europe | 129 | 0.79 | 43.6 | sertraline | 200 | HAMD17 | 25.4 | ①② |
|  |  |  | 129 | 0.84 | 43.9 | placebo | 0 | HAMD17 | 25.5 | ①② |
| Ou^142^ | 2010 | Asia | 120 | 0.54 | 36.4 | citalopram | 40 | HAMD17 | 22.9 | ② |
|  |  |  | 120 | 0.58 | 36.7 | escitalopram | 20 | HAMD17 | 23.4 | ② |
| PAR 29060.314 (HP 82/134)^143^ |  | Europe | 10 | 0.3 | 46.8 | paroxetine | 30 |  |  | ① |
|  |  |  | 8 | 0.75 | 41.8 | amitriptyline | 150 |  |  | ① |
| PAR 29060.316 (HP/82/47A)^144^ |  | Europe | 9 | 0.78 | 47.2 | paroxetine | 30 |  |  | ①② |
|  |  |  | 8 | 1 | 41 | amitriptyline | 150 |  |  | ①② |
| PAR 29060.318 (HP/82/64A)^145^ |  | Europe | 9 | 1 | 47.3 | paroxetine | 30 |  |  | ①② |
|  |  |  | 12 | 1 | 50.1 | amitriptyline | 150 |  |  | ①② |
| Patris^146^ | 1996 | Europe | 184 | 0.76 | 43 | fluoxetine | 20 | MADRS | 29.4 | ② |
|  |  |  | 173 | 0.79 | 44 | citalopram | 20 | MADRS | 29.7 | ② |
| Perahia^147^ | 2008 | Cross-continental | 166 | 0.72 | 45.42 | duloxetine | 60 | HAMD17 | 23.1 | ①② |
|  |  |  | 166 | 0.69 | 42.62 | venlafaxine | 150 | HAMD17 | 23.1 | ①② |
| Perahia^147^ | 2008 | Cross-continental | 164 | 0.67 | 43.07 | duloxetine | 60 | HAMD17 | 22.21 | ①② |
|  |  |  | 171 | 0.62 | 40.64 | venlafaxine | 150 | HAMD17 | 22.24 | ①② |
| Pomara^45^ | 2013 | North America | 255 | 0.51 | 39.3 | vilazodone | 40 | MADRS | 30.6 | ② |
|  |  |  | 253 | 0.56 | 41.1 | placebo | 0 | MADRS | 30.9 | ② |
| Quera^148^ | 2010 | not specified | 71 | 0.68 | 41.3 | agomelatine | 50 | HAMD17 | 26.1 | ① |
|  |  |  | 67 | 0.61 | 41.4 | escitalopram | 20 | HAMD17 | 26.1 | ① |
| Raskin^149^ | 2007 | North America | 207 | 0.6 | 72.63 | duloxetine | 60 | HAMD17 | 18.76 | ①② |
|  |  |  | 104 | 0.58 | 73.33 | placebo | 0 | HAMD17 | 18.94 | ①② |
| Rickels^150^ | 1994 | North America | 96 | 0.63 | 44.7 | nefazodone | 600 | HRSD | 24.3 | ①② |
|  |  |  | 95 | 0.63 | 42.6 | placebo | 0 | HRSD | 23.5 | ①② |
| Roose^151^ | 2004 | North America | 84 | 0.54 | 79.8 | citalopram | 20 | HAMD | 24.4 | ①② |
|  |  |  | 90 | 0.62 | 79.3 | placebo | 0 | HAMD | 24.2 | ①② |
| Rudolph^152^ | 1999 | North America | 103 | 0.69 | 40 | fluoxetine | 60 | HAMD | 26 | ① |
|  |  |  | 100 | 0.73 | 40 | venlafaxine | 225 | HAMD | 25 | ① |
|  |  |  | 98 | 0.64 | 40 | placebo | 0 | HAMD | 25 | ① |
| Rush^153^ | 1998 | North America | 67 |  |  | nefazodone | 500 |  |  | ①② |
|  |  |  | 64 |  |  | fluoxetine | 40 |  |  | ①② |
| Sacchetti^154^ | 2002 | Europe | 64 | 0.64 | 48.7 | paroxetine | 50 | HAMD | 25.4 | ①② |
|  |  |  | 65 | 0.66 | 50.5 | amitriptyline | 250 | HAMD | 26.2 | ①② |
| Sambunaris^155^ | 2014 | North America | 220 | 0.66 | 44.6 | placebo | 0 | HAMD | 22.9 | ② |
|  |  |  | 222 | 0.65 | 45 | levomilnacipran | 120 | HAMD | 23.3 | ② |
| Schatzberg^156^ | 2002 | North America | 128 | 0.5 | 71.7 | mirtazapine | 45 | HAMD | 22.2 | ①② |
|  |  |  | 126 | 0.53 | 72 | paroxetine | 40 | HAMD | 22.4 | ①② |
| Schatzberg^157^ | 2006 | North America | 100 | 0.45 | 71 | fluoxetine | 60 | HAMD | 24 | ①② |
|  |  |  | 104 | 0.56 | 71 | venlafaxine | 225 | HAMD | 24 | ①② |
|  |  |  | 96 | 0.46 | 71 | placebo | 0 | HAMD | 23 | ①② |
| Schneider^158^ | 2003 | North America | 371 | 0.54 | 70 | sertraline | 100 | HAMD | 21.4 | ①② |
|  |  |  | 376 | 0.58 | 69.6 | placebo | 0 | HAMD | 21.4 | ①② |
| Schone^159^ | 1993 | Europe | 54 | 0.83 | 74.3 | paroxetine | 50 | HAMD | 29 | ② |
|  |  |  | 52 | 0.9 | 73.7 | fluoxetine | 80 | HAMD | 27.9 | ② |
| Schwartz^160^ | 2002 | Cross-continental | 80 | 0.71 | 41.9 | reboxetine | 10 | HAMD | 28.2 | ② |
|  |  |  | 87 | 0.67 | 42.2 | venlafaxine | 375 | HAMD | 28.4 | ② |
| SCT-MD-49 (NCT00668525)^161^ | | North America | 322 | 0.64 | 41.4 | escitalopram | 10 | HAMD | 29.3 | ①② |
|  | |  | 332 | 0.69 | 40.4 | escitalopram | 30 | HAMD | 29.3 | ①② |
|  | |  | 220 | 0.63 | 42.3 | placebo | 0 | HAMD | 28.9 | ①② |
| Sechter^162^ | 1999 | Europe | 118 | 0.67 | 43.4 | sertraline | 150 | HAMD | 24.5 | ①② |
|  |  |  | 120 | 0.68 | 42.5 | fluoxetine | 60 | HAMD | 24.9 | ①② |
| Sechter^163^ | 2004 | Europe | 149 | 0.69 | 44.8 | milnacipran | 100 | HAMD | 23.7 | ② |
|  |  |  | 153 | 0.73 | 42.8 | paroxetine | 20 | HAMD | 23.4 | ② |
| Settle^164^ | 1999 | North America | 150 | 0.65 | 37.2 | bupropion | 300 | HAMD | 24.4 | ② |
|  |  |  | 154 | 0.64 | 38.2 | placebo | 0 | HAMD | 23.88 | ② |
| Sheehan^165^ | 2009 | North America | 99 | 0.69 | 37.8 | fluoxetine | 80 | HAMD | 29.5 | ② |
|  |  |  | 95 | 0.48 | 41.7 | venlafaxine | 375 | HAMD | 29.9 | ② |
|  |  |  | 95 | 0.64 | 39.9 | placebo | 0 | HAMD | 29.4 | ② |
| Sheehan^166^ | 2009 | North America | 206 | 0.64 | 43.8 | trazodone | 375 | HAMD | 23.2 | ① |
|  |  |  | 206 | 0.64 | 44 | placebo | 0 | HAMD | 22.4 | ① |
| Shu^167^ | 2014 | Asia | 314 | 0.68 | 39.2 | agomelatine | 50 | HAMD | 26.8 | ①② |
|  |  |  | 314 | 0.71 | 38.9 | fluoxetine | 40 | HAMD | 26.8 | ①② |
| Sramek^168^ | 1995 | not specified | 72 | 0.6 | 33.9 | fluoxetine | 20 | HAMD | 28.2 | ② |
|  |  |  | 72 | 0.6 | 33.9 | placebo | 0 | HAMD | 27.5 | ② |
| StudStudie009^169^ | | Europe | 26 | 0.65 | 45.81 | reboxetine | 8 | HAMD | 25.33 | ①② |
|  | |  | 24 | 0.67 | 47.04 | placebo | 0 | HAMD | 25.27 | ①② |
| Studie032^170^ | | Asia | 43 | 0.63 | 40.65 | reboxetine | 10 | HAMD | 27.23 | ①② |
|  | |  | 42 | 0.62 | 35.98 | fluoxetine | 40 | HAMD | 28.33 | ①② |
| Study015^171^ | | Cross-continental | 112 | 0.63 | 45.9 | reboxetine | 10 | HAMD | 27.5 | ①② |
|  | |  | 112 | 0.48 | 43.3 | placebo | 0 | HAMD | 27.1 | ①② |
| Study043^172^ | 2006 | Europe | 183 | 0.69 | 42.8 | reboxetine | 10 | HAMD | 27.4 | ①② |
|  |  |  | 176 | 0.6 | 41.5 | citalopram | 40 | HAMD | 27.4 | ①② |
| Study045^173^ | | Cross-continental | 87 | 0.7 | 42.3 | reboxetine | 2 | HAMD | 26.3 | ② |
|  | |  | 87 | 0.63 | 40.8 | reboxetine | 4 | HAMD | 26.2 | ② |
|  | |  | 89 | 0.63 | 41.6 | reboxetine | 8 | HAMD | 26.4 | ② |
|  | |  | 87 | 0.7 | 40.5 | placebo | 0 | HAMD | 26.4 | ② |
| Study049^174^ | | North America | 107 | 0.55 | 39.9 | reboxetine | 10 | HAMD | 25.1 | ①② |
|  | |  | 105 | 0.58 | 39.7 | placebo | 0 | HAMD | 25.3 | ①② |
| Study F1J‐MC‐ HMAQ ‐ Study Group B^175^ | | North America | 82 | 0.68 | 39.86 | duloxetine | 120 | HAMD | 17.88 | ①② |
|  | |  | 75 | 0.67 | 41.39 | placebo | 0 | HAMD | 18.32 | ①② |
|  | |  | 37 | 0.62 | 39.65 | fluoxetine | 20 | HAMD | 20.03 | ①② |
| Thase^176^ | 2006 | North America | 171 | 0.56 | 37.1 | bupropion | 450 | HAMD | 24.9 | ① |
|  |  |  | 177 | 0.64 | 37.4 | venlafaxine | 225 | HAMD | 24.1 | ① |
| Tollefson^177^ | 1995 | North America | 335 | 0.54 | 67.4 | fluoxetine | 20 | HAMD | 22.2 | ② |
|  |  |  | 336 | 0.55 | 68.1 | placebo | 0 | HAMD | 22.1 | ② |
| Tourian^178^ | 2009 | North America | 155 | 0.69 | 41 | desvenlafaxine | 50 | HAMD | 23 | ①② |
|  |  |  | 160 | 0.66 | 39 | desvenlafaxine | 100 | HAMD | 23 | ①② |
|  |  |  | 159 | 0.66 | 39 | duloxetine | 60 | HAMD | 23 | ①② |
|  |  |  | 164 | 0.58 | 39 | placebo | 0 | HAMD | 24 | ①② |
| Tzanakaki^179^ | 2000 | Europe | 54 | 0.75 | 49 | fluoxetine | 60 | HAMD | 27.1 | ② |
|  |  |  | 55 | 0.83 | 47 | venlafaxine | 225 | HAMD | 27.8 | ② |
| Vanmoffaert^180^ | 1995 | Europe | 100 | 0.69 | 46.1 | mirtazapine | 56 | HAMD | 29.2 | ① |
|  |  |  | 100 | 0.71 | 46.3 | trazodone | 350 | HAMD | 27.5 | ① |
| Ventura^181^ | 2007 | North America | 107 | 0.55 | 40.6 | escitalopram | 10 | HAMD | 26.8 | ①② |
|  |  |  | 108 | 0.6 | 38.1 | sertraline | 200 | HAMD | 26.8 | ①② |
| Versiani^182^ | 1999 | South America | 77 | 0.76 | 41.3 | fluoxetine | 20 | HAMD | 28.4 | ①② |
|  |  |  | 80 | 0.76 | 41.3 | amitriptyline | 300 | HAMD | 27.8 | ①② |
| Versiani^183^ | 2000 | Cross-continental | 28 | 0.54 | 41.7 | reboxetine | 10 | HAMD | 35.7 | ② |
|  |  |  | 28 | 0.5 | 40 | placebo | 0 | HAMD | 35.1 | ② |
| Versiani^184^ | 2005 | Cross-continental | 145 | 0.74 | 43 | mirtazapine | 60 | HAMD | 29 | ①② |
|  |  |  | 149 | 0.69 | 47 | fluoxetine | 40 | HAMD | 28 | ①② |
| Wade^185^ | 2003 | Europe | 99 | 0.75 | 40 | mirtazapine | 45 | HAMD | 23.8 | ①② |
|  |  |  | 98 | 0.71 | 40 | paroxetine | 30 | HAMD | 24.4 | ①② |
| Wade^186^ | 2007 | Cross-continental | 144 | 0.74 | 43.4 | escitalopram | 20 | HAMD | 22.7 | ①② |
|  |  |  | 151 | 0.7 | 44.5 | duloxetine | 60 | HAMD | 22.7 | ①② |
| Wang^187^ | 2014 | Cross-continental | 157 | 0.76 | 40.3 | escitalopram | 20 | HAMD | 27.2 | ①② |
|  |  |  | 157 | 0.67 | 39.7 | placebo | 0 | HAMD | 26.6 | ①② |
| Wang^188^ | 2015 | Asia | 213 | 0.58 | 40 | vortioxetine | 10 | MADRS | 32.3 | ② |
|  |  |  | 230 | 0.62 | 41 | venlafaxine | 150 | MADRS | 32.3 | ② |
| Weisler^189^ | 1994 | North America | 63 |  | 40.2 | bupropion | 450 | HAMD | 25.8 | ①② |
|  |  |  | 61 |  | 40.8 | trazodone | 400 | HAMD | 24.9 | ①② |
| WELL AK140016^190^ | | North America | 69 | 0.46 | 37.6 | bupropion | 300 | HAMD | 21.9 | ①② |
|  | |  | 71 | 0.52 | 37 | paroxetine | 20 | HAMD | 22.3 | ①② |
| Wilcox^191^ | 1994 | not specified | 50 | 0.48 | 40 | amitriptyline | 300 | HAMD | 25.8 | ① |
|  |  |  | 49 | 0.47 | 40 | placebo | 0 | HAMD | 25.5 | ① |
| Yevtushenko^192^ | 2007 | Europe | 108 | 0.61 | 35.19 | escitalopram | 10 | MADRS | 34.78 | ① |
|  |  |  | 106 | 0.58 | 34.79 | citalopram | 10 | MADRS | 35.4 | ① |
|  |  |  | 108 | 0.57 | 35.12 | citalopram | 20 | MADRS | 35.7 | ① |
| Zajecka^193^ | 2010 | North America | 170 | 0.69 | 44.2 | agomelatine | 25 | HAMD | 26.7 | ①② |
|  |  |  | 168 | 0.63 | 44.2 | agomelatine | 50 | HAMD | 27.1 | ①② |
|  |  |  | 173 | 0.68 | 43.1 | placebo | 0 | HAMD | 27.1 | ①② |
| Zhang^194^ | 2014 | Asia | 192 | 0.62 | 39.5 | trazodone | 450 | HAMD | 21.6 | ① |
|  |  |  | 190 | 0.61 | 38.3 | placebo | 0 | HAMD | 21.9 | ① |
| 29060/299^195^ | | Europe | 109 | 0.62 | 40.6 | paroxetine | 50 | HAMD | 27.52 | ① |
|  | |  | 108 | 0.56 | 40.27 | amitriptyline | 250 | HAMD | 28.16 | ① |
| 29060/356^196^ | | Oceania | 68 | 0.56 | 42.2 | paroxetine | 20 | HAMD | 24.6 | ①② |
|  | |  | 70 | 0.81 | 40.6 | fluoxetine | 20 | HAMD | 23.8 | ①② |
| Borhannejad^197^ | 2020 | North America | 25 | 0.8 | 71.84 | vortioxetine | 15 | HAMD | 30.6 | ①② |
|  |  |  | 25 | 0.68 | 69.44 | sertraline | 75 | HAMD | 30.88 | ①② |
| Emsley^198^ | 2018 | Cross-continental | 107 | 0.66 | 70.8 | placebo | 0 | HRSD | 26.7 | ② |
|  |  |  | 99 | 0.66 | 70.3 | escitalopram | 10 | HRSD | 26.6 | ② |
| Fagiolini^199^ | 2020 | Oceania | 158 | 0.78 | 47.9 | trazodone | 225 | MADRS | 43.8 | ① |
|  |  |  | 166 | 0.73 | 47.8 | venlafaxine | 450 | MADRS | 45.8 | ① |
| Inoue^200^ | 2020 | Asia | 161 | 0.44 | 39.5 | placebo | 0 | MADRS | 30.8 | ① |
|  |  |  | 165 | 0.44 | 40 | vortioxetine | 10 | MADRS | 30.6 | ① |
|  |  |  | 163 | 0.49 | 40.4 | vortioxetine | 20 | MADRS | 30.5 | ① |
| Kennedy^201^ | 2016 | North America | 133 | 0.79 | 45.4 | agomelatine | 10 | SDS | 21.5 | ① |
|  |  |  | 138 | 0.65 | 46.5 | agomelatine | 25 | SDS | 22 | ① |
|  |  |  | 137 | 0.72 | 43.1 | agomelatine | 50 | SDS | 22.4 | ① |
|  |  |  | 141 | 0.77 | 45 | placebo | 0 | SDS | 21.6 | ① |
| Nishimura^202^ | 2018 | Cross-continental | 152 | 0.59 | 43.6 | placebo | 0 | MADRS | 31.6 | ② |
|  |  |  | 144 | 0.32 | 44.2 | vortioxetine | 5 | MADRS | 31.6 | ② |
|  |  |  | 150 | 0.38 | 45.7 | vortioxetine | 10 | MADRS | 31.8 | ② |
|  |  |  | 154 | 0.4 | 44 | vortioxetine | 20 | MADRS | 31.7 | ② |
| Udristoiu^203^ | 2016 | Europe | 144 | 0.84 | 47.1 | agomelatine | 25 | HAMD17 | 26.1 | ①② |
|  |  |  | 143 | 0.8 | 46.4 | escitalopram | 10 | HAMD17 | 26.1 | ①② |
| Wang^204^ | 2019 | Asia | 60 | 0.65 | 45.8 | vortioxetine | 20 | MADRS | 33.8 | ② |
|  |  |  | 60 | 0.7 | 47.1 | placebo | 0 | MADRS | 34.1 | ② |
| LvYe^205^ | 2021 | Asia | 184 | 0.73 | 29.5 | toludesvenlafaxine | 160 | HAMD | 21.9 | ① |
|  |  |  | 184 | 0.72 | 29.4 | toludesvenlafaxine | 80 | HAMD | 22.4 | ① |
|  |  |  | 184 | 0.73 | 29.7 | placebo | 0 | HAMD | 21.7 | ① |
| Lin^206^ | 2022 | Asia | 39 | 0.87 | 66.9 | sertraline | 150 | HAMD | 27.0 | ①② |
|  |  |  | 39 | 0.74 | 70.5 | placebo | 0 | HAMD | 25.4 | ①② |
| NCT02623205^207^ | 2022 | North America | 31 | 0.64 | 31.7 | escitalopram | 30 | HAMD | 18.9 | ①② |
|  |  |  | 32 | 0.67 | 29.1 | placebo | 0 | HAMD | 16.8 | ①② |
| NCT02332291^208^ | 2021 | North America | 63 | 0.57 | 66.4 | escitalopram | 20 | MADRS | 26.3 | ② |
|  |  |  | 32 | 0.63 | 66.4 | bupropion | 450 | MADRS | 25.8 | ② |

1. Somnolence

② Insomnia

Table S3 Risk of bias assessment for individual studies

| studyID | Randomization process | Deviations from intended interventions | Missing outcome data | Measurement of the outcome | Selection of the reported result | Overall bias |
| --- | --- | --- | --- | --- | --- | --- |
| Aberg-Wisted2000 | Some concerns | Low | Low | Low | Some concerns | Some concerns |
| Aguglia1993 | Some concerns | Low | High | Low | Low | High |
| AK11023652009 | Some concerns | Low | High | Low | Low | High |
| Alvarez2012 | Low | Low | High | Low | Low | High |
| Amini2005 | Some concerns | Low | Low | Some concerns | Low | Some concerns |
| Ansseau1989a | Some concerns | Low | High | Some concerns | Some concerns | High |
| Ansseau1989b | Some concerns | Low | Low | Some concerns | Low | Some concerns |
| Ansseau1991 | Some concerns | Low | High | Some concerns | Low | High |
| Ansseau1994b | Some concerns | Low | Some concerns | Some concerns | Low | Some concerns |
| Ansseau1994c | Some concerns | Low | High | Some concerns | Low | High |
| Asnis2013 | Some concerns | Low | High | Low | Low | High |
| Bakish1992 | Some concerns | Low | High | Some concerns | Low | High |
| Baldwin2006 | Some concerns | Low | Some concerns | Some concerns | Low | Some concerns |
| Baldwin2012 | Low | Low | Some concerns | Low | Low | Some concerns |
| Ban1998 | Some concerns | Low | Low | Some concerns | Low | Some concerns |
| Battegay1985 | Some concerns | Low | High | Some concerns | Some concerns | High |
| Behnke2003 | Some concerns | Low | Some concerns | Some concerns | Low | Some concerns |
| Berlanga1997 | Some concerns | Low | High | Some concerns | Low | High |
| Bignamini1992 | Some concerns | Low | Low | Some concerns | Low | Some concerns |
| Blacker1988 | Some concerns | Low | Some concerns | Some concerns | Low | Some concerns |
| Andreoli2002 | Some concerns | Low | Low | Some concerns | Low | High |
| Bose2008 | Some concerns | Low | Some concerns | Some concerns | Low | Some concerns |
| Bougerol1997 | Some concerns | Low | Some concerns | Some concerns | Low | Some concerns |
| Study 0491998 | Some concerns | Low | High | Some concerns | Low | High |
| Boyer2008 | Some concerns | Low | Low | Some concerns | Low | Some concerns |
| Brunoni2012 | Low | Low | Low | Some concerns | Some concerns | Some concerns |
| Byerley1988 | Some concerns | Low | Some concerns | Some concerns | Low | Some concerns |
| CAGO178A23032008 | Some concerns | Low | Some concerns | Some concerns | Low | Some concerns |
| Chouinard1985 | Some concerns | Low | Low | Some concerns | Low | Some concerns |
| Chouinard1998 | Some concerns | Low | Low | Some concerns | Low | Some concerns |
| CL3-20098-022 | Low | Low | High | Some concerns | Low | High |
| CL3-20098-023 | Low | Low | Low | Some concerns | Low | Some concerns |
| CL3-20098-026 | Low | Low | Some concerns | Some concerns | Low | Some concerns |
| CL3-20098-062 | Low | Low | Some concerns | Some concerns | Low | Some concerns |
| CL3-20098-070 | Low | Low | Some concerns | Some concerns | Low | Some concerns |
| Claghorn1983 | Some concerns | Low | Some concerns | Some concerns | Low | Some concerns |
| Claghorn1995 | Some concerns | Low | High | Some concerns | Low | High |
| Claghorn1996 | Some concerns | Low | Some concerns | Some concerns | Low | Some concerns |
| Clerc1994 | Some concerns | Low | High | Some concerns | Low | High |
| Clerc2001 | Some concerns | Low | Some concerns | Some concerns | Low | Some concerns |
| Cohn1985 | Some concerns | Low | High | Some concerns | Low | High |
| Coleman1999 | Some concerns | Low | High | Some concerns | Low | High |
| Coleman2001 | Some concerns | Low | Some concerns | Some concerns | Low | Some concerns |
| Corrigan2000 | Some concerns | Low | High | Some concerns | Low | High |
| Corruble2013 | Low | Low | Low | Some concerns | Low | Some concerns |
| Croft1999 | Some concerns | Low | Some concerns | Low | Low | Some concerns |
| Croft2014 | Some concerns | Low | Low | Some concerns | Low | Some concerns |
| Cunningham1994 | Some concerns | Low | Low | Some concerns | Low | Some concerns |
| Cunningham1997 | Some concerns | Low | Some concerns | Some concerns | Low | Some concerns |
| Blier2009 | Some concerns | Low | Some concerns | Some concerns | Low | High |
| DeMartinis2007 | Some concerns | Low | Some concerns | Some concerns | Low | Some concerns |
| Ronchi1998 | Some concerns | Low | Some concerns | Some concerns | Low | Some concerns |
| Detke2002 | Some concerns | Low | Some concerns | Some concerns | Low | Some concerns |
| Detke2004 | Some concerns | Low | Some concerns | Some concerns | Low | Some concerns |
| Dierick1996 | Some concerns | Low | Some concerns | Some concerns | Low | Some concerns |
| Dimidjian2006 | Some concerns | Low | Some concerns | Low | Low | Some concerns |
| Doogan1994 | Low | Low | Some concerns | Some concerns | Some concerns | Some concerns |
| Dube2010 | Some concerns | Low | Some concerns | Some concerns | Low | Some concerns |
| Dunbar1993 | Some concerns | Low | Some concerns | Some concerns | Low | Some concerns |
| Fabre1995 | Some concerns | Low | Some concerns | Some concerns | Some concerns | Some concerns |
| Fabre1996 | Some concerns | Low | Some concerns | Low | Low | Some concerns |
| Falk1989 | Some concerns | Low | High | Some concerns | Low | High |
| Fava2002 | Some concerns | Low | Some concerns | Some concerns | Low | Some concerns |
| Fava2005 | Some concerns | Low | Some concerns | Some concerns | Low | Some concerns |
| Feighner1989a | Some concerns | Low | Some concerns | Some concerns | Low | Some concerns |
| Feighner1989b | Some concerns | Low | High | Some concerns | Low | High |
| Dunbar1991 | Some concerns | Low | Some concerns | Some concerns | Low | Some concerns |
| Gentil2000 | Some concerns | Low | Some concerns | Some concerns | Some concerns | Some concerns |
| Geretsegger1995 | Some concerns | Low | Some concerns | Some concerns | Low | Some concerns |
| Goldstein2004a | Some concerns | Low | Some concerns | Some concerns | Low | Some concerns |
| Goldstein2004b | Some concerns | Low | Some concerns | Some concerns | Low | Some concerns |
| Gommoll2014 | Low | Low | High | Low | Low | High |
| Goodarzi2015 | Some concerns | Low | High | Some concerns | Low | High |
| Griebel2012a | Some concerns | Low | Some concerns | Low | Low | Some concerns |
| Guelfi1998 | Some concerns | Low | Some concerns | Low | Some concerns | Some concerns |
| Hale2010 | Some concerns | Low | High | Some concerns | Some concerns | High |
| Halikas1995 | Some concerns | Low | Low | Some concerns | Low | Some concerns |
| Hewett2008 | Some concerns | Low | Low | Some concerns | Low | Some concerns |
| Hewett2010 | Some concerns | Low | Low | Low | Some concerns | Some concerns |
| Hicks2002 | Some concerns | Low | Low | Low | Low | Some concerns |
| Higuchi2016 | Some concerns | Low | High | Some concerns | Low | High |
| Higuchi2011 | Some concerns | Low | High | Some concerns | Low | High |
| Hormazabal1985 | Some concerns | Low | Some concerns | Some concerns | High | Some concerns |
| Hoyberg1996 | Some concerns | Low | Some concerns | Some concerns | High | Some concerns |
| Hsu2011 | Some concerns | Low | High | Some concerns | Low | High |
| Itil1983 | Some concerns | Low | Some concerns | Some concerns | Low | Some concerns |
| Iwata2013 | Some concerns | Low | Low | Low | Low | Some concerns |
| Judd1993 | Some concerns | Low | High | Low | Low | High |
| Kamijima2013 | Low | Low | Low | Some concerns | Low | Some concerns |
| Kasper2005b | Some concerns | Low | Low | Some concerns | Low | Some concerns |
| Kasper2005a | Some concerns | Low | Some concerns | Some concerns | Some concerns | Some concerns |
| Kasper2012 | Some concerns | Low | Low | Low | Some concerns | Some concerns |
| Katona2012 | Some concerns | Low | Low | Some concerns | Low | Some concerns |
| Keegan1991 | Some concerns | Low | Some concerns | Low | Some concerns | Some concerns |
| Keller2006a | Low | Low | Low | Some concerns | Low | Some concerns |
| Keller2007 | Some concerns | Low | Some concerns | Some concerns | Some concerns | Some concerns |
| Kennedy2014 | Some concerns | Low | Low | Some concerns | Low | Some concerns |
| Khan1998 | Low | Low | Some concerns | Low | Low | Some concerns |
| Koshino2013 | Some concerns | Low | Some concerns | Some concerns | Some concerns | Some concerns |
| Kramer1998 | Some concerns | Low | High | Some concerns | Low | High |
| Kyle1998 | Some concerns | Low | Low | Some concerns | High | Some concerns |
| Lalit2004 | Some concerns | Low | Some concerns | Some concerns | Some concerns | Some concerns |
| Learned2012a | Low | Low | Low | Low | Low | Some concerns |
| Lecrubier1997 | Some concerns | Low | Low | Some concerns | Low | Some concerns |
| Lee2007 | Some concerns | Low | Low | Low | Low | Some concerns |
| Lemoine2004 | Some concerns | Low | Low | Some concerns | Some concerns | Some concerns |
| Lepine2000 | Some concerns | Low | Low | Some concerns | Low | Some concerns |
| Lepola2001 | Some concerns | Low | Low | Some concerns | Low | Some concerns |
| Lieberm2008 | Some concerns | Low | Some concerns | Some concerns | Low | Some concerns |
| Liebowit2008 | Some concerns | Low | Some concerns | Low | Some concerns | Some concerns |
| Liebowitz2013 | Some concerns | Low | Some concerns | Some concerns | Low | Some concerns |
| Loo2002 | Some concerns | Low | Some concerns | Some concerns | Low | Some concerns |
| Lydiard1997 | Some concerns | Low | Low | Low | Low | Some concerns |
| M/2020/0046 (Study 046) | Some concerns | Low | Some concerns | Low | Low | Some concerns |
| M/2020/0047 (Study 047) | Some concerns | Low | Some concerns | Some concerns | Some concerns | Some concerns |
| Mahableshwarkar2013 | Some concerns | Low | Some concerns | Some concerns | Some concerns | Some concerns |
| Mahableshwarkar2015 | Some concerns | Low | Some concerns | Some concerns | Some concerns | Some concerns |
| Mao2008 | Some concerns | Low | Some concerns | Some concerns | Low | Some concerns |
| Mao2015 | Some concerns | Low | High | Some concerns | Some concerns | Some concerns |
| Marchesi1998 | Low | Low | Low | Some concerns | Low | Some concerns |
| Mathews2015 | Some concerns | Low | Some concerns | Some concerns | Some concerns | Some concerns |
| McGrath2000 | Some concerns | Low | Low | Low | Low | Some concerns |
| McPartlin1998 | Some concerns | Low | Some concerns | Some concerns | Low | Some concerns |
| Moller1993 | Low | Low | Some concerns | Some concerns | Low | Some concerns |
| Moller2000 | Some concerns | Low | Some concerns | Some concerns | Low | Some concerns |
| Montgomeryunpublished | Some concerns | Low | Some concerns | Some concerns | Low | Some concerns |
| Montgomery2004 | Some concerns | Low | Low | Low | Low | Some concerns |
| Moon1994 | Some concerns | Low | Some concerns | Some concerns | Low | Some concerns |
| Moore2005 | Some concerns | Low | Low | Some concerns | Low | Some concerns |
| Moscovitch2004 | Some concerns | Low | Low | Low | Low | Some concerns |
| Munizza2006 | Some concerns | Low | Some concerns | Some concerns | Low | Some concerns |
| MY-1008/BRL-029060/2/CPMS-076unpublished | Some concerns | Low | Some concerns | Some concerns | Some concerns | Some concerns |
| MY-1042/BRL-029060/CPMS-251unpublished | Some concerns | Low | Low | Low | Low | Some concerns |
| MY-1043/BRL-029060/115unpublished | Some concerns | Low | Some concerns | Some concerns | Some concerns | Some concerns |
| MY-1045/BRL-029060/1 (PAR 128) unpublished | Some concerns | Low | Some concerns | Some concerns | Some concerns | Some concerns |
| NCT01020799 | Some concerns | Low | Low | Low | Low | Some concerns |
| NCT01145755 | Some concerns | Low | High | Some concerns | Low | High |
| NCT01254305 | Some concerns | Low | Some concerns | Some concerns | Low | Some concerns |
| NCT01255787 (EUCTR2010-022257-41, Lu AA21004/CCT-002, U1111-1117-6595, JapicCTI-101344, CTRI/2011/08/001963) unpublished | Some concerns | Low | Some concerns | Some concerns | Low | Some concerns |
| NCT01355081(japicCTI-111492, U1111-1120-9277) unpublished | Low | Low | Some concerns | Some concerns | Low | Some concerns |
| NCT01808612 | Some concerns | Low | Some concerns | Some concerns | Low | Some concerns |
| Nemeroff1995 | Low | Low | High | Some concerns | Low | Some concerns |
| Nemeroff2007 | Some concerns | Low | Some concerns | Some concerns | Low | Some concerns |
| Nierenberg2007 | Low | Low | Some concerns | Low | Some concerns | Some concerns |
| Noguera1991 | Low | Low | Low | Some concerns | Low | Some concerns |
| Oakes2012a (NCT00536471) | Some concerns | Low | High | Some concerns | Low | High |
| Olie1997 | Low | Low | Low | Some concerns | Low | Some concerns |
| Ou2010 | Some concerns | Low | High | Some concerns | Some concerns | High |
| PAR 29060.314 (HP 82/134) | Some concerns | Low | Low | Some concerns | Low | Some concerns |
| PAR 29060.316 (HP/82/47A) | Some concerns | Low | Some concerns | Some concerns | Some concerns | Some concerns |
| PAR 29060.318 (HP/82/64A) | Some concerns | Low | Some concerns | Some concerns | Low | Some concerns |
| Patris1996 | Some concerns | Low | Some concerns | Some concerns | Low | Some concerns |
| Perahia2008a | Some concerns | Some concerns | High | Some concerns | Some concerns | High |
| Perahia2008b | Low | Low | Low | Some concerns | Low | Some concerns |
| Pomara2013 | Some concerns | Low | High | Some concerns | Low | High |
| Quera-Salva2010 (CL3-20098-056) | Some concerns | Low | Some concerns | Some concerns | Low | Some concerns |
| Raskin2007 | Some concerns | Low | High | Some concerns | Low | High |
| Rickels1994 | Some concerns | Low | High | Some concerns | Low | High |
| Roose2004 | Some concerns | Low | Some concerns | Some concerns | Low | Some concerns |
| Rudolph1999 | Some concerns | Low | Some concerns | Some concerns | Low | Some concerns |
| Rush1998 | Some concerns | Low | Some concerns | Some concerns | Low | Some concerns |
| Sacchetti2002 | Some concerns | Low | High | Some concerns | Low | High |
| Sambunaris2014 | Low | Low | Some concerns | Some concerns | Low | Some concerns |
| Schatzberg2002 | Some concerns | Low | High | Some concerns | Low | High |
| Schatzberg2006 | Some concerns | Low | Low | Some concerns | Some concerns | Some concerns |
| Schneider2003 | Some concerns | Low | Some concerns | Low | Low | Some concerns |
| Schone1993 | Some concerns | Low | Some concerns | Some concerns | Some concerns | Some concerns |
| Schwartz2002 | Some concerns | Low | Some concerns | Some concerns | Low | Some concerns |
| SCT-MD-49 (NCT00668525) | Some concerns | Low | High | Some concerns | Low | High |
| Sechter1999 | Some concerns | Low | Low | Some concerns | Low | Some concerns |
| Sechter2004 | Some concerns | Low | Some concerns | Some concerns | Low | Some concerns |
| Settle1999 | Some concerns | Low | Low | Some concerns | Low | Some concerns |
| Sheehan2009a | Some concerns | Low | High | Some concerns | Low | High |
| Sheehan2009b | Some concerns | Low | High | Some concerns | Low | High |
| Shu2014 | Some concerns | Low | Low | Some concerns | Low | Some concerns |
| Sramek1995 | Some concerns | Low | High | Some concerns | High | High |
| Studie009 | Low | Low | High | Some concerns | Low | High |
| Studie032 | Low | Low | High | Some concerns | Low | High |
| Study015 | Some concerns | Low | Some concerns | Low | Low | Some concerns |
| Study043 | Some concerns | Low | Some concerns | Low | Low | Some concerns |
| Study045 | Some concerns | Low | Some concerns | Low | Low | Some concerns |
| Study049 | Some concerns | Low | Some concerns | Low | Low | Some concerns |
| F1J-MC-HMAQ | Low | Low | Low | Low | Low | Some concerns |
| Thase2006 | Some concerns | Low | Low | Some concerns | Some concerns | Some concerns |
| Tollefson1995 | Some concerns | Low | Some concerns | Some concerns | Low | Some concerns |
| Tourian2009 | Some concerns | Low | Low | Some concerns | Some concerns | Some concerns |
| Tzanakaki2000 | Some concerns | Low | Some concerns | Some concerns | Low | Some concerns |
| Vanmoffaert1995a | Some concerns | Low | High | Some concerns | Low | High |
| Ventura2007 | Some concerns | Low | Low | Some concerns | Low | Some concerns |
| Versiani1999 | Low | Low | Some concerns | Some concerns | Low | Some concerns |
| Versiani2000 | Some concerns | Low | High | Some concerns | Some concerns | High |
| Versiani2005 | Some concerns | Low | Some concerns | Some concerns | Low | Some concerns |
| Wade2003 | Some concerns | Low | Some concerns | Some concerns | Low | Some concerns |
| Wade2007 | Some concerns | Low | Some concerns | Some concerns | Some concerns | Some concerns |
| Wang2014 | Some concerns | Low | Some concerns | Some concerns | Low | Some concerns |
| Wang2015 | Some concerns | Low | Some concerns | Some concerns | Low | Some concerns |
| Weisler1994 | Some concerns | Low | High | Some concerns | Low | High |
| WELL AK140016 | Some concerns | Low | High | Some concerns | Low | High |
| Wilcox1994 | Low | Low | Some concerns | Some concerns | Low | Some concerns |
| Yevtushenko2007 | Some concerns | Low | Some concerns | Some concerns | Low | Some concerns |
| Zajecka2010 | Some concerns | Low | High | Low | Low | High |
| Zhang2014 | Some concerns | Low | Some concerns | Some concerns | Low | Some concerns |
| 29060/299 | Some concerns | Low | High | Some concerns | Low | High |
| 29060/356 | Some concerns | Low | Some concerns | Some concerns | Low | Some concerns |
| Borhannejad2020 | Some concerns | Low | High | Some concerns | Low | High |
| Emsley2018 | Low | Low | Low | Low | Low | Low |
| Fagiolini2020 | Low | Low | Low | Low | Low | Low |
| Inoue2020 | Low | Low | Low | Low | Low | Low |
| Kennedy2016 | Some concerns | Low | Low | Some concerns | Low | Some concerns |
| Nishimura2018 | Low | Low | Low | Some concerns | Low | Some concerns |
| Udristoiu2016 | Low | Low | Low | Low | Low | Low |
| Wang2019 | Low | Low | Low | Low | Low | Low |
| LvYe2021 | Low | Low | Low | Low | Low | Low |
| Lin2022 | Some concerns | Low | Some concerns | Some concerns | Some concerns | Some concerns |
| NCT02623205 | Some concerns | Low | Low | Low | Low | Some concerns |
| NCT02332291 | Low | Low | Low | Low | Low | Low |

Table S4 The estimations of meta-regression coefficients of age for somnolence

| Comparison | 2.5% | 50% | 97.5% |
| --- | --- | --- | --- |
| Escitalopram.Agomelatine | -0.7544 | 0.16202 | 1.124852 |
| Escitalopram.Citalopram | -2.2179 | -0.95735 | 0.282555 |
| Escitalopram.Duloxetine | -23.9863 | -0.07447 | 37.935153 |
| Escitalopram.Fluoxetine | -1.6821 | -0.36662 | 0.942979 |
| Escitalopram.Paroxetine | -63.2282 | -0.13783 | 49.500845 |
| Escitalopram.Placebo | -21.8189 | 0.07643 | 32.687697 |
| Escitalopram.Sertraline | -0.5276 | 0.68400 | 2.094115 |
| Escitalopram.Venlafaxine | -0.8842 | 1.25740 | 4.229692 |
| Fluoxetine.Amitriptyline | -1.4049 | -0.44887 | 0.456386 |
| Fluoxetine.Bupropion | -26.4538 | -0.04935 | 31.079981 |
| Fluoxetine.Milnacipran | -87.8177 | -0.30545 | 28.317054 |
| Fluoxetine.Mirtazapine | -34.5797 | 0.34245 | 50.981724 |
| Fluoxetine.Nefazodone | -4.2381 | -2.16508 | -0.228472 |
| Fluoxetine.Reboxetine | -70.8804 | -0.19628 | 27.013904 |
| Paroxetine.Trazodone | -3.6062 | -1.35987 | 0.718327 |
| Placebo.Desvenlafaxine | -50.6445 | -0.38904 | 30.928440 |
| Placebo.Fluvoxamine | -0.7819 | 0.21079 | 1.239899 |
| Placebo.Levomilnacipran | -50.1099 | 0.11149 | 92.733599 |
| Placebo.Toludesvenlafaxine | -0.7350 | 0.23300 | 1.190115 |
| Placebo.Vilazodone | -0.1166 | 0.78450 | 1.709991 |
| Placebo.Vortioxetine | -56.5582 | -0.19425 | 30.700162 |
| Sertraline.Clomipramine | -2.5740 | -0.25713 | 1.836714 |

Table S5 The estimations of meta-regression coefficients of percentage of females for somnolence

| Comparison | 2.5% | 50% | | 97.5% | |
| --- | --- | --- | --- | --- | --- |
| Escitalopram.Agomelatine | -0.61438 | | 0.806981 | | 2.2644 |
| Escitalopram.Citalopram | -0.86945 | | -0.183351 | | 0.4949 |
| Escitalopram.Duloxetine | -1.60987 | | -0.157081 | | 1.2678 |
| Escitalopram.Fluoxetine | -0.76289 | | 0.632510 | | 2.0670 |
| Escitalopram.Paroxetine | -43.90122 | | -0.297941 | | 22.3886 |
| Escitalopram.Placebo | -1.76563 | | 1.280864 | | 4.6112 |
| Escitalopram.Sertraline | -2.71362 | | -1.161995 | | 0.3108 |
| Escitalopram.Venlafaxine | -0.65898 | | 0.314264 | | 1.3193 |
| Fluoxetine.Amitriptyline | -1.25374 | | -0.421318 | | 0.4063 |
| Fluoxetine.Bupropion | -0.81250 | | 0.385520 | | 1.6607 |
| Fluoxetine.Milnacipran | -51.81818 | | -0.154519 | | 23.0026 |
| Fluoxetine.Mirtazapine | -2.09634 | | -0.500705 | | 1.1194 |
| Fluoxetine.Nefazodone | -1.46752 | | -0.587379 | | 0.2769 |
| Fluoxetine.Reboxetine | -3.80016 | | -1.522444 | | 0.6196 |
| Paroxetine.Trazodone | -0.74064 | | -0.173521 | | 0.3801 |
| Placebo.Desvenlafaxine | -0.47331 | | 0.842032 | | 2.2508 |
| Placebo.Fluvoxamine | -0.48180 | | 0.410065 | | 1.3443 |
| Placebo.Levomilnacipran | -16.89655 | | 0.440209 | | 53.6966 |
| Placebo.Toludesvenlafaxine | 0.03385 | | 2.415289 | | 5.1076 |
| Placebo.Vilazodone | -0.97191 | | -0.378645 | | 0.1982 |
| Placebo.Vortioxetine | -13.72184 | | 0.620660 | | 68.4721 |
| Sertraline.Clomipramine | -1.24174 | | -0.150633 | | 0.9407 |

Table S6 The estimations of meta-regression coefficients of baseline severity for somnolence

| Comparison | 2.5% | 50% | 97.2% |
| --- | --- | --- | --- |
| Escitalopram.Agomelatine | -1.32141 | 0.44365 | 2.16366 |
| Escitalopram.Citalopram | -0.26672 | 0.82813 | 1.96091 |
| Escitalopram.Duloxetine | -1.23033 | -0.12297 | 0.96926 |
| Escitalopram.Fluoxetine | -0.45674 | 0.69307 | 1.86912 |
| Escitalopram.Paroxetine | -49.53701 | -0.27620 | 31.05469 |
| Escitalopram.Placebo | -10.63067 | 0.59413 | 18.00251 |
| Escitalopram.Sertraline | -0.61495 | 0.04889 | 0.71114 |
| Escitalopram.Venlafaxine | -1.26112 | -0.45487 | 0.34650 |
| Fluoxetine.Amitriptyline | -1.31167 | -0.42719 | 0.46305 |
| Fluoxetine.Bupropion | -1.44580 | 0.01552 | 1.46153 |
| Fluoxetine.Milnacipran | -1.44122 | -0.23506 | 0.94705 |
| Fluoxetine.Mirtazapine | -0.20637 | 1.13357 | 2.50411 |
| Fluoxetine.Nefazodone | -14.21990 | 0.42426 | 17.06700 |
| Fluoxetine.Reboxetine | -0.59494 | 0.05544 | 0.69832 |
| Paroxetine.Trazodone | -2.04448 | 0.02350 | 1.98524 |
| Placebo.Desvenlafaxine | -0.63877 | 0.09982 | 0.84037 |
| Placebo.Fluvoxamine | -46.95080 | -0.18296 | 40.32788 |
| Placebo.Toludesvenlafaxine | 0.09043 | 1.32217 | 2.71168 |
| Placebo.Vilazodone | -1.29576 | -0.22995 | 0.81429 |
| Placebo.Vortioxetine | -34.03749 | -1.09619 | 10.77679 |
| Sertraline.Clomipramine | -0.92895 | 0.27648 | 1.45080 |

Table S7 The estimations of meta-regression coefficients of age for insomnia

| Comparison | 2.5% | 50% | 97.5% |
| --- | --- | --- | --- |
| Fluoxetine.Agomelatine | -0.60874 | -0.070973 | 0.46935 |
| Fluoxetine.Amitriptyline | -0.45467 | 0.461323 | 1.35635 |
| Fluoxetine.Bupropion | -1.22897 | 0.544931 | 2.30344 |
| Fluoxetine.Citalopram | -1.37243 | -0.620115 | 0.07283 |
| Fluoxetine.Clomipramine | -4.87123 | 1.680159 | 20.39640 |
| Fluoxetine.Duloxetine | -2.98539 | -0.256182 | 2.46162 |
| Fluoxetine.Escitalopram | -2.81528 | -1.235639 | 0.19344 |
| Fluoxetine.Milnacipran | -0.81460 | -0.350838 | 0.10894 |
| Fluoxetine.Mirtazapine | -0.38220 | -0.066657 | 0.24934 |
| Fluoxetine.Nefazodone | -2.31619 | 0.863556 | 4.53873 |
| Fluoxetine.Paroxetine | -2.79562 | 1.651546 | 7.75820 |
| Fluoxetine.Placebo | -6.27447 | 0.510824 | 9.59045 |
| Fluoxetine.Reboxetine | -0.86912 | 0.054174 | 0.97970 |
| Fluoxetine.Sertraline | -3.50983 | 0.756741 | 5.93951 |
| Fluoxetine.Trazodone | -0.94151 | -0.269989 | 0.39454 |
| Fluoxetine.Venlafaxine | -1.91900 | 0.442585 | 2.89212 |
| Placebo.Desvenlafaxine | -0.57211 | -0.151008 | 0.27245 |
| Placebo.Fluvoxamine | -2.98304 | -1.062189 | 0.47890 |
| Placebo.Levomilnacipran | -0.61603 | -0.027758 | 0.54919 |
| Placebo.Vilazodone | -12.47115 | -2.040903 | 4.03257 |
| Placebo.Vortioxetine | -1.73566 | -0.429651 | 0.72929 |

Table S8 The estimations of meta-regression coefficients of percentage of females for insomnia

| Comparison | 2.5% | 50% | 97.5% |
| --- | --- | --- | --- |
| Fluoxetine.Agomelatine | -1.274140 | 0.17614 | 1.65466 |
| Fluoxetine.Amitriptyline | -0.248794 | 0.63501 | 1.52223 |
| Fluoxetine.Bupropion | -0.370076 | 0.42438 | 1.23054 |
| Fluoxetine.Citalopram | -1.344342 | -0.36716 | 0.59621 |
| Fluoxetine.Clomipramine | -1.108475 | 3.89260 | 51.94345 |
| Fluoxetine.Duloxetine | -2.692081 | -0.63574 | 1.35455 |
| Fluoxetine.Escitalopram | -1.430729 | -0.42730 | 0.57314 |
| Fluoxetine.Milnacipran | -0.792545 | -0.09854 | 0.59874 |
| Fluoxetine.Mirtazapine | -0.612634 | -0.11342 | 0.37979 |
| Fluoxetine.Nefazodone | -1.840537 | -0.24085 | 1.36413 |
| Fluoxetine.Paroxetine | -1.234482 | 2.94745 | 8.77923 |
| Fluoxetine.Placebo | -3.595154 | 0.44791 | 4.71475 |
| Fluoxetine.Reboxetine | -0.862897 | 0.00917 | 0.88294 |
| Fluoxetine.Sertraline | -2.998492 | -0.96336 | 0.95148 |
| Fluoxetine.Trazodone | -0.469640 | -0.08491 | 0.28539 |
| Fluoxetine.Venlafaxine | -1.710719 | -1.00643 | -0.34053 |
| Placebo.Desvenlafaxine | -0.656073 | -0.13906 | 0.37253 |
| Placebo.Fluvoxamine | -8.655282 | -2.93348 | 0.90989 |
| Placebo.Levomilnacipran | 0.054263 | 0.60658 | 1.17158 |
| Placebo.Vilazodone | -7.876517 | -2.21569 | 1.85526 |
| Placebo.Vortioxetine | -1.336209 | -0.40769 | 0.46090 |

Table S9 The estimations of meta-regression coefficients of baseline severity for insomnia

| Comparison | 2.5% | 50% | 97.5% |
| --- | --- | --- | --- |
| Fluoxetine.Agomelatine | -1.48747 | 0.15515 | 1.79180 |
| Fluoxetine.Amitriptyline | -0.86814 | 0.52567 | 2.00927 |
| Fluoxetine.Bupropion | -0.43828 | 0.17827 | 0.79297 |
| Fluoxetine.Citalopram | -1.25559 | -0.20636 | 0.83227 |
| Fluoxetine.Clomipramine | -4.06618 | -0.21195 | 3.28544 |
| Fluoxetine.Duloxetine | -8.03105 | -0.62697 | 5.49155 |
| Fluoxetine.Escitalopram | -0.41296 | 0.04806 | 0.50437 |
| Fluoxetine.Milnacipran | -0.52765 | 0.03507 | 0.61525 |
| Fluoxetine.Mirtazapine | -0.51668 | 0.02333 | 0.56181 |
| Fluoxetine.Nefazodone | -1.21021 | 0.45183 | 2.14579 |
| Fluoxetine.Paroxetine | -10.44560 | -2.85248 | 1.91520 |
| Fluoxetine.Placebo | -1.08359 | 0.71275 | 2.58148 |
| Fluoxetine.Reboxetine | -2.27583 | -0.85360 | 0.51568 |
| Fluoxetine.Sertraline | -1.80529 | 0.09759 | 2.11025 |
| Fluoxetine.Trazodone | -0.41654 | 0.07251 | 0.56016 |
| Fluoxetine.Venlafaxine | -0.51014 | 0.32744 | 1.17779 |
| Placebo.Desvenlafaxine | -0.41016 | 0.01597 | 0.43894 |
| Placebo.Fluvoxamine | -6.30878 | -2.22935 | 1.06326 |
| Placebo.Levomilnacipran | -0.29059 | 0.23295 | 0.75346 |
| Placebo.Vilazodone | -27.22746 | -3.37413 | 8.82220 |
| Placebo.Vortioxetine | -1.69010 | -0.15742 | 1.21435 |

Table S10 Local inconsistency detection by node split approach

| Outcome | Comparisons with direct evidence only | Comparisons with both direct and indirect evidence | Comparisons with inconsistent direct and indirect evidence |
| --- | --- | --- | --- |
| Somnolence | 3 | 253 | 9 |
| Insomnia | 1 | 231 | 3 |

Table S11 Secondary outcomes reported by included studies

| Author | Year | agent | Dose (mg) | sample size (n) | bad dream (n) | Sleepwalk(n) | restless leg syndrome (n) | sleep disorders (n) | abnormal dreams (n) | sedation-related symptoms (n) | sleep length reduced (n) | yawn (n) |
| --- | --- | --- | --- | --- | --- | --- | --- | --- | --- | --- | --- | --- |
| Study 049 | 1998 | reboxetine | 10 | 107 | 2 |  |  |  |  |  |  |  |
| Study 049 | 1998 | placebo | 0 | 105 | 3 |  |  |  |  |  |  |  |
| Claghorn | 1996 | fluvoxamine | 150 | 50 |  |  |  |  | 1 |  |  |  |
| Claghorn | 1996 | placebo | 0 | 50 |  |  |  |  | 0 |  |  |  |
| Corruble | 2013 | escitalopram | 20 | 160 |  |  |  |  | 1 |  |  |  |
| Corruble | 2013 | agomelatine | 50 | 164 |  |  |  |  | 4 |  |  |  |
| Cunningham | 1997 | placebo | 0 | 100 |  |  |  |  | 0 |  |  |  |
| Cunningham | 1997 | venlafaxine | 75 | 203 |  |  |  |  | 7 |  |  |  |
| DeMartinis | 2007 | placebo | 0 | 120 |  |  |  |  | 4 |  |  | 0 |
| DeMartinis | 2007 | desvenlafaxine | 400 | 116 |  |  |  |  | 8 |  |  | 2 |
| DeMartinis | 2007 | desvenlafaxine | 200 | 116 |  |  |  |  | 7 |  |  | 6 |
| DeMartinis | 2007 | desvenlafaxine | 100 | 118 |  |  |  |  | 6 |  |  | 3 |
| Dunbar | 1993 | paroxetine | 50 | 170 |  |  |  |  |  |  |  | 8 |
| Dunbar | 1993 | placebo | 0 | 171 |  |  |  |  |  |  |  | 0 |
| Fava | 2002 | sertraline | 200 | 96 |  |  |  |  | 8 |  |  |  |
| Fava | 2002 | fluoxetine | 60 | 92 |  |  |  |  | 8 |  |  |  |
| Fava | 2002 | paroxetine | 60 | 96 |  |  |  |  | 7 |  |  |  |
| Dunbar | 1991 | paroxetine | 50 | 241 |  |  |  |  |  |  |  | 7 |
| Dunbar | 1991 | placebo | 0 | 244 |  |  |  |  |  |  |  | 0 |
| Kasper | 2005 | trazodone | 450 | 55 |  |  |  | 1 |  |  |  |  |
| Kasper | 2005 | paroxetine | 40 | 53 |  |  |  | 0 |  |  |  |  |
| Keller | 2007 | fluoxetine | 60 | 275 |  |  |  |  |  |  |  | 7 |
| Keller | 2007 | venlafaxine | 300 | 821 |  |  |  |  |  |  |  | 4 |
| Learned | 2012 | paroxetine | 30 | 166 |  |  |  | 6 |  |  |  |  |
| Learned | 2012 | placebo | 0 | 156 |  |  |  | 3 |  |  |  |  |
| M/2020/0046 (Study 046) |  | reboxetine | 10 | 265 |  |  |  | 1 | 7 |  |  |  |
| M/2020/0046 (Study 046) |  | placebo | 0 | 257 |  |  |  | 3 | 6 |  |  |  |
| M/2020/0046 (Study 046) |  | paroxetine | 40 | 265 |  |  |  | 7 | 4 |  |  |  |
| M/2020/0047 (Study 047) |  | reboxetine | 10 | 258 |  |  |  | 3 | 4 |  |  |  |
| M/2020/0047 (Study 047) |  | placebo | 0 | 254 |  |  |  | 2 | 4 |  |  |  |
| M/2020/0047 (Study 047) |  | paroxetine | 40 | 262 |  |  |  | 5 | 7 |  |  |  |
| NCT01355081(japicCTI-111492, U1111-1120-9277) |  | vortioxetine | 5 | 119 | 0 |  | 0 |  |  |  |  |  |
| NCT01355081(japicCTI-111492, U1111-1120-9277) |  | vortioxetine | 10 | 123 | 2 |  | 0 |  |  |  |  |  |
| NCT01355081(japicCTI-111492, U1111-1120-9277) |  | placebo | 0 | 124 | 1 |  | 1 |  |  |  |  |  |
| Perahia | 2008 | duloxetine | 60 | 166 |  |  |  |  | 17 |  |  | 22 |
| Perahia | 2008 | venlafaxine | 150 | 166 |  |  |  |  | 10 |  |  | 10 |
| Perahia | 2008 | duloxetine | 60 | 164 |  |  |  |  | 22 |  |  | 22 |
| Perahia | 2008 | venlafaxine | 150 | 171 |  |  |  |  | 17 |  |  | 11 |
| Study043 | 2006 | reboxetine | 10 | 183 |  |  |  |  |  |  | 13 |  |
| Study043 | 2006 | citalopram | 40 | 176 |  |  |  |  |  |  | 8 |  |
| Study045 |  | reboxetine | 2 | 87 |  |  |  | 2 |  |  |  |  |
| Study045 |  | reboxetine | 4 | 87 |  |  |  | 1 |  |  |  |  |
| Study045 |  | reboxetine | 8 | 89 |  |  |  | 0 |  |  |  |  |
| Study045 |  | placebo | 0 | 87 |  |  |  | 0 |  |  |  |  |
| Study049 |  | reboxetine | 10 | 107 |  |  |  |  | 2 |  |  |  |
| Study049 |  | placebo | 0 | 105 |  |  |  |  | 3 |  |  |  |
| Thase | 2006 | bupropion | 450 | 171 |  |  |  |  |  |  |  | 0 |
| Thase | 2006 | venlafaxine | 225 | 177 |  |  |  |  |  |  |  | 12 |
| Tourian | 2009 | desvenlafaxine | 50 | 155 |  |  |  |  | 2 |  |  | 2 |
| Tourian | 2009 | desvenlafaxine | 100 | 160 |  |  |  |  | 3 |  |  | 1 |
| Tourian | 2009 | duloxetine | 60 | 159 |  |  |  |  | 8 |  |  | 7 |
| Tourian | 2009 | placebo | 0 | 164 |  |  |  |  | 2 |  |  | 0 |
| Vanmoffaert | 1995 | mirtazapine | 56 | 100 |  |  |  | 4 |  |  |  |  |
| Vanmoffaert | 1995 | trazodone | 350 | 100 |  |  |  | 9 |  |  |  |  |
| Zajecka | 2010 | agomelatine | 25 | 170 |  |  |  |  | 5 |  |  |  |
| Zajecka | 2010 | agomelatine | 50 | 168 |  |  |  |  | 4 |  |  |  |
| Zajecka | 2010 | placebo | 0 | 173 |  |  |  |  | 9 |  |  |  |
| NCT02623205 | 2022 | escitalopram | 30 | 42 | 0 |  |  | 0 |  | 0 |  |  |
| NCT02623205 | 2022 | placebo | 0 | 42 | 0 |  |  | 0 |  | 0 |  |  |


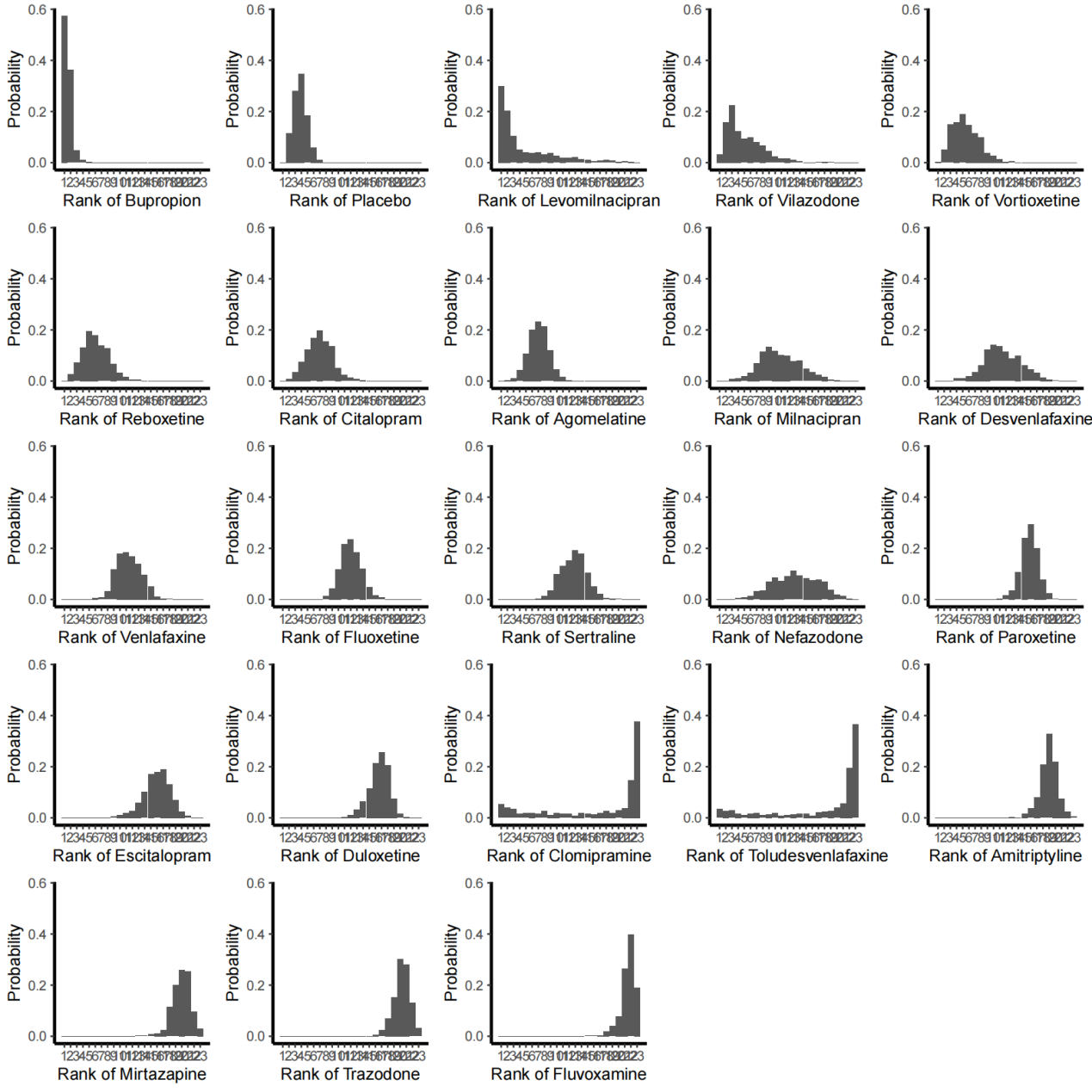


Figure S1 SUCRA for somnolence


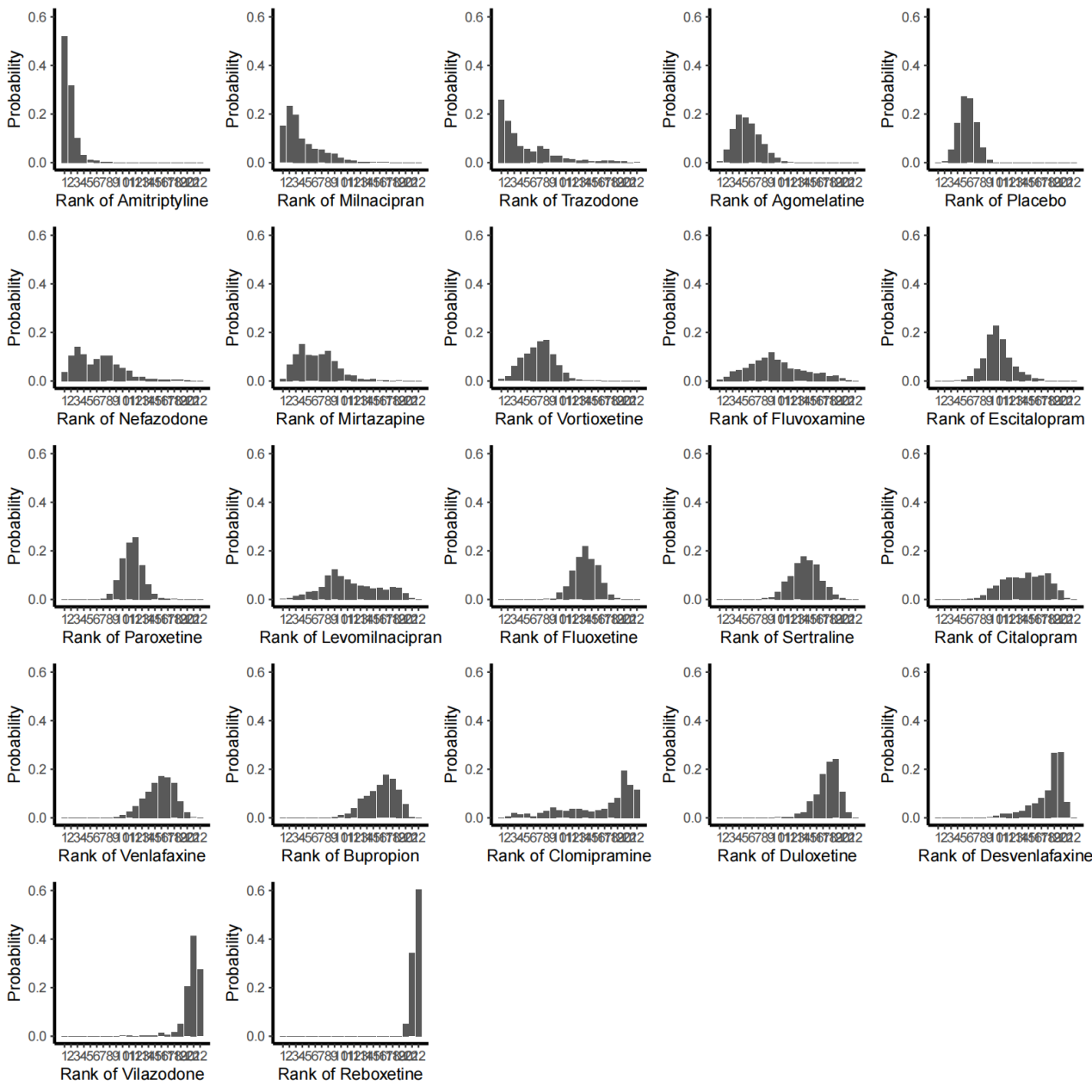


Figure S2 SUCRA for insomnia


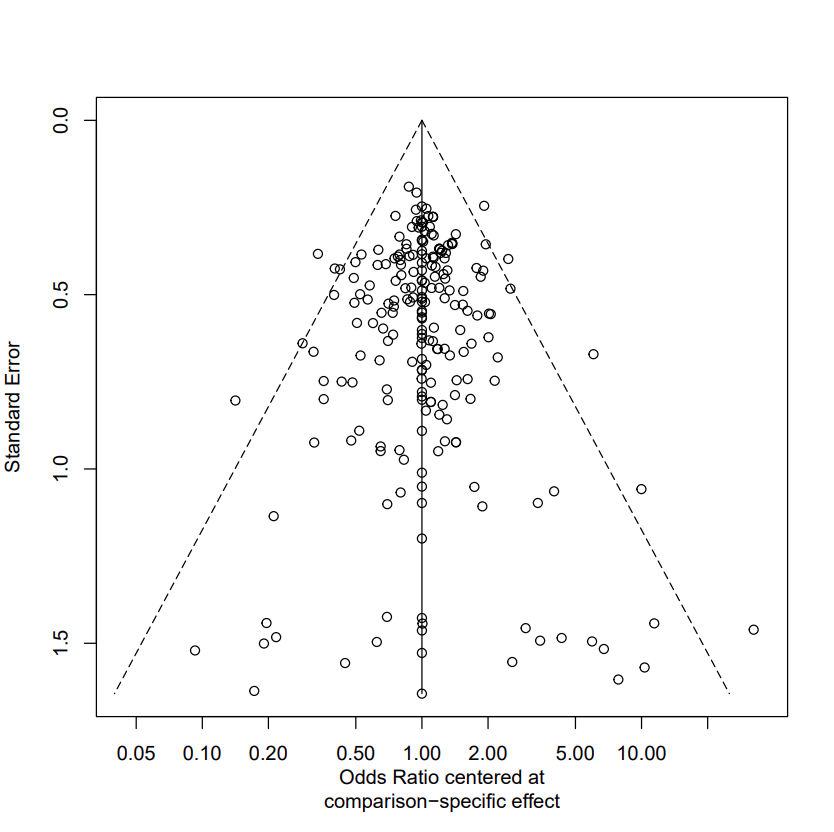


Figure S3 Funnel plot of somnolence


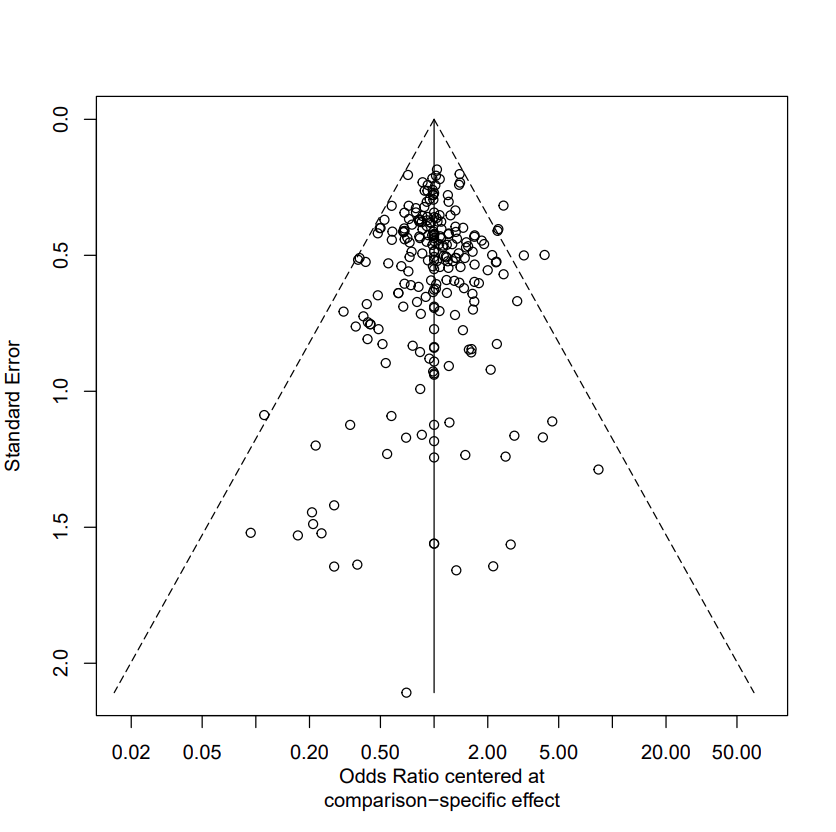


Figure S4 Funnel plot of insomnia


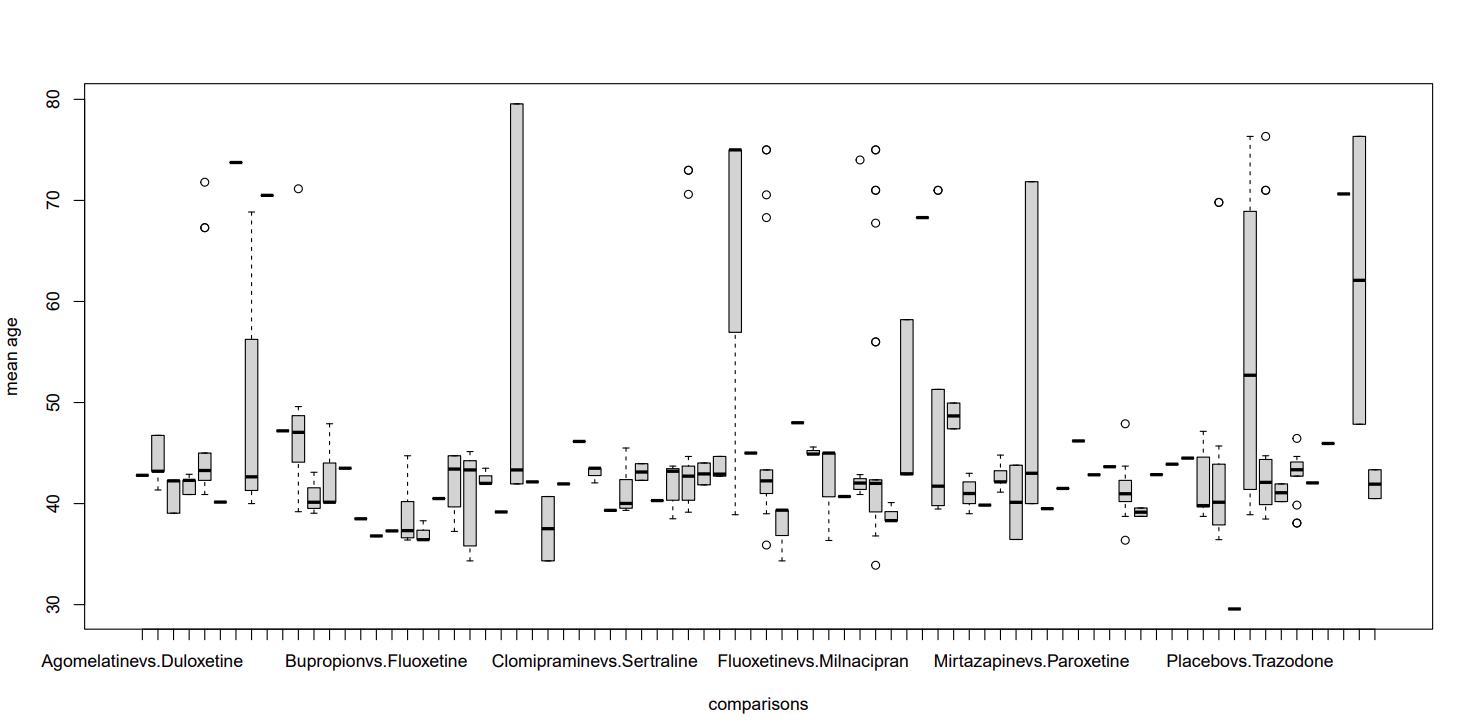


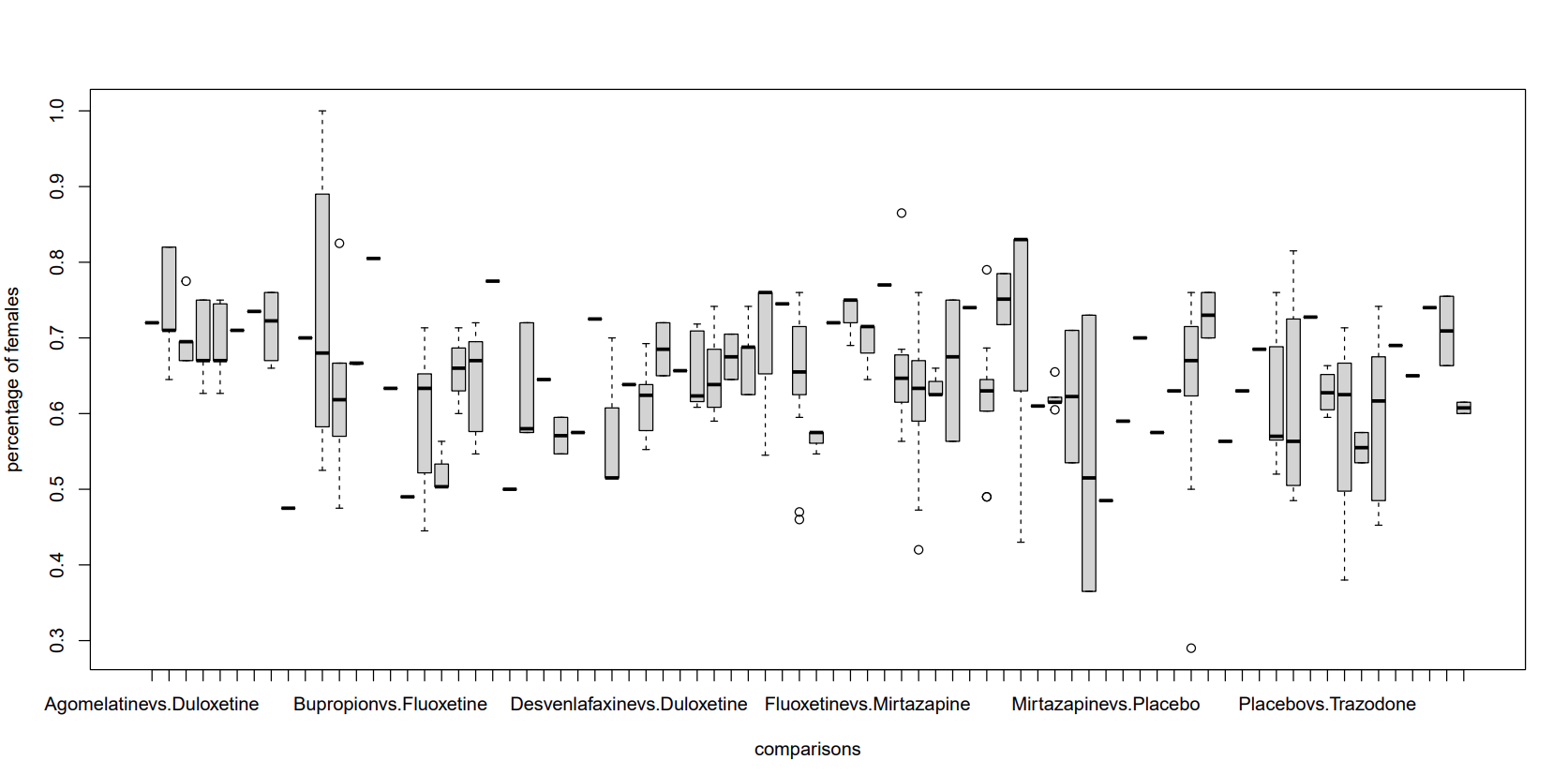


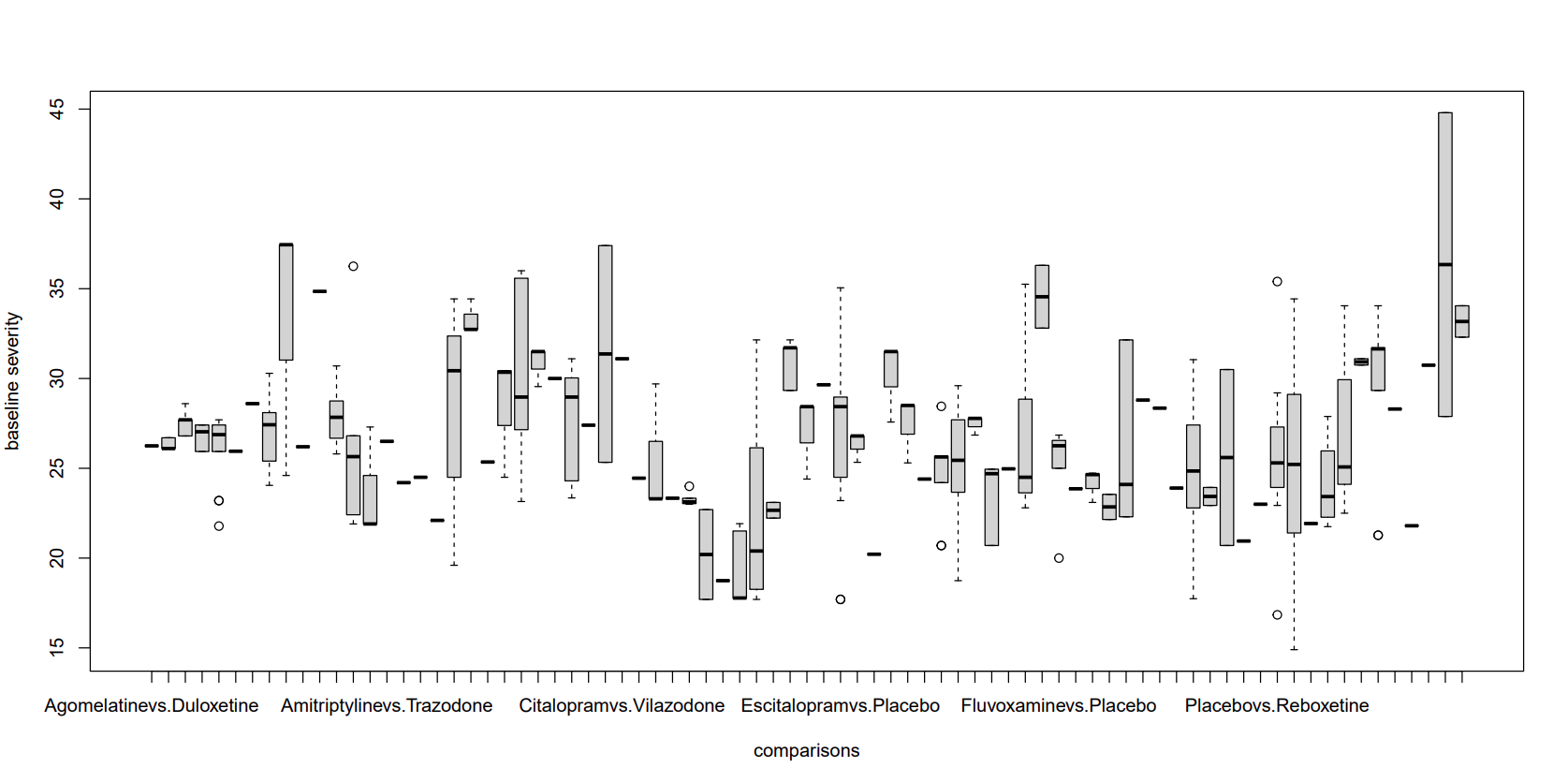


Figure S5 Illustrations for the similarity of baseline characteristics across comparisons


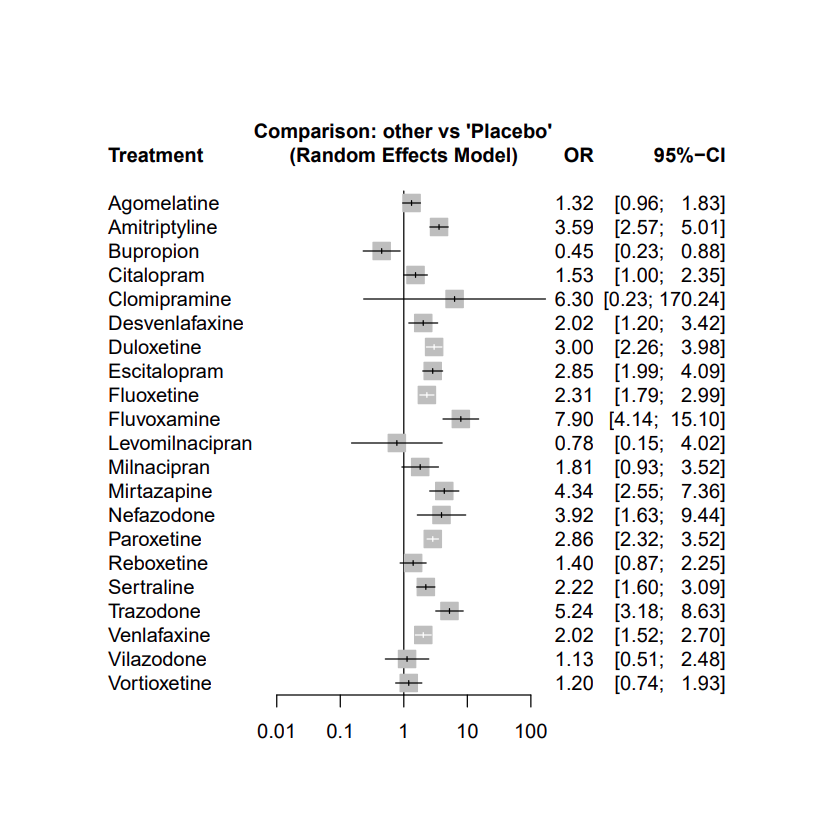


Figure S6 Result of sensitivity analyses for somnolence


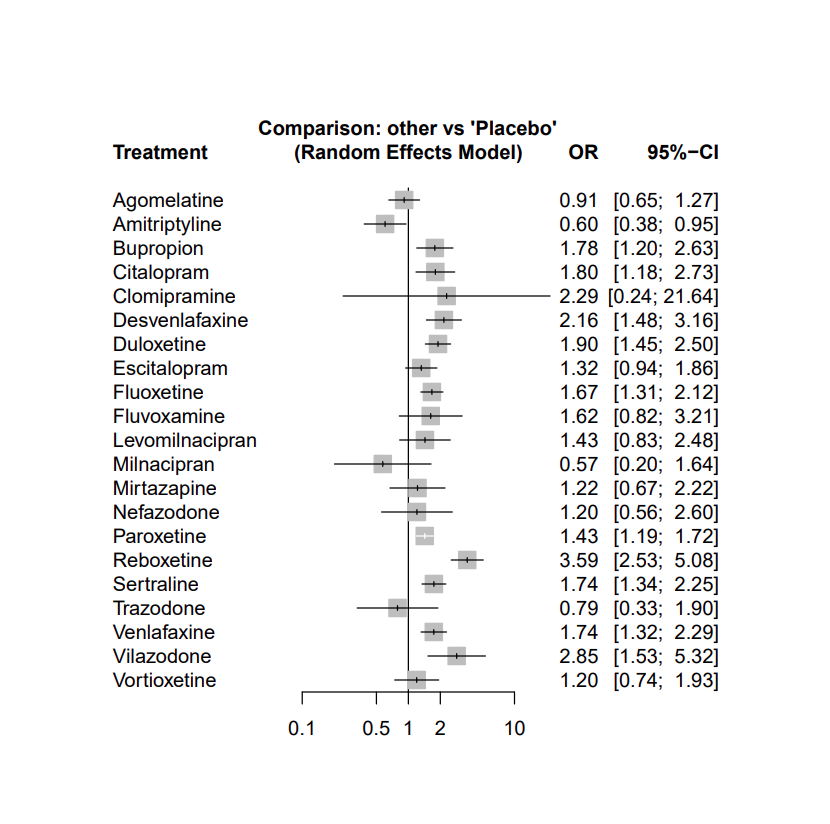


Figure S7 Result of sensitivity analyses for insomnia

References

1. A A-w, H A, L E, et al. Sertraline versus paroxetine in major depression: Clinical outcome after six months of continuous therapy. J Clin Psychopharmacol 2000;20:645-652.

2. E A, M C, Gb C, et al. Double-blind study of the efficacy and safety of sertraline versus fluoxetine in major depression. Int Clin Psychopharmacol 1993;8:197-202.

3. Https. //www.Gsk-clinicalstudyregister.Com/study/ak1102365#ps.

4. E A, V P, M D, et al. Active reference study of lu aa21004 in patients with major depressive disorder. Int J Neuropsychopharmacol 2012;15:589-600.

5. H A, S A, Sa J, et al. Comparison of mirtazapine and fluoxetine in the treatment of major depressive disorder: A double-blind, randomized trial. J Clin Pharmacy and Therapeutics 2005;30:133-138.

6. M A, R VF, C M, et al. Controlled comparison of two doses of milnacipran (f 2207) and amitriptyline in major depressive inpatients. Psychopharmacology 1989;98:163-168.

7. M A, R VF, Ma G, et al. Interest of a loading dose of milnacipran in endogenous depressive inpatients. Comparison with the standard regimen and with fluvoxamine. Eur Neuropsychopharmacol 1991;1:113-121.

8. M A, P D, A L, et al. Controlled comparison of nefazodone and amitriptyline in major depressive inpatients. Psychopharmacology 1994;115:254-260.

9. Asnis GM, Bose A, Gommoll CP, et al. Efficacy and safety of levomilnacipran sustained release 40 mg, 80 mg, or 120 mg in major depressive disorder: A phase 3, randomized, double-blind, placebo-controlled study. Journal of Clinical Psychiatry 2013;74:242-248.

10. Comparison of moclobemide, amitriptyline and placebo in depression: A canadian multicentre study. Psychopharmacology. Berl 1992.

11. D B, K B, C B. Resolution of sexual dysfunction during double-blind treatment of major depression with reboxetine or paroxetine. J Psychopharmacol 2006;20:91-96https//www.

12. Henigsberg N, Mahableshwarkar AR, Jacobsen P, et al. A randomized, double-blind, placebo-controlled 8-week trial of the efficacy and tolerability of multiple doses of lu aa21004 in adults with major depressive disorder. J Clin Psychiatry 2012;73:953-959.

13. Ta B, P G, E A, et al. Clinical efficacy of reboxetine: A comparative study with desipramine, with methodological considerations. 1998.

14. R B, M H, U R. Double-blind comparative study of paroxetine and amitriptyline in depressed patients of a university psychiatric outpatient clinic (pilot study). Neuropsychobiology 1985;13:31-37https//wwwgsk-clinicalstudyregistercom/study/29060/29309?

15. K B, J S, S M, et al. Mirtazapine orally disintegrating tablet versus sertraline: A prospective onset of action study. J Clin Psychopharmacol 2003;23:358-364.

16. Berlanga C, Arechavaleta B, Heinze G, et al. A double-blind comparison of nefazodone and fluoxetine in the treatment of depressed outpatients. Salud Mental 1997;20:1-8.

17. Bignamini A, Rapisarda V. A double-blind multicentre study of paroxetine and amitriptyline in depressed outpatients. Italian paroxetine study group. Int Clin Psychopharmacol 1992;6 Suppl 4:37-41.

18. R B, Nj S, N C, et al. The drug treatment of depression in general practice: A comparison of nocte administration of trazodone with mianserin, dothiepin and amitriptyline. Psychopharmacology 1988.

19. Andreoli V, Caillard V, Deo RS, et al. Reboxetine, a new noradrenaline selective antidepressant, is at least as effective as fluoxetine in the treatment of depression. Journal of Clinical Psychopharmacology 2002;22:393-399.

20. A B, D L, C G. Escitalopram in the acute treatment of depressed patients aged 60 years or older. Am J Geriatric Psychiatry 2008;16:14-20.

21. Bougerol T, Scotto JC, Patris M, et al. Citalopram and fluoxetine in major depression - comparison of two clinical trials in a psychiatrist setting and in general practice. Clinical Drug Investigation 1997;14:77-89.

22. Https://www.Iqwig.De/de/projekte-ergebnisse/studieninformationen-zureboxetin.3304.Html.

23. Boyer P, Montgomery S, Lepola U, et al. Efficacy, safety, and tolerability of fixed-dose desvenlafaxine 50 and 100 mg/day for major depressive disorder in a placebo-controlled trial. Int Clin Psychopharmacol 2008;23:243-253.

24. Ar B, L V, A B, et al. The sertraline vs. Electrical current therapy for treating depression clinical study: Results from a factorial, randomized, controlled trial. JAMA Psychiatry 2013;70:383-391.

25. Byerley WF, Reimherr FW, Wood DR, et al. Fluoxetine, a selective serotonin uptake inhibitor, for the treatment of outpatients with major depression. J Clin Psychopharmacol 1988;8:112-115.

26. [Available from: https://clinicaltrials.gov/ct2/show/record/NCT00463242.

27. Chouinard G. A double-blind controlled clinical trial of fluoxetine and amitriptyline in the treatment of outpatients with major depressive disorder. J Clin Psychiatry 1985;46:32-37.

28. G C, B S, Mc B, et al. Canadian multicenter, double-blind study of paroxetine and fluoxetine in major depressive disorder. J Affect Disord 1999;54:39-48https//wwwgsk-clinicalstudyregistercom/study/29060/29131#.

29. CL3-20098-022. Http://www.Ema.Europa.Eu/docs/en_gb/document_library/epar__public_assessment_report/human/000916/wc500038315.Pdf.

30. Http. //www.Ema.Europa.Eu/docs/en_gb/document_library/epar__public_assessment_report.

31. CL3-20098-026. Http://www.Ema.Europa.Eu/docs/en_gb/document_library/epar__public_assessment_report/human/000916/wc500038315.Pdf.

32. CL3-20098-062.

[Available from: https://www.clinicaltrialsregister.eu/ctr-search/search?query=2008-004642-92.

33. CL3-20098-070.

[Available from: https://www.clinicaltrialsregister.eu/ctr-search/trial/2009-011795-29/FI

34. Claghorn J GS, Goldstein BJ. Zimeldine tolerability in comparison to amitriptyline and placebo. Acta Psychiatr Scand Suppl 1983;308:104-114.

35. Claghorn JL, Lesem MD. A double-blind placebo-controlled study of org 3770 in depressed outpatients. J Affect Disord 1995;34:165-171.

36. Jl C, Cq E, Dd W, et al. Fluvoxamine maleate in the treatment of depression: A single-center, double-blind, placebo-controlled comparison with imipramine in outpatients. J Clin Psychopharmacol 1996;16:113-120.

37. Ge C, P R, A V-pJ. Double-blind comparison of venlafaxine and fluoxetine in patients hospitalized for major depression and melancholia. The Venlafaxine French Inpatient Study Group Int Clin Psychopharmacol 1994;9:139-143.

38. G C. Milnacipran/fluvoxamine study group. Antidepressant efficacy and tolerability of milnacipran, a dual serotonin and noradrenaline reuptake inhibitor: A comparison with fluvoxamine. Int Clin Psychopharmacol 2001;16:145-151.

39. Cohn JB, Wilcox C. A comparison of fluoxetine, imipramine, and placebo in patients with major depressive disorder. J Clin Psychiatry 1985;46:26-31.

40. Coleman CC CL, Foster VJ, et al. Sexual dysfunction associated with the treatment of depression: A placebo-controlled comparison of bupropion sustained release and sertraline treatment. Ann Clin Psychiatry 1999;11: 205–215.

41. Coleman CC KB, Bolden-Watson C, et al. Placebo-controlled comparison of the eff ects on sexual functioning of bupropion sustained release and fluoxetine. Clin Ther 2001;23:1040-1058.

42. Mh C, Aq D, Ce W, et al. Comparison of pramipexole, fluoxetine, and placebo in patients with major depression. Depress Anxiety 2000;11:58-65.

43. Corruble E dBC, Belaïdi C, Goodwin GM; agomelatine study group. Efficacy of agomelatine and escitalopram on depression, subjective sleep and emotional experiences in patients with major depressive disorder: A 24-wk randomized, controlled, double-blind trial. Int J Neuropsychopharmacol 2013;16:2219-2234.

44. Croft H SEJ, Houser T, Batey SR, Donahue RMJ, Ascher JA. A placebo-controlled comparison of the antidepressant efficacy and effects on sexual functioning of sustainedrelease bupropion and sertraline. Clin Ther 1999;21:643-658.

45. Ha C, N P, C G, et al. Efficacy and safety of vilazodone in major depressive disorder: A randomized, double-blind, placebocontrolled trial. J Clin Psychiatry 2014;75:e1291-1298.

46. Cunningham LA, Borison RL, Carman JS, et al. A comparison of venlafaxine, trazodone, and placebo in major depression. J Clin Psychopharmacol 1994;14:99-106.

47. La C. Once-daily venlafaxine extended release (xr) and venlafaxine immediate release (ir) in outpatients with major depression. Venlafaxine xr 208 study group. Ann Clin Psychiatry 1997;9:157-164.

48. P B, G G, Je T, et al. Mirtazapine and paroxetine in major depression: A comparison of monotherapy versus their combination from treatment initiation. Eur Neuropsychopharmacol 2009;19:457-465.

49. DeMartinis NA, Yeung PP, Entsuah R, et al. A double-blind, placebo-controlled study of the efficacy and safety of desvenlafaxine succinate in the treatment of major depressive disorder. J Clin Psychiatry 2007;68:677-688.

50. D DR, P R, M L, et al. Fluoxetine and amitriptyline in elderly depressed patients. A 10-week, double-blind study on course of neurocognitive adverse events and depressive symptoms. Arch Gerontology and Geriatrics 1998;1:125-140.

51. Detke MJ, Lu YL, Goldstein DJ, et al. Duloxetine, 60 mg once daily, for major depressive disorder: A randomized double-blind placebo-controlled trial. Journal of Clinical Psychiatry 2002;63:308-315.

52. Mj D, Cg W, Ch M, et al. Duloxetine in the acute and long-term treatment of major depressive disorder: A placebo- and paroxetine-controlled trial. Eur Neuropsychopharmacol 2004;14:457-470.

53. Dierick M, Ravizza L, Realini R, et al. A double-blind comparison of venlafaxine and fluoxetine for treatment of major depression in outpatients. Prog Neuropsychopharmacol Biol Psychiatry 1996;20:57-71.

54. S D, Sd H, Ks D, et al. Randomized trial of behavioral activation, cognitive therapy, and antidepressant medication in the acute treatment of adults with major depression. J Consult Clin Psychol 2006;74:658-159.

55. Doogan DP, Langdon CJ. A double-blind, placebo-controlled comparison of sertraline and dothiepin in the treatment of major depression in general practice. Int Clin Psychopharmacol 1994;9:95-100.

56. S D, Ma D, M J, et al. Study of the effects of ly2216684, a selective norepinephrine reuptake inhibitor, in the treatment of major depression. J Psychiatr Res 2010;44:356-363.

57. Dunbar GC, Claghorn JL, Kiev A, et al. A comparison of paroxetine and placebo in depressed outpatients. Acta Psychiatr Scand 1993;87:302-305.

58. L F, F A, M A, et al. Sertraline in major depression: Double-blind comparison with placebo. Biol Psychiatry 1995;38:592-602.

59. L F, L B, B Z, et al. Fluvoxamine vs imipramine and placebo: A double-blind comparison in depressed patients. Int Clin Psychopharmacol 1996;11:119-127.

60. We F, Jf R, Mw O, et al. Fluoxetine versus trazodone in depressed geriatric patients. J Geriatr Psychiatry Neurol 1989;2:208-214.

61. M F, Sl H, Ra J, et al. Acute efficacy of fluoxetine versus sertraline and paroxetine in major depressive disorder including effects of baseline insomnia. J Clin Psychopharmacol 2002;22:137-147.

62. Fava M, Alpert J, Nierenberg AA, et al. A double-blind, randomized trial of st john's wort, fluoxetine, and placebo in major depressive disorder. J Clin Psychopharmacol 2005;25:441-447.

63. Feighner JP, Boyer WF, Merideth CH, et al. A double-blind comparison of fluoxetine, imipramine and placebo in outpatients with major depression. Int Clin Psychopharmacol 1989;4:127-134.

64. Dunner DL DG. Optimal dose regimen for paroxetine. J Clin Psychiatry 1992;53.

65. V G, F K-c, R M, et al. Double-blind comparison of venlafaxine and amitriptyline in outpatients with major depression with or without melancholia. J Psychopharmacol 2000;14:61-66.

66. C G, Ch S, M M, et al. Multicenter double blind study of paroxetine and amitriptyline in elderly depressed inpatients. Psychopharmacology. Berl 1995;119:277-281.

67. Dj G, Y L, Mj D, et al. Duloxetine in the treatment of depression: A double-blind placebo-controlled comparison with paroxetine. J Clin Psychopharmacol 2004;24:389-399.

68. Cp G, Wm G, CA C. A randomized, double-blind, placebo-controlled study of flexible doses of levomilnacipran er (40–120 mg/day) in patients with major depressive disorder. Journal of Drug Assessment 2014;3:40-120.

69. K G, N A, R M, et al. Comparison of therapeutic effects of mirtazaine and citalopram on outpatients with major depressive disorder and anxiety symptoms. World Journal of Pharmaceutical Research 2015;4:346-357.

70. Griebel G BS, Stahl SM. The vasopressin v(1b) receptor antagonist ssr149415 in the treatment of major depressive and generalized anxiety disorders: Results from 4 randomized, double- blind, placebo-controlled studies. J Clin Psychiatry 2012;73:1403-1411.

71. Guelfi JD AM, Corruble E, Samuelian JC, Tonelli I, Tournoux A, Plétan Y. A double-blind comparison of the efficacy and safety of milnacipran and fluoxetine in depressed inpatients. Int Clin Psychopharmacol 1998;13:121-128.

72. A H, Rm C, C M, et al. Superior antidepressant efficacy results of agomelatine versus fluoxetine in severe mdd patients: A randomized, double-blind study. Int Clin Psychopharmacol 2010;25:305-314.

73. Ja H. Org 3770 (mirtazapine) versus trazodone. A Placebo Controlled Trial in Depressed Elderly Patients Human Psychopharmacology 1995.

74. Hewett K, Chrzanowski W, Schmitz M, et al. Eight-week, placebo-controlled, double-blind comparison of the antidepressant efficacy and tolerability of bupropion xr and venlafaxine xr. Journal of Psychopharmacology 2009;23:531-538.

75. Hewett K, Gee MD, Krishen A, et al. Double-blind, placebo-controlled comparison of the antidepressant efficacy and tolerability of bupropion xr and venlafaxine xr. J Psychopharmacol 2010;24:1209-1216.

76. Hicks JA, Argyropoulos SV, Rich AS, et al. Randomised controlled study of sleep after nefazodone or paroxetine treatment in out-patients with depression. British Journal of Psychiatry 2002;180:528-535.

77. Higuchi T, Kamijima K, Nakagome K, et al. A randomized, double-blinded, placebo-controlled study to evaluate the efficacy and safety of venlafaxine extended release and a long-term extension study for patients with major depressive disorder in japan. Int Clin Psychopharmacol 2016;31:8-19.

78. T H, Jp H, Hy J, et al. Paroxetine controlledrelease formulation in the treatment of major depressive disorder: A randomized, double-blind, placebo- controlled study. in Japan and Korea Psychiatry Clin Neurosci 2011;65:655-663.

79. L H, Lm O, S I. Cianopramine and amitriptyline in the treatment of depressed patients--a placebo-controlled study. Psychopharmacology. Berl 1985;86:205-208.

80. Hoyberg OJ, Maragakis B, Mullin J, et al. A double-blind multicentre comparison of mirtazapine and amitriptyline in elderly depressed patients. Acta Psychiatr Scand 1996;93:184-190.

81. Jw H, Tp S, Cy H, et al. Faster onset of antidepressant effects of citalopram compared with sertraline in drug-naïve first-episode major depressive disorder in a chinese population: A 6-week double-blind, randomized comparative study. J Clin Psychopharmacol 2011;31:577-581.

82. Itil TM SR, Mukherjee S, Coleman BS, Michael ST. A double-blind placebocontrolled study of fluvoxamine and imipramine in out-patients with primary depression. Br J Clin Pharmacol 1983;15(Suppl 3):433S-438S.

83. N I, Ka T, E H, et al. Efficacy and safety of desvenlafaxine 25 and 50mg/day in a randomized, placebo-controlled study of depressed outpatients. J Psychiatr Pract 2013;19:5-14.

84. Judd FK, Moore K, Norman TR, et al. A multicentre double blind trial of fluoxetine versus amitriptyline in the treatment of depressive illness. Aust N Z J Psychiatry 1993;27:49-55.

85. Kamijima K, Hashimoto S, Nagayoshi E, et al. Double-blind, comparative study of milnacipran and paroxetine in japanese patients with major depression. Neuropsychiatr Dis Treat 2013;9:555-565.

86. Kasper S, Olivieri L, Di Loreto G, et al. A comparative, randomised, double-blind study of trazodone prolonged-release and paroxetine in the treatment of patients with major depressive disorder. Curr Med Res Opin 2005;21:1139-1146.

87. S K, B E, K L, et al. Combining escitalopram with gaboxadol provides no additional benefit in the treatment of patients with severe major depressive disorder. Int J Neuropsychopharmacol 2012;15:715-725.

88. Katona C, Hansen T, Olsen CK. A randomized, double-blind, placebo-controlled, duloxetine-referenced, fixed-dose study comparing the efficacy and safety of lu aa21004 in elderly patients with major depressive disorder. Int Clin Psychopharmacol 2012;27:215-223.

89. D K, Rc B, S B, et al. A comparison of fluoxetine and amitriptyline in the treatment of major depression. Int Clin Psychopharmacol 1991;6:117-124.

90. M K, S M, W B, et al. Lack of efficacy of the substance p (neurokinin1 receptor) antagonist aprepitant in the treatment of major depressive disorder. Biol Psychiatry 2006;59:216-223.

91. Keller MB, Trivedi MH, Thase ME, et al. The prevention of recurrent episodes of depression with venlafaxine for two years (prevent) study: Outcomes from the 2-year and combined maintenance phases. J Clin Psychiatry 2007;68:1246-1256.

92 Kennedy SH, Avedisova A, Gimenez-Montesinos N, et al. A placebo-controlled study of three agomelatine dose regimens (10 mg, 25 mg, 25-50 mg) in patients with major depressive disorder. European Neuropsychopharmacology 2014;24:553-563.

93 A K, Gv U, Rl R, et al. The use of venlafaxine in the treatment of major depression and major depression associated with anxiety: A doseresponse study. Venlafaxine Investigator Study Group J Clin Psychopharmacol 1998;18:19-25.

94. Y K, W B, H S, et al. Efficacy and safety of bupropion sustainedrelease formulation for the treatment of major depressive disorder: A multi-center, randomized, double-blind, placebo-controlled study in asian patients. Neuropsychiatr Dis Treat 2013;9:1273-1280.

95. Ms K, N C, J F, et al. Distinct mechanism for antidepressant activity by blockade of central substance p receptors. Science 1998;281:1640-1645.

96. Cj K, He P, Kf O. Comparison of the tolerability and efficacy of citalopram and amitriptyline in elderly depressed patients treated in general practice. Depress Anxiety 1998;8:147-153.

97. V L, Pm A, Rp H, et al. Escitalopram versus citalopram and sertraline. A Double-Blind Controlled, Multi-centric Trial in Indian Patients with Unipolar Major Depression Indian J Psychiatry 2004;46:333-341.

98. Learned S, Graff O, Roychowdhury S, et al. Efficacy, safety, and tolerability of a triple reuptake inhibitor gsk372475 in the treatment of patients with major depressive disorder: Two randomized, placebo- and active-controlled clinical trials. J Psychopharmacol 2012;26:653-662.

99. Y L, M B, Ca M, et al. Efficacy of venlafaxine in depressive illness in general practice. Acta Psychiatr Scand 1997;95:485-493.

100. P L, L S, X X, et al. Once-daily duloxetine 60 mg in the treatment of major depressive disorder: Multicenter, double-blind, randomized, paroxetine-controlled, non-inferiority trial. in China, Korea, Taiwan and Brazil Psychiatry Clin Neurosci 2007;61:295-307.

101. P L, C G, E A. Improvement in subjective sleep in major depressive disorder with a novel antidepressant, agomelatine: Randomized, double-blind comparison with venlafaxine. J Clin Psychiatry 2007;68:1723-1732.

102. Lepine JP, Goger J, Blashko C, et al. A double-blind study of the efficacy and safety of sertraline and clomipramine in outpatients with severe major depression. Int Clin Psychopharmacol 2000;15:263-271.

103. UM L, H L, EH R. Escitalopram (10-20 mg/day) is effective and well tolerated in a placebo-controlled study in depression inprimary care. Int Clin Psychopharmacol 2003;18:211-217.

104. Lieberman DZ, Montgomery SA, Tourian KA, et al. A pooled analysis of two placebo-controlled trials of desvenlafaxine in major depressive disorder. Int Clin Psychopharmacol 2008;23:188-197.

105. Liebowitz MR MA, Padmanabhan SK, Ganguly R, Tummala R, Tourian KA. Efficacy, safety, and tolerability of desvenlafaxine 50 mg/day and 100 mg/day in outpatients with major depressive disorder. Curr Med Res Opin 2008;24(7):1877-1890.

106. Mr L, Ka T, E H, et al. Study 3362 investigators. A double-blind, randomized, placebo-controlled study assessing the efficacy and tolerability of desvenlafaxine 10 and 50 mg/day in adult outpatients with major depressive disorder. BMC Psychiatry 2013;22:94-94.

107. H L, A H, H Dh. Determination of the dose of agomelatine, a melatoninergic agonist and selective 5-ht(2c) antagonist, in the treatment of major depressive disorder: A placebo-controlled dose range study. Int Clin Psychopharmacol 2002;17:239-247.

108. Lydiard RB, Stahl SM, Hertzman M, et al. A double-blind, placebo-controlled study comparing the effects of sertraline versus amitriptyline in the treatment of major depression. J Clin Psychiatry 1997;58:484-491.

109. 047) MS. Https://www.Iqwig.De/de/projekte-ergebnisse/studieninformationen-zureboxetin.3304.Html.

110. Ar M, Pl J, Randomized CYA. A randomized, double-blind trial of 2.5 mg and 5 mg vortioxetine (lu aa21004) versus placebo for 8 weeks in adults with major depressive disorder. Curr Med Res Opin 2013;29:217-226.

111. Mahableshwarkar AR JP, Chen Y, Serenko M, Trivedi MH. A randomized, double-blind, duloxetine-referenced study comparing efficacy and tolerability of 2 fixed doses of vortioxetine in the acute treatment of adults with mdd. . Psychopharmacology (Berl) 2015;232:2061-2070.

112. P M, Y T, F J, et al. Escitalopram in major depressive disorder: A multicenter, randomized, double-blind, fixed-dose, parallel trial in a chinese population. Depression and Anxiety 2008;25:46-54.

113. Jj M, Sx X, J Z, et al. Rhodiola rosea versus sertraline for major depressive disorder: A randomized placebo-controlled trial. Phytomedicine 2015;15:394-399.

114. C M, A C, A R, et al. Anxious-agitated major depression 409. Responsive to fluoxetine? A double-blind comparison with amitriptyline. Pharmacopsychiatry 1998;31:216-221.

115. M M, C G, D C, et al. Efficacy and safety of vilazodone 20 and 40 mg in major depressive disorder: A randomized, double-blind, placebocontrolled trial. Int Clin Psychopharmacol 2015;30:67-74.

116. McGrath PJ, Stewart JW, Janal MN, et al. A placebo-controlled study of fluoxetine versus imipramine in the acute treatment of atypical depression. Am J Psychiatry 2000;157:344-350.

117. McPartlin GM RA, Anderson C, Casoy J. A comparison of once daily venlafaxine xr and paroxetine in depressed outpatients treated in general practice. Primary Care Psychiatry 1998;4:127-132.

118. Hj M, H B, F E, et al. Double-blind multicenter study of paroxetine and amitriptyline in depressed inpatients. Pharmacopsychiatry 1993;26:75-78.

119. Hj M, K G, F L, et al. Multicenter comparative study of sertraline versus amitriptyline in outpatients with major depression. Pharmacopsychiatry 2000;33:206-212.

120. Http://www.Accessdata.Fda.Gov/drugsatfda_docs/nda/98/020822a.Cfm.

121. Montgomery SA HA, Bothmer J. A randomised study comparing escitalopram with venlafaxine xr in primary care patients with major depressive disorder. Neuropsychobiology 2004;50(1):57-64.

122. Moon CA, Jago W, Wood K, et al. A double-blind comparison of sertraline and clomipramine in the treatment of major depressive disorder and associated anxiety in general practice. J Psychopharmacol 1994;8:171-176.

123. Moore N, Verdoux H, Fantino B. Prospective, multicentre, randomized, double-blind study of the efficacy of escitalopram versus citalopram in outpatient treatment of major depressive disorder. Int Clin Psychopharmacol 2005;20:131-137.

124. A M, Ca B, Jm E, et al. International collaborative group on sertraline in the treatment of outpatients with seasonal affective disorders. A placebo-controlled study of sertraline in the treatment of outpatients with seasonal affective disorder. Psychopharmacology. Berl 2004;171:390-397.

125. Munizza C, Olivieri L, Di Loreto G, et al. A comparative, randomized, double-blind study of trazodone prolonged-release and sertraline in the treatment of major depressive disorder. Curr Med Res Opin 2006;22:1703-1713.

126. MY-1008/BRL-029060/2/CPMS-076.

Https://www.Gsk-clinicalstudyregister.Com/study/29060/076#rs.

127. MY-1042/BRL-029060/CPMS-251.

Http://www.Gsk-clinicalstudyregister.Com/study/29060/251?Search=compound&compound=

paroxetine#rs

128. MY-1043/BRL-029060/115. Http://www.Gsk-clinicalstudyregister.Com/study/29060/115#rs

129. 128) M-B-P. Http://www.Gsk-clinicalstudyregister.Com/study/29060/128#rs

130. NCT01020799. Https://clinicaltrials.Gov/ct2/show/nct01020799.

131. NCT01145755. Https://clinicaltrials.Gov/ct2/show/nct01145755.

132. NCT01254305. Https://clinicaltrials.Gov/ct2/show/nct01254305.

133. NCT01255787. Https://clinicaltrials.Gov/ct2/show/nct01255787.

134. NCT01355081. Https://clinicaltrials.Gov/ct2/show/nct01355081.

135. NCT01808612. Https://clinicaltrials.Gov/ct2/show/nct01808612.

136. Cb N, P N, J B, et al. Double-blind multicenter comparison of fluvoxamine versus sertraline in the treatment of depressed outpatients. Depression 1995;3:163-169.

137. Nemeroff CB TMESG. A double-blind, placebo-controlled comparison of venlafaxine and fluoxetine treatment in depressed outpatients. J Psychiatry Res 2007;41:351-359.

138. Aa N, Jh G, Ch M. Duloxetine versus escitalopram and placebo in the treatment of patients with major depressive disorder: Onset of antidepressant action, a non-inferiority study. Curr Med Res Opin 2007;23:401-416.

139. R N, R A. Fluoxetine vs. Clomipramine in depressed patients: A controlled multicentre trial. J Affect Disord 1991;22:119-124.

140. Tm O, Al M, Lb M, et al. Assessment of depressive symptoms and functional outcomes in patients with major depressive disorder treated with duloxetine versus placebo: Primary outcomes from two trials conducted under the same protocol. Hum Psychopharmacol 2012;27:47-56.

141. Olie J, Gunn K, Katz E. A double-blind placebo-controlled multicentre study of sertraline in the acute and continuation treatment of major depression. Eur Psychiatry 1997;12:34-41.

142. Ou JJ XG, Wu RR, Li LH, Fang MS, Zhang HG, Xie SP, Shi JG, Du B, Yuan XQ, Zhao JP. Efficacy and safety of escitalopram versus citalopram in major depressive disorder: A 6-week, multicenter, randomized, double-blind, flexible-dose study. Psychopharmacology 2010;213(2):639–646.

143. 82/134) PH. Https://www.Gsk-clinicalstudyregister.Com/study/29060/314search=study&#rs.

144. (HP/82/47A) P. Https://wwwgsk-clinicalstudyregistercom/study/29060/316#rs.

145. (HP/82/64A) P. Https://www.Gsk-clinicalstudyregister.Com/study/29060/318#rs.

146. M P, Jm B, T B, et al. Citalopram versus fluoxetine: A double-blind, controlled, multicentre, phase iii trial in patients with unipolar major depression treated in general practice. International Clinical Psychopharmacology 1996;11:129-136.

147. Perahia DG, Pritchett YL, Kajdasz DK, et al. A randomized, double-blind comparison of duloxetine and venlafaxine in the treatment of patients with major depressive disorder. J Psychiatr Res 2008;42:22-34.

148. Ma Q-s, G H, P P, et al. Comparison of agomelatine and escitalopram on nighttime sleep and daytime condition and efficacy in major depressive disorder patients. International Clin 2011;26:252-262.

149. J R, Cg W, A S, et al. Efficacy of duloxetine on cognition, depression, and pain in elderly patients with major depressive disorder: An 8-week, double-blind, placebo-controlled trial. Am J Psychiatry 2007;164:900-909.

150. K R, E S, C C, et al. Nefazodone and imipramine in major depression: A placebo-controlled trial. Br J Psychiatry 1994;164:802-805.

151. Sp R, Ha S, Kr K, et al. Old-old depression study group. Antidepressant pharmacotherapy in the treatment of depression in the very old: A randomized, placebo-controlled trial. Am J Psychiatry 2004;161:2050-2059.

152. Rudolph RL FA. A double-blind, randomized, placebo-controlled trial of oncedaily venlafaxine extended release (xr) and fluoxetine for the treatment of depression. J Affect Disord 1999;56:171-181.

153. Aj R, R A, Jc G, et al. Comparative effects of nefazodone and fluoxetine on sleep in outpatients with major depressive disorder. Biol Psychiatry 1998;44:3-14.

154. E S, Gb C, G P, et al. Paroxetine versus amitriptyline in patients with recurrent major depression: A double-blind trial. Int J Psychiatry Clin Pract 2002;6:23-29.

155. Sambunaris A BA, Gommoll CP, Chen C, Greenberg WM, Sheehan DV. A phase iii, double- blind, placebo-controlled, flexible-dose study of levomilnacipran extendedrelease in patients with major depressive disorder. J Clin Psychopharmacol 2014;34:47-56.

156. Af S, C K, He R, et al. Mirtazapine vs. Paroxetine study group. Double-blind, randomized comparison of mirtazapine and paroxetine in elderly depressed patients. Am J Geriatr Psychiatry 2002;10:541-550.

157. Schatzberg, A. RS. A double-blind, placebo-controlled study of venlafaxine and fluoxetine in geriatric outpatients with major depression. Am J Geriatr Psychiatry 2006;14:361-370.

158. Schneider LS, Nelson JC, Clary CM, et al. An 8-week multicenter, parallel-group, double-blind, placebo-controlled study of sertraline in elderly outpatients with major depression. Am J Psychiatry 2003;160:1277-1285.

159. Schone W, Ludwig M. A double-blind study of paroxetine compared with fluoxetine in geriatric patients with major depression. J Clin Psychopharmacol 1993;13:34S-39S.

160. Https://www.Iqwig.De/download/studie_schwartz_et_al_poster.Pdf.

161. (NCT00668525) S-M-. Https://clinicaltrials.Gov/ct2/show/nct00668525.

162. Sechter D, Troy S, Paternetti S, et al. A double-blind comparison of sertraline and fluoxetine in the treatment of major depressive episode in outpatients. Eur Psychiatry 1999;14:41-48.

163. Sechter D, Vandel P, Weiller E, et al. A comparative study of milnacipran and paroxetine in outpatients with major depression. J Affect Disord 2004;83:233-236.

164. Ec S, Sm S, Sr B, et al. Safety profile of sustained579. Release bupropion in depression: Results of three clinical trials. Clin Ther 1999;21:454-580http//digitalcommonsohsuedu/fdadrug/421/.

165. Dv S, Ha C, Er G, et al. Trazodone in major depressive disorder: A randomized, double-blind, placebocontrolled study. Psychiatry Res 2009;6:20-33.

166. Sheehan DV CH, Gossen ER, Levitt RJ, Brullé C, Bouchard S, Rozova A. Extended-release trazodone in major depressive disorder: A randomized, double-blind, placebo-controlled study. Psychiatry (Edgmont) 2009;6(5):20-33.

167. L S, Ah S, Ys H, et al. Comparable efficacy and safety of 8 weeks treatment with agomelatine 25-50mg or fluoxetine 20-40 g in asian out-patients

with major depressive disorder. Asian J Psychiatr 2014;8:26-32.

168. Sramek JJ KK, Jasinsky O, Kardatzke D, Kennedy S, Cutler NR. Placebo-controlled study of abt-200 versus fluoxetine in the treatment of major depressive disorder. Depression 1995;3:199-203.

169. (CTN009-FCE20124) S. Https://www.Iqwig.De/de/projekte-ergebnisse/studieninformationen-zu-reboxetin.3304.Html

170. (M2020/0032) S. Https://www.Iqwig.De/download/studie_032.Pdf.

171. 015 S. Https://www.Iqwig.De/de/projekte-ergebnisse/studieninformationen-zu-reboxetin.3304.Html.

172. 043 S. Https://www.Iqwig.De/download/studie_043.Pdf.

173. 045 S. Https://www.Iqwig.De/de/projekte-ergebnisse/studieninformationen-zu-reboxetin.3304.Html

174. 049 S. Https://www.Iqwig.De/de/projekte-ergebnisse/studieninformationen-zu-reboxetin.3304.Html

175. B SFJ-M-H-SG. Http://www.Lillytrials.Com/results/cymbalta.Pdf (starting page in the pdf document: 147)

176. Thase ME CA, Haight BR, Thompson AH, Modell JG, Johnston JA. A doubleblind comparison between bupropion xl and venlafaxine xr: Sexual functioning, antidepressant efficacy, and tolerability. J Clin Psychopharmacol 2006;26:482-488.

177. Tollefson GD, Bosomworth JC, Heiligenstein JH, et al. A double-blind, placebo-controlled clinical trial of fluoxetine in geriatric patients with major depression. The fluoxetine collaborative study group. Int Psychogeriatr 1995;7:89-104.

178 .Ka T, Sk P, J G. Desvenlafaxine 50 and 100 mg/d in the treatment of major depressive disorder: An 8. Week, Phase III, Multicenter, Randomized, Double-Blind, Placebo- Controlled, Parallel Group Trial and a Post Hoc Pooled Analysis of Three Studies Clinical Therapeutics 2009;31:1405-1423.

179. M T, M G, I N, et al. Increased remission rates with venlafaxine compared with fluoxetine in hospitalized patients with major depression and melancholia. Int Clin Psychopharmacol 2000;15:29-34.

180. Vanmoffaert M, Bartholome F, Cosyns P, et al. A controlled comparison of sertraline and fluoxetine in acute and continuation treatment of major depression. Human Psychopharmacology-Clinical and Experimental 1995;10:393-405.

181. D V, Ep A, Gh S, et al. Escitalopram versus sertraline in the treatment of major depressive disorder: A randomized clinical trial. Current Medical Research and Opinion 2007;23:245-250.

182. M V, Ja O, G M, et al. Fluoxetine versus amitriptyline in the treatment of major depression with associated anxiety (anxious depression): A double-blind comparison. International Clinical Psychopharmacology 1993;8:143-149.

183. M V, M A, Double-blind CG. Placebo-controlled study with reboxetine in inpatients with severe major depressive disorder. J Clin Psychopharmacol 2000;20:28-34.

184. M V, R M, Cj R-vM, et al. Comparative efficacy antidepressants study group. Comparison of the effects of mirtazapine and fluoxetine in severely depressed patients. CNS Drugs 2005;19:137-146.

185. A W, Gm C, M A, et al. 24- week study comparing the efficacy and tolerability of mirtazapine and paroxetine in depressed patients in primary care. Int Clin Psychopharmacol 2003;18:133-141.

186. Wade A, Gembert K, Florea I. A comparative study of the efficacy of acute and continuation treatment with escitalopram versus duloxetine in patients with major depressive disorder. Curr Med Res Opin 2007;23:1605-1614.

187. Wang G, McIntyre A, Earley WR, et al. A randomized, double-blind study of the efficacy and tolerability of extended-release quetiapine fumarate (quetiapine xr) monotherapy in patients with major depressive disorder. Neuropsychiatr Dis Treat 2014;10:201-216.

188. G W, M G, G F, et al. Comparison of vortioxetine versus venlafaxine xr in adults in asia with major depressive disorder: A randomized, doubleblind study. Curr Med Res Opin 2015;31:785-794.

189. Rh W, Ja J, Cg L, et al. Comparison of bupropion and trazodone for the treatment of major depression. J Clin Psychopharmacol 1994;14:170-179.

190. AK140016 W. Https://www.Gsk-clinicalstudyregister.Com/study/ak140016#rs

191. Cs W, Jb C, Bb K, et al. A double- blind, placebo-controlled study comparing mianserin and amitriptyline in moderately depressed outpatients. Int Clin Psychopharmacol 1994;9:271-279.

192. Vy Y, Ai B, Yg Y, et al. Efficacy and tolerability of escitalopram versus citalopram in major depressive disorder: A 6-week, multicenter, prospective, randomized, double-blind, active-controlled study in adult outpatients. Clin Ther 2007;29:2319-2332.

193. J Z, A S, S S. Efficacy and safety of agomelatine in the treatment of major depressive disorder. J Clin Psychopharm 2010;30:135-144.

194. L Z, Ww X, Lh L. Efficacy and safety of prolonged-release trazodone in major depressive disorder. Pharmacology 2014;94:199-206.

195. 29060/299. Https://www.Gsk-clinicalstudyregister.Com/study/29060/299#rs

196. 29060/356. Https://www.Gsk-clinicalstudyregister.Com/study/29060/356#rs

197. Borhannejad F, Shariati B, Naderi S, et al. Comparison of vortioxetine and sertraline for treatment of major depressive disorder in elderly patients: A double-blind randomized trial. Journal of Clinical Pharmacy and Therapeutics 2020;45:804-811.

198. Emsley R, Ahokas A, Suarez A, et al. Efficacy of tianeptine 25-50 mg in elderly patients with recurrent major depressive disorder: An 8-week placebo- and escitalopram-controlled study. Journal of Clinical Psychiatry 2018;79.

199. Fagiolini A, Albert U, Ferrando L, et al. A randomized, double-blind study comparing the efficacy and safety of trazodone once-a-day and venlafaxine extended-release for the treatment of patients with major depressive disorder. International Clinical Psychopharmacology 2020:137-146.

200. Inoue T, Sasai K, Kitagawa T, et al. Randomized, double-blind, placebo-controlled study to assess the efficacy and safety of vortioxetine in japanese patients with major depressive disorder. Psychiatry and Clinical Neurosciences 2020;74:140-148.

201. Kennedy SH, Avedisova A, Belaïdi C, et al. Sustained efficacy of agomelatine 10 mg, 25 mg, and 25-50 mg on depressive symptoms and functional outcomes in patients with major depressive disorder. A placebo-controlled study over 6 months. European Neuropsychopharmacology 2016;26:378-389.

202. Nishimura A, Aritomi Y, Sasai K, et al. Randomized, double-blind, placebo-controlled 8-week trial of the efficacy, safety, and tolerability of 5, 10, and 20 mg/day vortioxetine in adults with major depressive disorder. Psychiatry and Clinical Neurosciences 2018;72:64-72.

203. Udristoiu T, Dehelean P, Nuss P, et al. Early effect on general interest, and short-term antidepressant efficacy and safety of agomelatine (25-50 mg/day) and escitalopram (10-20 mg/day) in outpatients with major depressive disorder. A 12-week randomised double-blind comparative study. Journal of Affective Disorders 2016;199:6-12.

204. Wang J, Liu XF, Feng C, et al. Efficacy and safety of vortioxetine for the treatment of major depressive disorder: A randomised double-blind placebo-controlled study. International Journal of Psychiatry in Clinical Practice 2019;23:245-250.

205. NCT04853407. A study to evaluate the efficacy and safety of ansofaxine hydrochloride extended-release tablets in the treatment of major depressive disorder (mdd). <https://clinicaltrialsgov/ct2/show/NCT04853407?id=NCT04853407&draw=2&rank=1&load=cart.>

206.LIN C H, WANG S H, LANE H Y. Effects of Sodium Benzoate, a D-Amino Acid Oxidase Inhibitor, on Perceived Stress and Cognitive Function Among Patients With Late-Life Depression: A Randomized, Double-Blind, Sertraline- and Placebo-Controlled Trial. Int J Neuropsychopharmacol, 2022, 25(7): 545-555.

207. NCT02623205. Advancing Personalized Antidepressant Treatment Using PET/MRI. https://clinicaltrials.gov/ct2/show/NCT02623205?term=NCT02623205&draw=2&rank=1

1. NCT02332291.Connectivity Affecting the Antidepressant REsponse Study (CAARE). https://clinicaltrials.gov/ct2/show/results/NCT02332291?term=NCT02332291&draw=2&rank=1
